# Nanocaged enzymes with enhanced catalytic activity and increased stability against protease digestion

**DOI:** 10.1038/ncomms10619

**Published:** 2016-02-10

**Authors:** Zhao Zhao, Jinglin Fu, Soma Dhakal, Alexander Johnson-Buck, Minghui Liu, Ting Zhang, Neal W. Woodbury, Yan Liu, Nils G. Walter, Hao Yan

**Affiliations:** 1 Center for Molecular Design and Biomimetics, the Biodesign Institute at Arizona State University, Tempe, Arizona 85287, USA; 2 School of Molecular Sciences, Arizona State University, Tempe, Arizona 85287, USA; 3 Department of Chemistry, Center for Computational and Integrative Biology, Rutgers University-Camden, Camden, New Jersey 08102, USA; 4 Department of Chemistry, Single Molecule Analysis Group, University of Michigan, Ann Arbor, Michigan 48109, USA; 5 Center for Innovations in Medicine, the Biodesign Institute at Arizona State University, Tempe, Arizona 85287, USA

## Abstract

Cells routinely compartmentalize enzymes for enhanced efficiency of their metabolic pathways. Here we report a general approach to construct DNA nanocaged enzymes for enhancing catalytic activity and stability. Nanocaged enzymes are realized by self-assembly into DNA nanocages with well-controlled stoichiometry and architecture that enabled a systematic study of the impact of both encapsulation and proximal polyanionic surfaces on a set of common metabolic enzymes. Activity assays at both bulk and single-molecule levels demonstrate increased substrate turnover numbers for DNA nanocage-encapsulated enzymes. Unexpectedly, we observe a significant inverse correlation between the size of a protein and its activity enhancement. This effect is consistent with a model wherein distal polyanionic surfaces of the nanocage enhance the stability of active enzyme conformations through the action of a strongly bound hydration layer. We further show that DNA nanocages protect encapsulated enzymes against proteases, demonstrating their practical utility in functional biomaterials and biotechnology.

## Abstract (web summary)

Cells compartmentalize enzymes for enhanced efficiency of their metabolic pathways. Here, the authors describe a self-assembly approach to construct DNA nanocaged enzymes for enhancing catalytic activity and stability, and observe an inversed correlation between the protein size and the activity enhancement.

## Graphical abstract

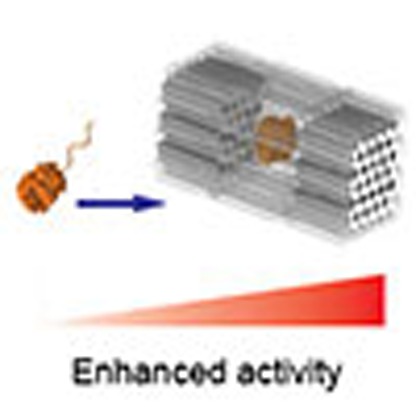

Common micro- and nanoscale subcellular compartments are formed from either lipids or proteins and include mitochondria, lysosomes, peroxisomes, carboxysomes and other metabolosomes, as well as multi-enzyme complexes^1^,^2^,^3^,^4^,^5^. Compartments increase the overall activity and specificity of the encapsulated enzyme pathways by maintaining a high local concentration of enzymes and substrates, promoting substrate channelling and protecting their content from damage, as well as by segregating potentially damaging reactions from the cytosol. Spatial confinement is also an important aspect for chaperone-assisted folding of linear polypeptides into active tertiary and quaternary conformations, as well as for preventing proteins from aggregating under cellular stress conditions^6^. A better understanding of the effects of spatial confinement on protein function will not only enhance our fundamental knowledge of cellular organization and metabolism but also increase our ability to translate biochemical pathways into a variety of noncellular applications, ranging from diagnostics and drug delivery to the production of high-value chemicals and smart materials^7^,^8^,^9^,^10^,^11^. Over the past few decades, artificial enzymatic particles have been created using compartmentalization by virus-like protein particles^7^, liposomes or polymersomes^5^ and chemical crosslinking^8^. However, severe obstacles to a broader application remain, including low encapsulation yield of large proteins because of steric hindrance^12^, insufficient access of substrates to the encapsulated enzymes, aggregation of vesicle shells^13^ and limited control over the spatial arrangement of proteins within the compartments^7^,^10^.

Recently, DNA nanostructures have started to emerge as promising molecular scaffolds to organize biomolecules at the nanoscale based on their programmable, sequence-driven self-assembly^14^,^15^,^16^,^17^,^18^,^19^,^20^. For example, multi-enzyme cascades have been assembled on DNA nanostructures with precise control over the spatial arrangement to enhance catalytic activity by substrate channelling^19^,^21^. Conversely, self-assembling DNA nanoboxes and -cages have shown promise in the delivery of macromolecular payloads such as antibodies^22^,^23^ and enzymes^24^. Tubular DNA nanostructures have also been used to construct efficient enzyme cascade nanoreactors^25^,^26^. Here, we describe a simple and robust strategy for the DNA nanocage-templated encapsulation of metabolic enzymes with high assembly yield and controlled packaging stoichiometry. With such an approach in hand, we sought to test the hypothesis that the recently described, chaperone-like stabilizing impact of polyphosphate on metabolic protein enzymes^27^ together with the cryptic RNA binding properties of many enzymes^28^ may lead to beneficial effects when enzymes are surrounded by DNA nanocages.

## Results

### Enzyme encapsulation strategy

**Figure 1:**
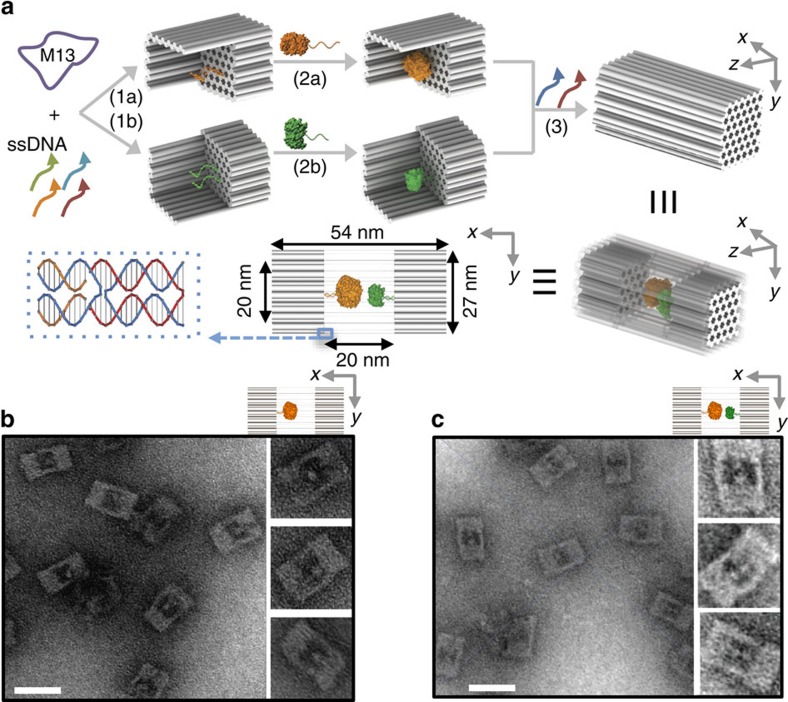
Design and characterization of DNA nanocage-encapsulated enzymes with controlled stoichiometry. (**a**) Schematic representations of the assembly of a DNA nanocage encapsulating a pair of GOx (orange) and HRP (green) enzymes. Individual enzymes were first attached to half-cages, followed by the addition of linker strands (red) to combine the two halves into a full-cage. Small pores of honeycomb shape (∼2.5 nm d.i.) were designed on the bottom of cages to facilitate the diffusion of substrate molecules in an out of the cage. (**b**) Negatively stained TEM images of DNA cages containing a single GOx (shown as less stained dots) and (**c**) a pair of GOx and HRP (shown as less stained dots). Scale bar, 50 nm.

As shown in Fig. 1a, our approach for enzyme encapsulation within DNA nanocages involves two steps: (1) the attachment of an individual enzyme into an open half-cage and (2) the assembly of two half-cages into a full (closed) nanocage. DNA half-cages were constructed by folding a full-length M13 viral DNA^29^ into the indicated shape based on a honeycomb lattice using the DNA origami technique^14^; a shape with two open sides was chosen to improve accessibility of the internal cavity to large proteins. Two half-cages were then linked into a full-cage by adding 24 short-bridge DNA strands that hybridize with the complementary ssDNA sequences extending from the edges of either half-cage. The DNA full-cage is ∼54 nm × 27 nm × 26 nm with designed inner cavity dimensions of 20 nm × 20 nm × 17 nm. By design, 42 small nanopores (each ∼2.5 nm in diameter) were introduced on each of the top and bottom surfaces of the DNA nanocage to permit the diffusion of small molecules (for example, enzyme substrates) across the DNA walls (Supplementary Fig. 1).

The formation of half and full DNA nanocages was first characterized using transmission electron microscopy (TEM) (Supplementary Figs 2 and 3) and gel electrophoresis (Supplementary Fig. 4), which indicate a nearly 100% yield for half-cages and a more than 90% yield for full-cages. To capture target enzymes into a half-cage, a previously reported succinimidyl 3-(2-pyridyldithio) propionate (SPDP) chemistry was used to crosslink a lysine residue on the protein surface to a thiol-modified oligonucleotide^19^,^30^,^31^. Two anchor probes of complementary sequence were displayed on the bottom of the half-cage cavity to capture a DNA-modified enzyme via sequence-specific DNA hybridization. As a demonstration of an enzyme cascade, a glucose oxidase (GOx)-attached half-cage was incubated with a horseradish peroxidase (HRP)-attached half-cage at a stoichiometric ratio of ∼1:1, followed by the addition of bridge strands into solution to assemble a full-DNA nanocage containing a GOx/HRP pair. The inner cavity of a full nanocage is of sufficient size to encapsulate this enzyme pair (GOx is ∼10 nm (ref. 32) and HRP ∼5 nm in diameter (ref. 33)). Unencapsulated enzyme and excess short DNA strands were removed using agarose gel electrophoresis^29^. Details of the enzyme–DNA conjugation and optimization of the assembly are discussed in Supplementary Figs 5–10, Supplementary Table 1 and Supplementary Note 1.

### Characterization of enzyme encapsulation

**Figure 2:**
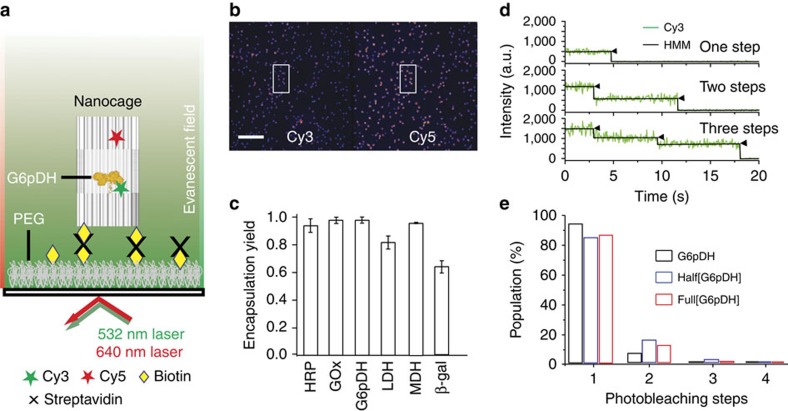
Single-molecule fluorescence characterization of enzyme encapsulation. (**a**) Schematic illustration of single-molecule fluorescence co-localization of Cy3-labelled protein with Cy5-labelled cage using TIRF microscopy. DNA cages were captured on the surface by biotin-streptavidin interaction. (**b**) Representative field of view of enzyme-encapsulating cages under TIRF microscope. Examples of Cy3-Cy5 co-localization are highlighted using a pair of rectangles. Scale bar, 10 μm. (**c**) Quantified encapsulation yield for six different enzymes. The total number of molecules analysed for each protein is shown in Supplementary Table 2. The error bars represent the standard deviation obtained from the analysis of two to four movies of the sample from the same batch. (**d**) Fluorophore photobleaching trajectories with one, two, and three photobleaching steps. Photobleaching steps were quantitatively analysed by fitting the trajectories by HMM in QUB program. (**e**) Photobleaching statistics for Cy3-labelled proteins encapsulated within half-cages (Half[G6pDH]) or full-cages (Full[G6pDH]), as well as for an unencapsulated protein control (G6pDH). HMM, hidden-Markov modelling.

To verify the presence of both enzymes within a DNA nanocage, the co-localization of a Cy3-labelled GOx (green emission) and a Cy5-labelled HRP (red emission) was quantified by dual-colour fluorescence gel electrophoresis, where a gel band with overlapped green and red colours was identified (see Supplementary Fig. 11). By comparison, the GOx-containing half-cage (Half[GOx]) shows the presence of only Cy3 (green), whereas a HRP-half-cage (Half[HRP]) shows the presence of only Cy5 (red). In addition, negatively stained TEM images were used to visualize DNA cages upon stoichiometrically controlled encapsulation of a single GOx (Fig. 1b) or a single GOx/HRP pair (Fig. 1c), where GOx and HRP were visible as brighter spots within the cage. To quantitatively analyse the yield of DNA nanocage encapsulation, two-colour total internal reflection fluorescence (TIRF) microscopy^34^ (Fig. 2a) was used to characterize the fluorescence co-localization of a Cy3-labelled enzyme and a Cy5-labelled nanocage (Fig. 2b). Six different enzymes were tested and characterized for encapsulation, ranging from the smallest HRP (∼44 kDa)^35^, (malic dehydrogenase (MDH, ∼70 kDa)^36^, glucose-6-phosphate dehydrogenase (G6PDH, ∼100 kDa)^37^, lactic dehydrogenase (LDH, ∼140 kDa)^38^ and GOx (∼160 kDa)^39^ to the largest β-galactosidase (β-Gal, ∼450 kDa)^40^. All six enzymes were successfully encapsulated within full DNA nanocages with high yields, ranging from 64 to 98% (Fig. 2c and Supplementary Table 2). The relatively low yield of β-Gal (64%) may be because of its large size (∼16 nm in diameter), which is comparable to the inner diameter of the nanocage (∼20 nm), likely resulting in steric hindrance for encapsulation. To evaluate how many copies of the same enzyme were encapsulated per DNA nanocage, the stepwise single-molecule fluorescence photobleaching was used to count the number of Cy3 fluorophores per cage (Fig. 2d). The number of copies of each enzyme per cage was estimated by normalizing the number of Cy3 fluorophores per DNA nanocage with the average number of Cy3 labels per free enzyme (see Supplementary Note 2). A majority of nanocage-encapsulated enzymes showed only one- or two-step photobleaching of Cy3, similar to the photobleaching of single free enzymes (Fig. 2e). These results suggest that most nanocages (∼90%) contain exactly one enzyme per cage, as expected (Fig. 2e and Supplementary Tables 3 and 4).

### Activity characterization of nanocaged enzymes

**Figure 3:**
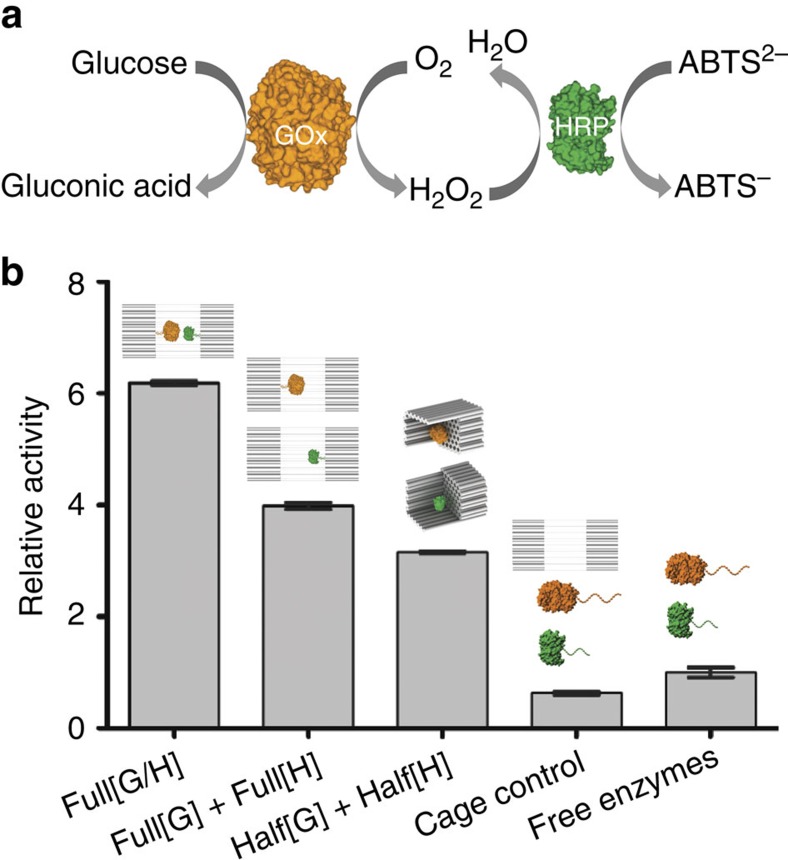
Activity characterization of encapsulated GOx/HRP pairs. (**a**) Schematic representation of the GOx/HRP cascade. (**b**) Normalized cascade activities for a GOx/HRP pair encapsulated within a full-cage (Full[GOx/HRP]), two individual full-cages (Full[GOx]+Full[HRP]) and two individual half-cages (Half[GOx]+Half[HRP]), as well as unencapsulaed enzyme pairs with and without the presence of DNA cages. Assay conditions: 1 nM enzyme or 1 nM enzyme-DNA cage, 1 mM glucose and 2 mM ABTS in TBS buffer (pH 7.5), and monitoring absorbance at 410 nm. Error bars were generated as the s.d. of at least three replicates.

To evaluate the effect of DNA nanocages on enzyme activity, we first tested an encapsulated GOx/HRP pair (Fig. 3a). This pair of enzymes catalyses a reaction cascade beginning with the oxidation of glucose by GOx to generate hydrogen peroxide (H_2_O_2_). H_2_O_2_ is subsequently used by HRP to oxidize ABTS, producing a strong colorimetric signal. As shown in Fig. 3b, the overall activity of a co-assembled GOx/HRP cage (Full[GOx/HRP]) is ∼8-fold higher than that of a control enzyme pair incubated with the same cage but without encapsulation. We hypothesized that two plausible effects could contribute to such a significant activity enhancement: (1) The proximity effect that brings the two enzymes close together and facilitates their substrate transfer, as described previously^19^,^21^,^41^; and/or (2) the unique environment provided by the high charge density of DNA helices within a nanocage. To separate the proximity effect from the charge density effect, we designed control experiments of DNA nanocages encapsulating only a single GOx or HRP enzyme, which clearly does not allow for substrate channelling between two proximal enzymes. For example, an equimolar mixture of two separate nanocages encapsulating either a single GOx or a single HRP (Full[GOx]+Full[HRP]) exhibited an ∼4-fold increase in overall activity compared with the unencapsulated control enzymes. Similarly, an equimolar mixture of two half-cages encapsulating either a single GOx or a single HRP already showed an increase in overall activity by ∼3-fold. Since there was no proximity effect in the case of two enzymes encapsulated into two different nanocages, the local environment modified by a DNA nanocage appears to be more important for the observed activity enhancement. Similarly, a half-cage was almost as effective in activity enhancement (3-fold) as a full-cage, suggesting that enzyme access to substrate does not play a role in this enhancement. Interestingly, a similar enhancement was reported previously upon conjugation of enzymes to a giant multi-branched DNA scaffold, without further explanation^42^.

**Figure 4:**
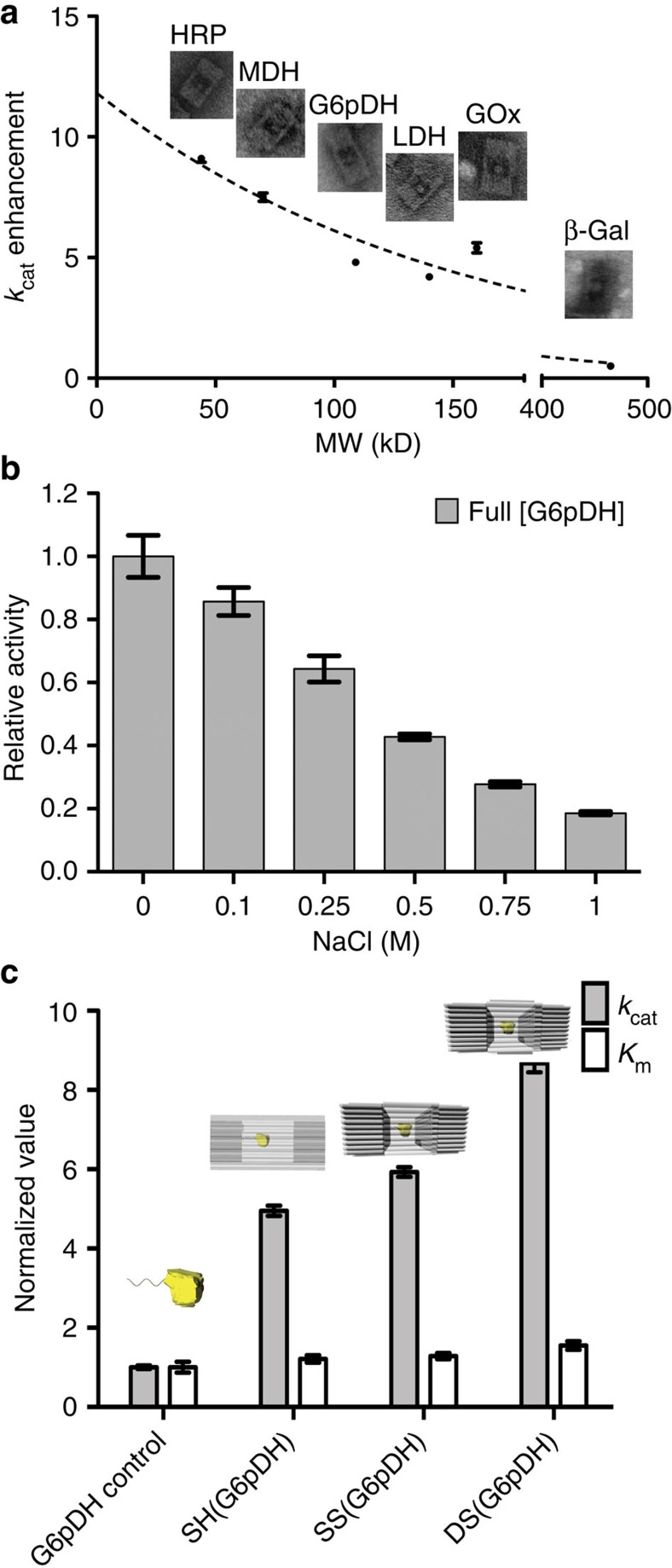
Mechanistic study of the activity enhancement of DNA nanocage-encapsulated enzymes. (**a**) Relationship between turnover rate enhancement factor after encapsulation against enzyme molecular weight (fitted using one-phase decay function). (**b**) Nanocage-encapsulated G6pDH activity change after incubation with different amount of NaCl. Assay conditions: 0.5 nM enzyme-DNA cage, incubation with 1 mM glucose-6-phosphate and 1 mM NAD^+^ in TBS buffer (pH 7.5), and monitoring absorbance at 340 nm. (**c**) Normalized *k*_cat_ and *K*_m_ values of free G6PDH and G6PDH that is encapsulated within different DNA cage: SH(G6pDH), a honeycomb lattice origami with a single layer; SS(G6pDH), a square-lattice origami with a single layer; and DS(G6pDH), a square-lattice origami with two layers. *k*_cat_ and *K*_m_ values of caged enzymes are normalized to that of free enzymes. Error bars were generated as the standard deviation of at least three replicates.

To test the generality of our observations, we evaluated the activity of six different enzymes upon encapsulation within DNA nanocages. As shown in Table 1, five of them (GOx, HRP, G6PDH, MDH and LDH) exhibited higher activity in nanocages than the free enzyme, with enhancements ranging from 3- to 10-fold. Detailed kinetic analyses show that the *K*_m_ (the Michaelis–Menten constant) varies little between encapsulated and free enzyme for most substrates (ranging from 0.5 to 2.4-fold of the free enzyme), suggesting that the porous DNA cages do not substantially hinder diffusion of small-molecule substrates. In contrast, a large increase in turnover number (*k*_cat_) was observed for these five enzymes (ranging from 3.5- to 9.6-fold of the free enzyme), suggesting an inherently higher catalytic activity of the proteins. For all the raw kinetics data and TEM images of the assembled structures, please see Supplementary Figs 12–46. Strikingly, an inverse correlation was observed between enhanced turnover and size of the encapsulated enzyme (Fig. 4a). That is, the smaller HRP (44 kDa) and MDH (70 kDa) exhibited relatively large increases in turnover number of 9.6±0.4- and 9.0±0.7-fold, respectively, whereas the larger enzymes G6PDH, LDH and GOx exhibited smaller enhancements of 4.7±0.1-, 4.1±0.1- and 5.4±0.2-fold, respectively. No correlation was observed between enhancement and isoelectric point (pI), despite the wide range of pI values for these enzymes (ranging from 4.2 to 10.0). In contrast to these five enzymes, β-Gal is strongly inhibited upon encapsulation, possibly because of its large size (∼16 nm in diameter, Supplementary Fig. 47) that is comparable to the inner cavity diameter (∼20 nm) of the DNA nanocage. Alternatively, the β-Gal orientation may be unfavourable and block binding of substrate to the active site. Notably, in a control experiment polyphosphate inhibited the activity of β-Gal (Supplementary Fig. 48), suggesting that the local high density of backbone phosphates of the DNA nanocage might be responsible for the decrease in activity of β-Gal. The DNA cages retained their structural integrity during the enzymatic reactions (Supplementary Fig. 49).

**Figure 5:**
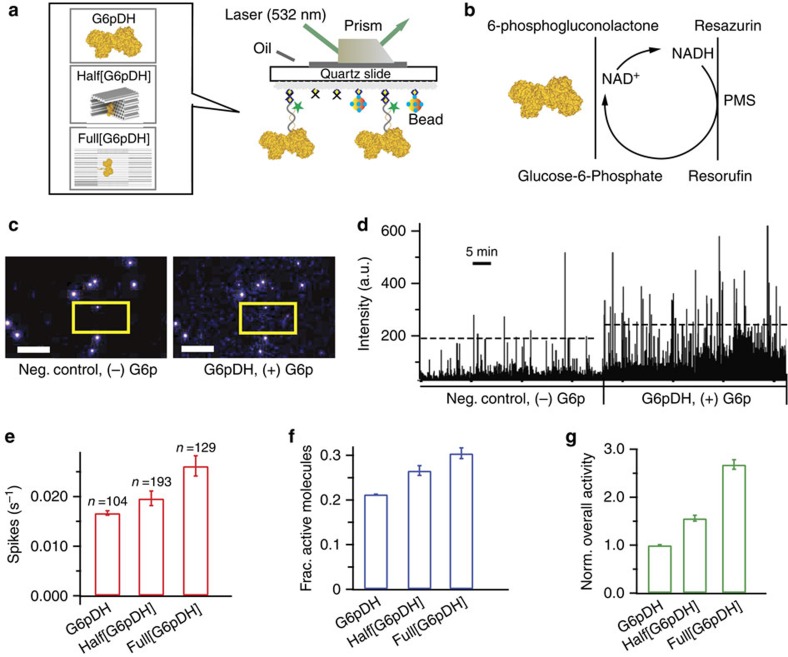
Single-molecule kinetics of nanocage-encapsulated enzymes. (**a**) Schematic of the experimental TIRF set up for characterizing G6pDH encapsulated within a full-cage (Full[G6pDH]) and a half-cage (Half[G6pDH]), as well as an unencapsulated control. (**b**) A PMS/resazurin-coupled fluorescence assay used to characterize the activity of G6pDH. NAD^+^ is first reduced to NADH by G6pDH, followed by PMS- catalyzed electron transfer from NADH to resazurin, producing a strongly fluorescent resorufin, which has an excitation/emission maximum at 544/590 nm. (**c**) TIRFM snapshots captured before and after the injection of substrate G6p. In presence of G6p, the field of view showed increased fluorescence due to the formation of resorufin (compare the boxed regions). Fluorescent beads (very bright spots present in both +G6p and −G6p images) were used as reference markers to correct for the drift. Scale bars, 10 μm. (**d**) Real-time traces of fluorescence spikes (resorufin production) for enzymes without and with the addition of G6p substrate. Ten single-molecule traces for each condition were concatenated. (**e**–**g**) Statistics of spike frequency, fraction of active molecules, and overall observed enzyme activity for G6pDH. The number of active molecules analysed is denoted by ‘*n*' in **e**. The standard deviations for the spike frequency were calculated after randomly assigning the active molecules into three groups; those for the fractions of active molecules were calculated from three to four independent movies, and those for the normalized overall activity were estimated from the propagation of errors. All experiments were carried out at room temperature in 1 × TBS buffer, pH 7.5, in the presence of 1 mM Mg^2+^ and 10% (w/v) PEG 8000.

To gain more detailed mechanistic insight into the enhancement of catalytic turnover, we applied a novel single-molecule fluorescence assay to characterize the activity of individual enzymes with and without encapsulation (Fig. 5). As shown in Fig. 5a,b, we used TIRF microscopy to record the repetitive turnover of substrates by individual G6pDH enzymes over time; coupling with a PMS/resazurin reaction^43^ (see single-molecule enzyme activity assay in Supplementary Note 3) allowed us to detect stochastic fluctuations of enzyme turnover rates via transient spikes in intensity from the generation of the fluorescent product resorufin (Fig. 5c,d and Supplementary Figs 50–52). Such fluctuations have been observed for various enzymes before^43^,^44^,^45^ and are thought to be induced by the conformational switching between more and less active sub-states^45^,^46^,^47^. Compared with a control without substrate, more frequent fluorescent spikes were observed with the addition of glucose-6-phosphate substrate (Fig. 5c,d). The average spike frequency was increased from 0.016±0.001 s^−1^ for unencapsulated enzymes, to 0.019±0.001 s^−1^ for the half-cage and 0.026±0.002 s^−1^ for the full-cage (Fig. 5e). Further analysis suggested that the fraction of active enzyme molecules was increased from ∼20% for unencapsulated enzymes to ∼27% for the half-cage and ∼31% for the full-cage (Fig. 5f). Taken together, the 1.6-fold higher spike frequency and the 1.5-fold increase in the fraction of active enzymes yield an ∼2.5-fold increase in G6pDH activity for the encapsulated compared with the unencapsulated enzyme (Fig. 5g), comparable to the ∼4-fold enhancement observed in the bulk assay. Conversely, a similar analysis of β-Gal activity showed an ∼3-fold lower activity of the full-cage enzyme (2.3±0.5-fold lower in spike frequency compared with free enzyme whereas the fractions of active enzymes (∼65%) were similar) compared with unencapsulated enzyme (Supplementary Fig. 51), also consistent with the bulk measurement.

The activity enhancement for DNA cage-encapsulated enzymes is consistent with recent reports of enhanced enzyme activity upon attachment to a long double-stranded DNA molecule (λDNA)^42^, a two-dimensional rectangular DNA origami^48^, or a DNA scaffold that bound to enzyme substrates^49^,^50^, and further suggests that it may be a widespread effect of enzyme–DNA interactions. Several mechanisms have been previously proposed to explain these observed enhancements, including micro-environment composed of giant and ordered DNA molecules, molecular crowding and the substrates affinity to DNA scaffolds. We further suggested that the negatively charged phosphate backbones of DNA might also contribute to the activity enhancement. DNA is a negatively charged biopolymer because of its closely spaced backbone phosphates (leading to a linear negative charge density of ∼0.6 e Å^−1^). Thus, upon encapsulation within a DNA nanocage, an enzyme is exposed to an environment full of negative charges that may resemble the relative abundance of polyanionic molecules and surfaces (including RNA and phospholipid membranes) within the cell. Phosphate is a known kosmotropic anion that increases the extent of hydrogen-bonded water structures (termed high-density or structured water)^51^,^52^,^53^. A DNA nanocage is thus expected to attract a strongly bound hydration layer of hydrogen-bonded water molecules inside its cavity^54^,^55^. Multiple studies^56^,^57^,^58^ have described that proteins are more stable and active in a highly ordered, hydrogen-bonded water environment, possibly due to stabilization of the hydrophobic interactions of a folded protein through an increase in the solvent entropy penalty upon unfolding. Consistent with this model, polyphosphate has been shown to act as a generic chaperone stabilizing a variety of enzymes^59^. To further test whether this mechanism is at work in our nanocages, we titrated the concentration of NaCl (known to consist of chaotropic ions)^60^,^61^ for the purpose of interrupting hydrogen-bonded water molecules. Consistent with our hypothesis, the activity of encapsulated enzymes significantly decreased with increasing NaCl concentration (reduced to ∼25% activity with 1 M NaCl as shown in Fig. 4b. A high concentration of Na^+^ can shield the negative charge on the DNA surface, thus disrupting the surface-bound hydration layer. As a control, we observed that the bulky kosmotropic cation, triethylammonium, had a much less pronounced effect on enzymatic activity (Supplementary Fig. 53). This model also allowed us to rationalize why we observed smaller enzymes to be more activated than larger enzymes, because their higher surface-to-volume ratio predicts a stronger impact of the hydration layer.

To further test this model, we investigated the effect of DNA helix density on the encapsulated enzyme activity. As shown in Fig. 4c, we designed three nanocages with walls that systematically increase the density of DNA helices, including: (1) a single-layer honeycomb pattern (SH) with ∼2–3 nm pores between helices; (2) a single-layer square pattern (SS) with smaller ∼0.5–1 nm pores between helices; and (3) a double-layer square pattern (DS). The helix density at the top and bottom surfaces thus increased from 0.12 helices per nm^2^ for SH to 0.16 helices per nm^2^ for the SS and DS designs. The *k*_cat_ of G6pDH encapsulated in the SH-cage was ∼4.7-fold higher than that of the free enzyme. As the density of DNA helices was increased, the *k*_*cat*_ of encapsulated G6pDH raised to ∼6-fold for the SS-cage and 8-fold for the DS-cage compared with the free enzyme control. A slight increase in *K*_m_ values was also observed from the SH-cage to the SS- and DS-cages, possibly due to a decrease in substrate diffusion through the DNA walls of these more tightly packed structures. For example, the *K*_m_ value of G6PDH increased from ∼410 μM in the SH-cage to ∼440 μM in the SS-cage and ∼530 μM in the DS-cage (Fig. 4c, Supplementary Fig. 54). Additional studies showed that activities of attached enzymes were enhanced by increasing the helix packing density for various one-, two- and three-dimensional DNA scaffolds (Supplementary Fig. 55). These observations suggest that encapsulated enzymes exhibit higher activity within densely packed DNA cages, consistent with our model that the highly ordered, hydrogen-bonded water environment near closely spaced phosphate groups are responsible for this effect.

### Nanocaged enzymes are protected from proteolysis

**Figure 6:**
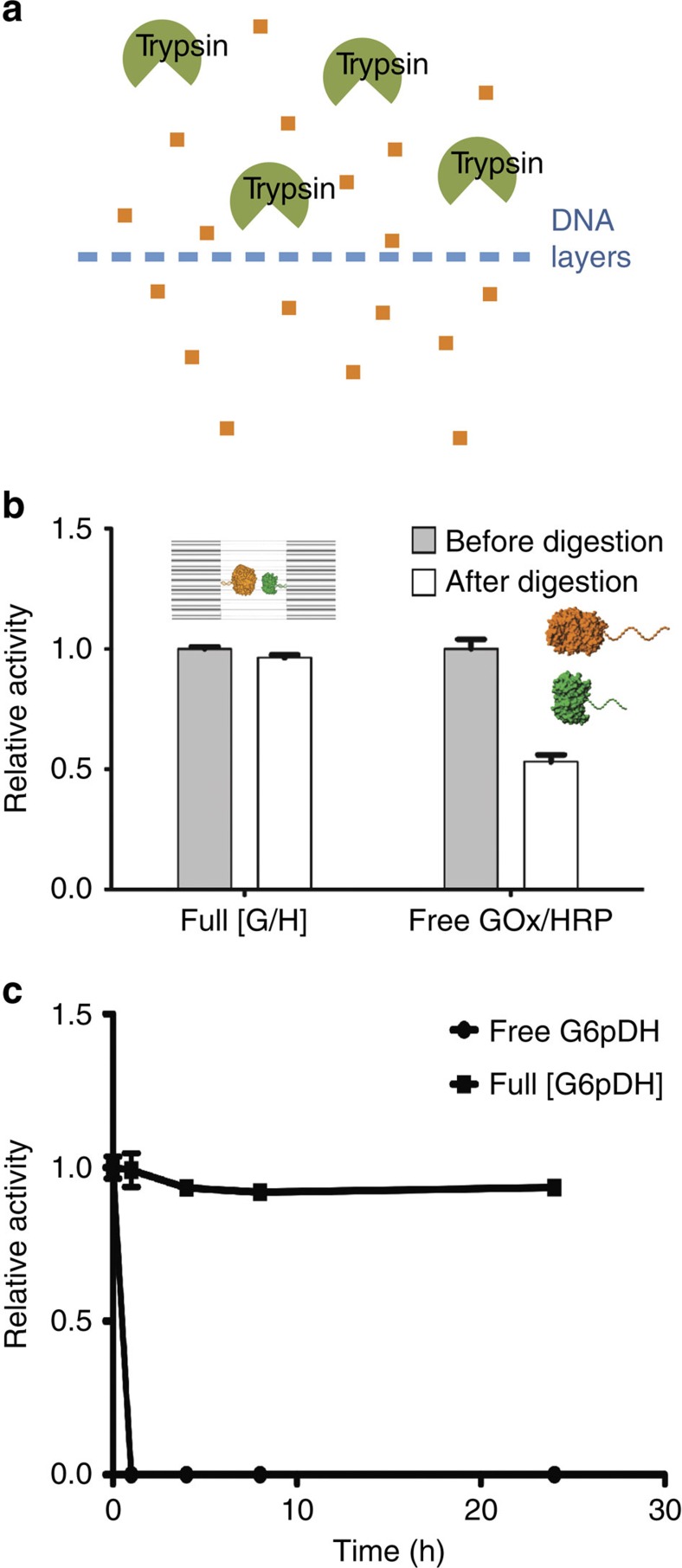
Protection of nanocaged enzymes against protease-mediated degradation and aggregation. (**a**) Schematic representation illustrating how a DNA cage may block access of big proteins such as a protease to the interior of the cage, but still allow the penetration of small molecules. (**b**) Relative enzyme activity of encapsulated GOx/HRP pairs (Full[GOx/HRP]) and free GOx/HRP pairs (free GOx/HRP) before and after the addition of trypsin. Trypsin digestion conditions: enzyme or enzyme-DNA cage was incubated with 1,000 times excess trypsin for 24 h at 37 °C. Assay conditions: 0.5 nM enzyme or 0.5 nM enzyme-DNA cage, incubation with 1 mM glucose and 2 mM ABTS in 1 × TBS buffer (pH 7.5), and monitoring absorbance at 410 nm. (**c**) Relative activity data for free G6pDH and Full[G6pDH] (0.5 nM) with trypsin digestion for 0, 1, 4, 8 and 24 h. Digestion by incubation sample with 1,000 times amount of trypsin at 37 °C in 1 × TBS buffer (pH 7.5). Error bars were generated as the s.d. of at least three replicates.

Self-assembled DNA nanostructures previously were found to be more resistant against nuclease degradation than single- or double-stranded DNA molecules^62^,^63^. Similarly, DNA nanocages should protect encapsulated enzymes from deactivation and aggregation under challenging biological conditions. As shown in Fig. 6, encapsulated GOx/HRP was highly resistant to digestion by trypsin (Fig. 6b), and retained more than 95% of its initial activity after incubation with trypsin for 24 h (Fig. 6b). A time-course experiment was also performed to demonstrate the stability of caged enzymes against trypsin digestion (Fig. 6c, Supplementary Figs 56–59). In contrast, free GOx/HRP only retained ∼50% of its initial activity after a similar incubation with trypsin. This result demonstrated the potential utility of DNA nanocages for protecting encapsulated proteins from biological degradation.

## Discussion

In summary, we have developed a method for using a DNA nanocage to efficiently encapsulate enzymes with high yield. Using single-molecule characterization, we were able to quantify the copies of encapsulated enzymes per cage with demonstrated one enzyme per cage. Upon encapsulation, five of six tested metabolic enzymes exhibit turnover numbers 4–10-fold higher than that of the free enzyme. Conversely, the *K*_m_ values remain similar between encapsulated enzymes and free enzymes, indicating an uninterrupted diffusion of small-molecule substrates and products through the nanopores in the DNA cage. Application of a novel single-molecule enzyme assay showed that both the fraction of active enzyme molecules and their individual turnover numbers increase as a consequence of encapsulation. We therefore propose that the unique local environment created within a DNA nanocage, particularly the high density of negatively charged phosphate groups, enhances the activity of encapsulated enzymes, where the tightly bound, highly structured water layers on DNA surface may stabilize the active enzyme conformations. This effect appears consistent with recent independent evidence that many conserved metabolic enzymes are stabilized by polyphosphate and associate non-specifically with nucleic acids through cryptic binding sites^27^,^28^,^64^, thus taking advantage of the high polyanionic DNA and RNA contents of the cell. DNA nanocages therefore may serve as a molecular tool to precisely sculpt the properties of the local environment of enzymes in smart-material and biotechnological application. DNA nanocages also demonstrated their value in protecting encapsulated enzymes from biological degradation through proteases. In the future, it may be feasible to construct precisely controlled, programmable DNA nanocages for theranostic nanodevices as therapeutic agents.

## Methods

### The design and characterization of DNA half-cages and full-cages

DNA origami half-cage and structures were designed with caDNAno (www.cadnano.org), each used one M13mp18 ssDNA as the scaffold. Detailed design schemes and DNA sequences are shown in the Supplementary Figs 1, 60 and 61. One or both of the half-cages contained single-stranded probe strands (four in each half-cage) extended towards the inside of the cage for binding with the DNA conjugated enzymes. Two of the half-cages can be linked together to form a fully enclosed full-cage though 24 linker strands. To form each of the half-cages, the M13mp18 ssDNA was mixed with the corresponding staples at a 1:10 molar ratio in 1 × TAE-Mg^2+^ buffer (40 mM Tris, 20 mM acetic acid, 2 mM EDTA and 12.5 mM magnesium acetate, pH 8.0) and annealed from 80 to 4 °C for 37 h. The excess staple strands were removed by the filtration of the DNA cages solution using 100 kDa Amicon filter with 1 × TAE-Mg^2+^ buffer for three times. To form a full-cage, 24 single-stranded DNA linkers were incubated with the two purified half-cages at a molar ratio of 5:1 for three hours at room temperature, in order to connect the two half-cages together.

### Enzyme-DNA cage assembly

A 15-fold molar excess of oligonucleotide-conjugated enzyme was incubated with the DNA half-cage containing capture strands. Protein assembly was performed using an annealing protocol, in which the temperature was gradually decreased from 37 to 4 °C over 2 h and then held constant at 4 °C using an established procedure^30^,^31^. Two Enzyme-attached half-cages were then assembled into a full-cage by adding DNA linkers as described above. The DNA caged-enzymes were further purified by agarose gel electrophoresis to remove excess free enzymes (please see Supplementary Notes 1 and 4 for detailed information).

### Single-molecule fluorescence microscopy

All single-molecule measurements were performed at room temperature using a TIRF microscope on PEGylated fused silica microscope slides. To passivate the microscope slides and functionalize the surface with biotin for selective immobilization of nanocages, a biotin- and PEG-coated surface was prepared by silylation with APTES, followed by incubation with a 1:10 mixture of biotin-PEG-SVA 5k:mPEG-SVA 5k as described previously^31^. A flow channel was constructed as described elsewhere^31^. To prepare the surface for enzyme or nanocage binding, a solution of 0.2 mg ml^−1^ streptavidin in T50 buffer (50 mM Tris-HCl, pH 8.0, 50 mM NaCl and 1 mM EDTA) was injected in to the flow channel, incubated for 10 min, and the excess streptavidin was flushed out thoroughly first with T50, then with 1 × TAE-Mg^2+^. For more detailed information, please see the Supplementary Notes 2 and 3.

### Bulk solution enzyme assay

A 96-well plate reader was used to monitor enzyme activity through absorbance changes of the samples. The enzyme samples and substrates were loaded in the wells of the 96-well plate with a final concentration of caged enzymes∼0.5 nM in 1 × TBS (Tris buffered saline with 1 mM MgCl_2_, pH 7.5) for most assays. Detailed assay conditions are described in the Supplementary Methods.

## Additional information

**How to cite this article:** Zhao, Z. *et al*. Nanocaged enzymes with enhanced catalytic activity and increased stability against protease digestion. *Nat. Commun.* 7:10619 doi: 10.1038/ncomms10619 (2016).

## Supplementary Material

Supplementary Supplementary Figures 1-61, Supplementary Tables 1-4, Supplementary Notes 1-4, Supplementary Methods and Supplementary References

## Figures and Tables

**Table 1:** Enzyme kinetic data for each individual enzyme encapsulated inside a DNA full-cage in comparison with the values for the free enzymes in solution.

Enzyme	pI	Molecular weight	Substrate	Free enzymes		Encapsulated enzymes	
				*K*_m_ (μM)	*k*_cat_ (s^−1^)	*K*_m_ (μM)	*k*_cat_ (s^−1^)
GOx	4.2	160 kDa	Glucose	6,200±900	240±10	3,000±600	1,300±50
HRP	8.8	44 kDa	H_2_O_2_	2.3±0.5	32±1	4.3±0.6	290±5
			ABTS	2,600±400	59±5	2,500±200	560±20
G6pDH	4.3	100 kDa	Glucose-6-phosphate	220±20	130±3	310±30	460±10
			NAD^+^	510±50	100±3	590±40	480±10
MDH	10.0	70 kDa	NADH	180±50	51±5	270±50	460±30
LDH	5.0	140 kDa	NADH	7.2±1.3	46±2	17.0±1.5	190±5
β-Gal	4.1	465 kDa	RBG	58.7±16.0	8.5±0.6*	95.5±18.9	1.6±0.1*

ABTS, 2,2′-azino-bis(3-ethylbenzothiazoline-6-sulphonic acid); GOx, glucose oxidase; G6pDH, glucose-6-phosphate dehydrogenase; HRP, horseradish peroxidase; LDH, lactic dehydrogenase; MDH, malic dehydrogenase; pI, isoelectric point.

The Michaelis–Menten plots of each enzyme and the conditions of the enzyme activity measurements can be found in the Supplementary Figs. The pI values of the enzymes were obtained from brenda-enzymes.org (refs 65, 66, 67, 68).

^*^k_cat_ values for β-Gal groups were not calibrated.
