# Supplementary material for: Nanocaged enzymes with enhanced catalytic activity and increased stability against protease digestion
Source: Nat Commun. 2016 Feb 10;7:10619. doi: 10.1038/ncomms10619 (PMC4749968; doi:10.1038/ncomms10619)
Supplement: Supplementary — Figures 1-61, Supplementary Tables 1-4, Supplementary Notes 1-4, Supplementary Methods and Supplementary References [file ncomms10619-s1.pdf]

Supplementary Figures

Cross-sectional view

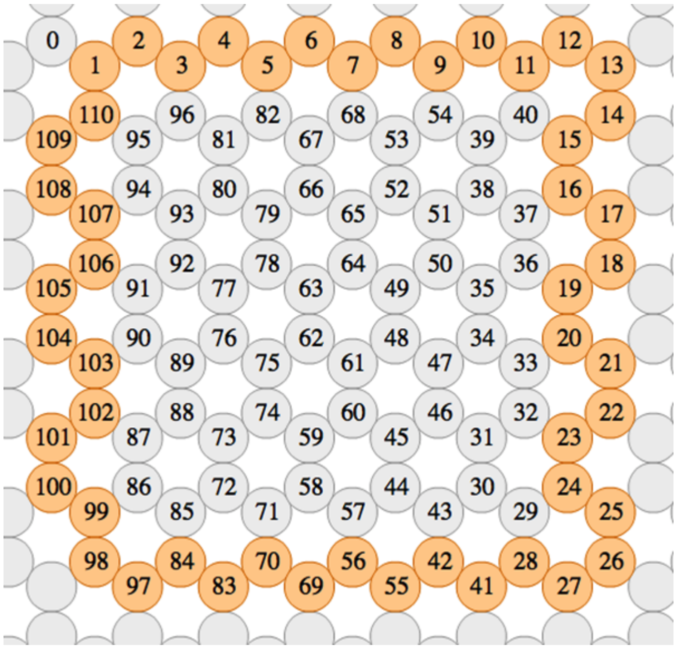

3D View

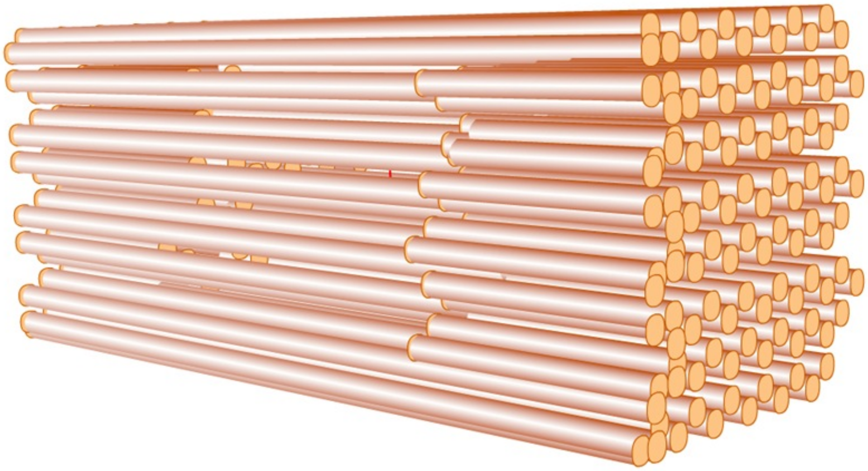

Supplementary Figure 1: Design of SH full-cage (honeycomb lattice), including cross-sectional view and 3D view.

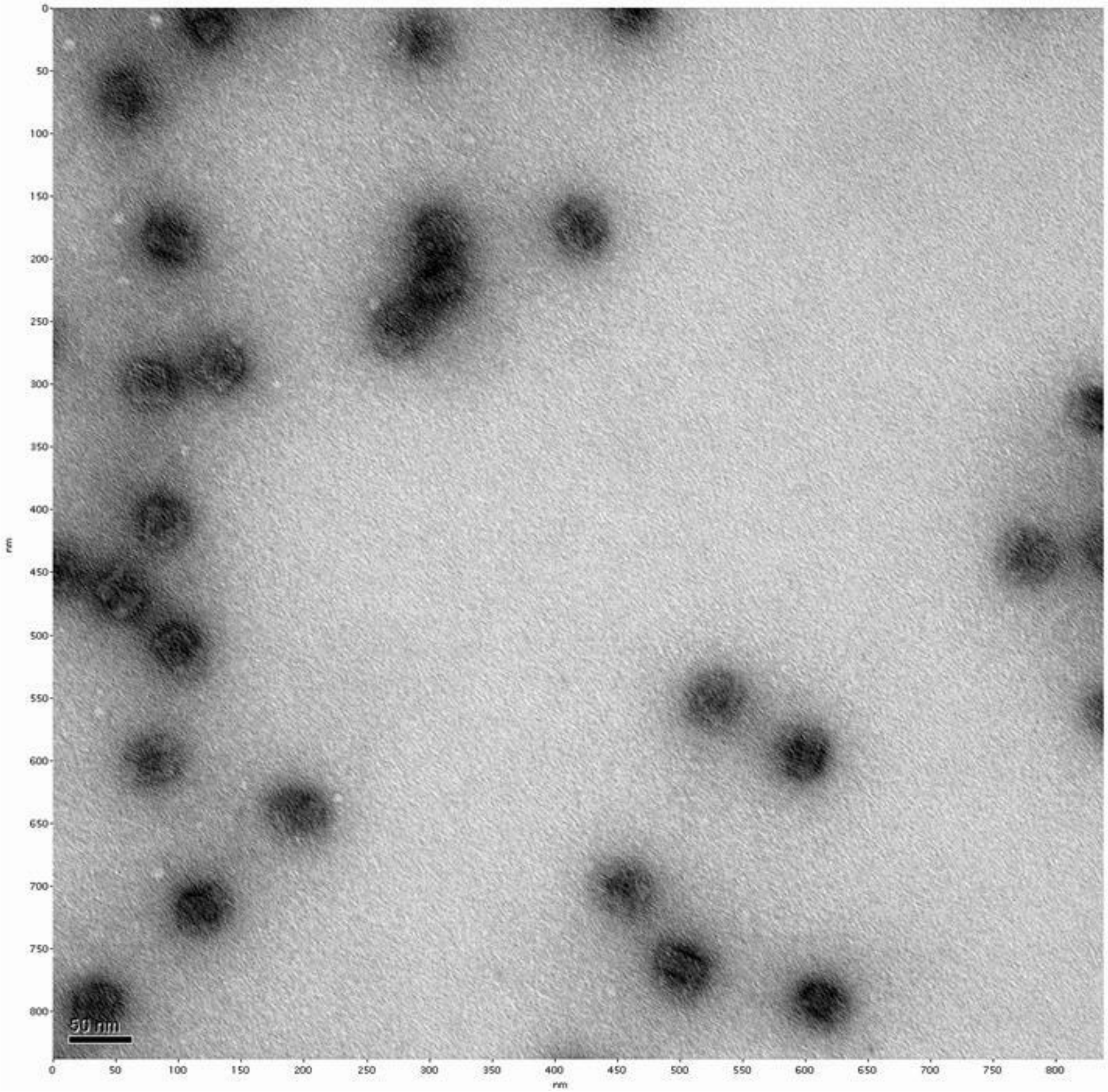

Supplementary Figure 2: A representative TEM image of the half-cage structure (scale bar: 50 nm).

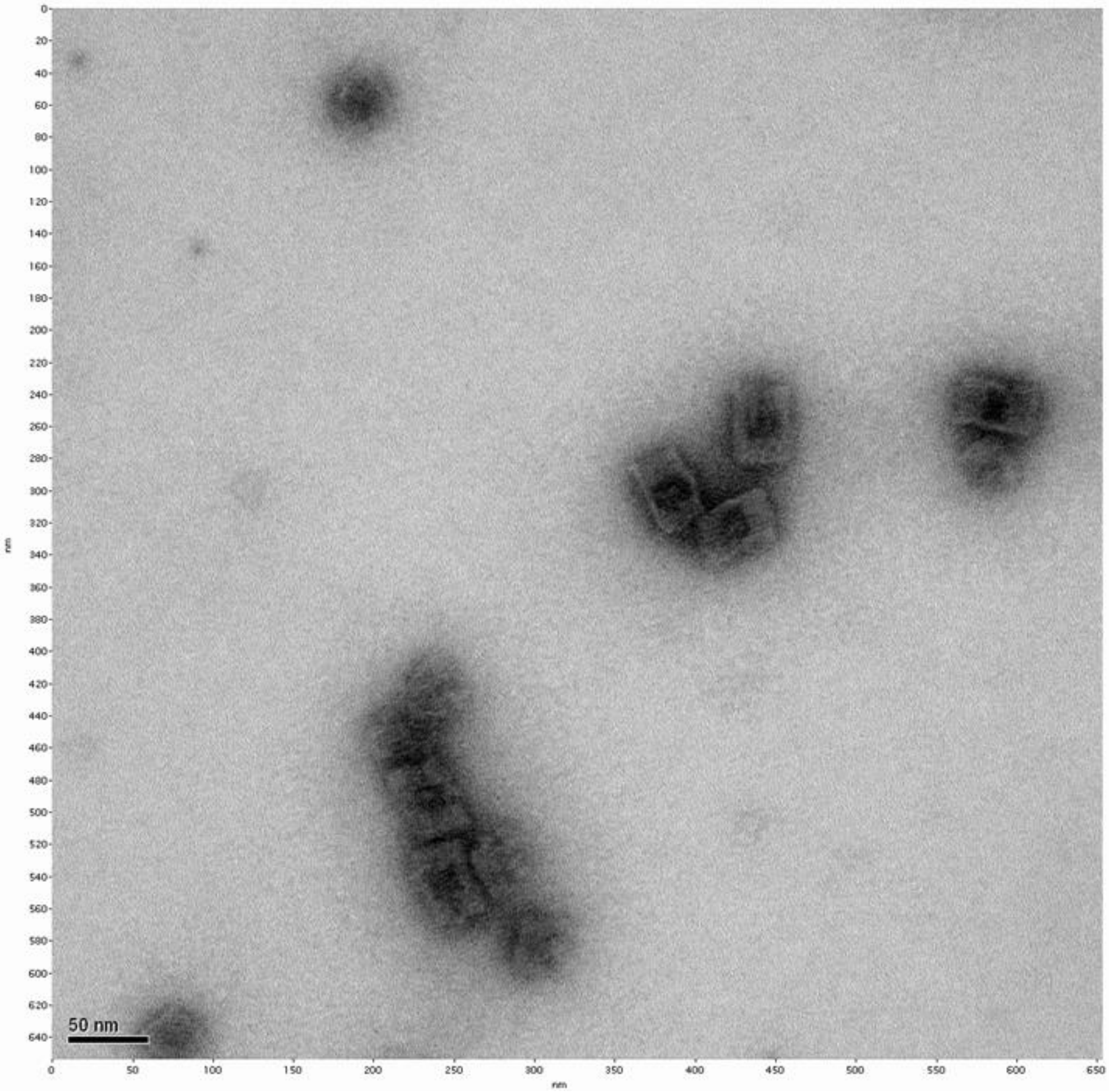

Supplementary Figure 3: A representative TEM image of the full-cage structure (scale bar: 50 nm).

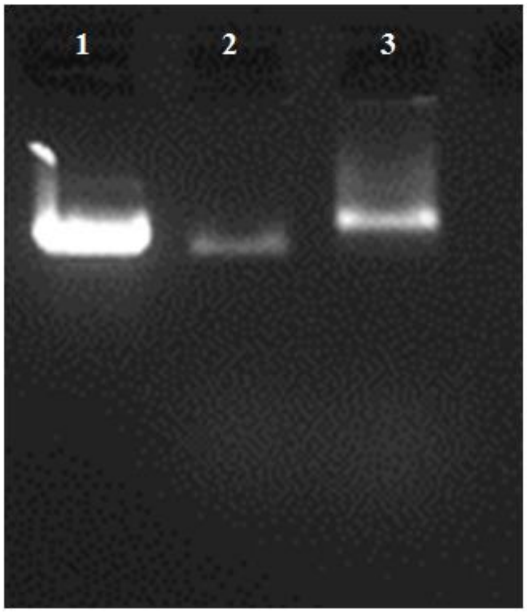

Supplementary Figure 4: Agarose gel electrophoresis (AGE) to characterize the full-cage structure (lane 1: M13 DNA, lane 2: half-cage; lane 3: full-cage). According to the gel band intensity, the assembly yield of the full-cage was higher than 90%.

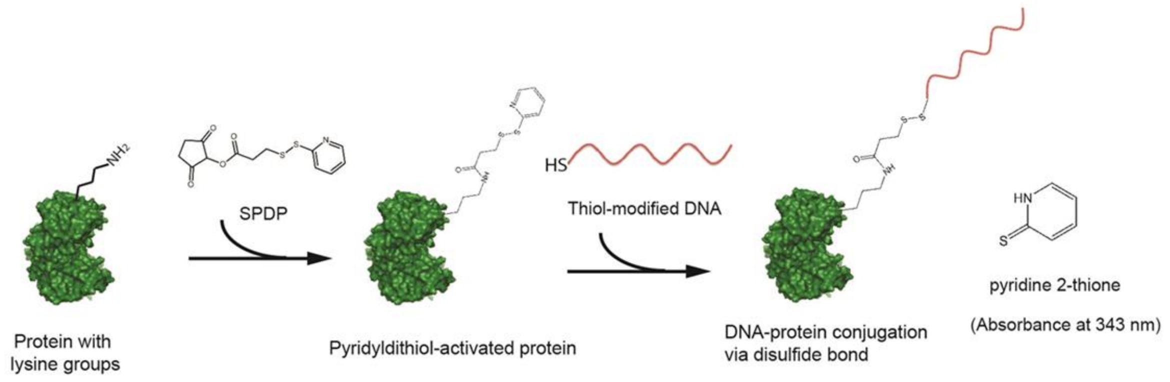

Supplementary Figure 5: Schematic illustration of the SPDP conjugation chemistry used for protein―DNA conjugation.

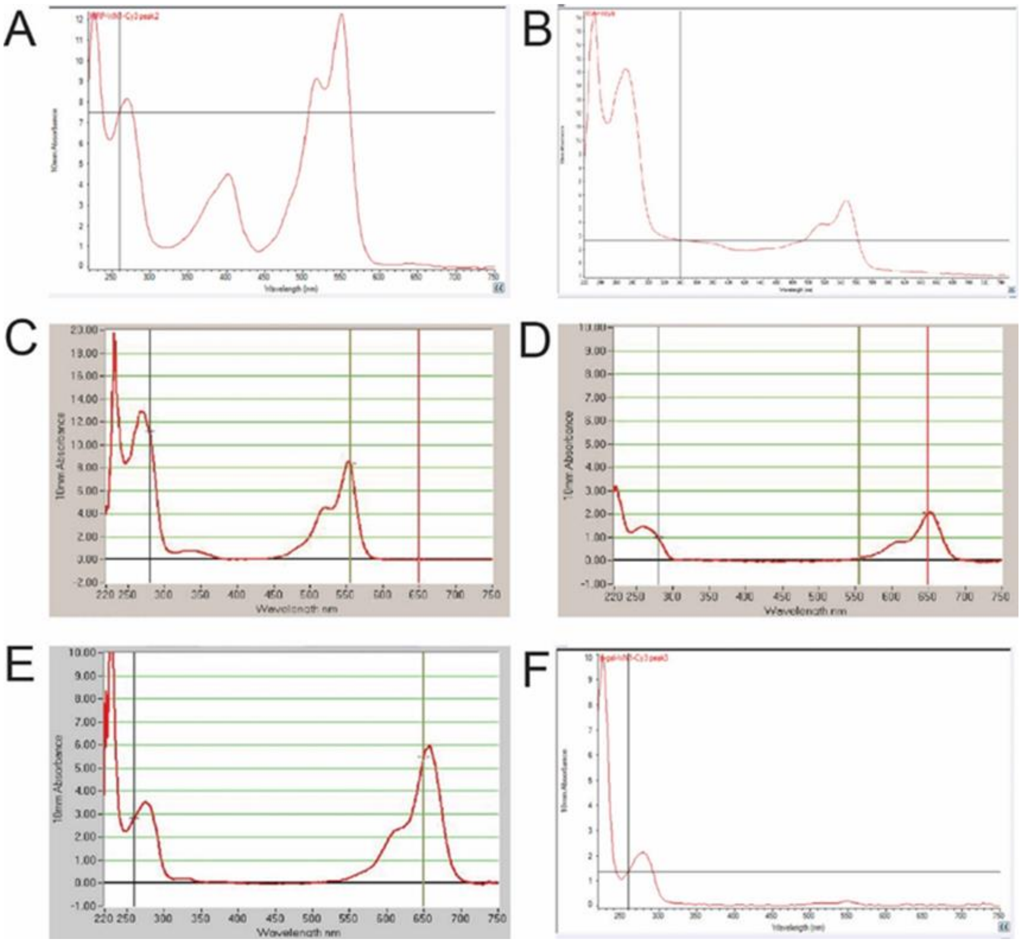

Supplementary Figure 6: Quantification of fluorescent dye-labeled enzyme-DNA conjugates using UV-Vis absorbance spectroscopy. (A) Cy3-labeled HRP-TTTTTCCCTCCCTCC with an average dye-to-protein ratio of ~1.8; (B) Cy3-labeled GOx-TTTTTCCCTCCCTCC with an average dye-to-protein ratio of ~1.5; (C) Cy3-labeled G6pDH-TTTTTCCCTCCCTCC with an average dye-to-protein ratio of ~1.6; (D) Alexa Fluor 647-labeled MDH-TTTTTGGCTGGCTGG with an average dye-to-protein ratio of ~1.2; (E) Alexa Fluor 647-labeled LDH-TTTTTGGCTGGCTGG with an average dye-to-protein ratio of ~1.7; (F) Cy3-labeled (β-Gal)-TTTTTCCCTCCCTCC with an average dye-to-protein ratio of ~0.6.

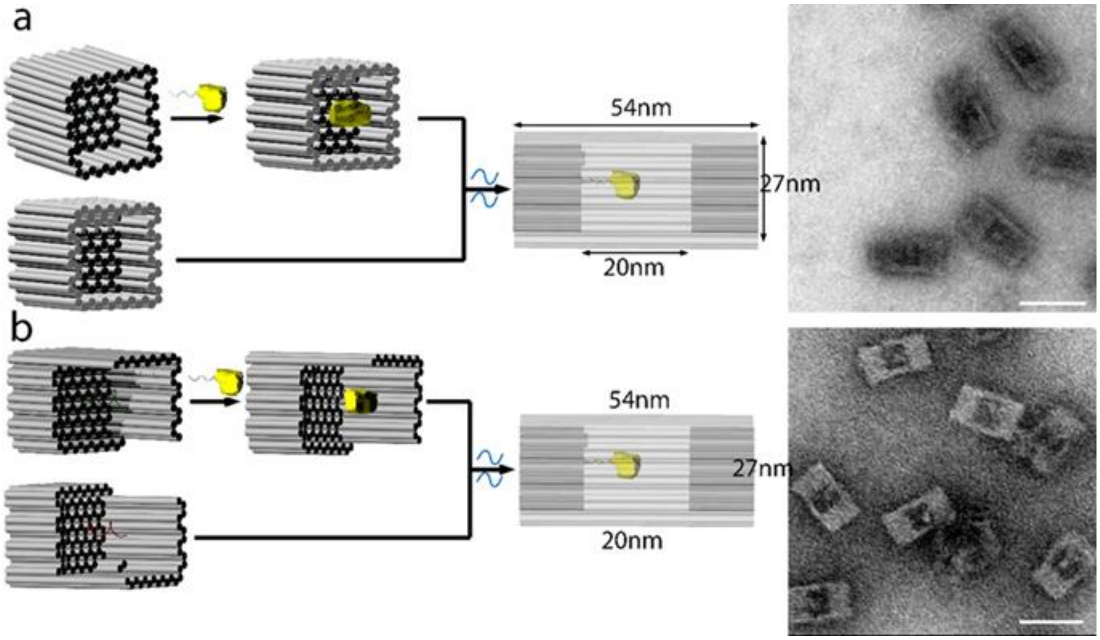

Supplementary Figure 7: Two different designs for the cage structure with different encapsulation yields (see Supplementary Figures 8 and9), assembled with GOx. (a) Cage with closed-wall design. (b) Cage with open-wall design.

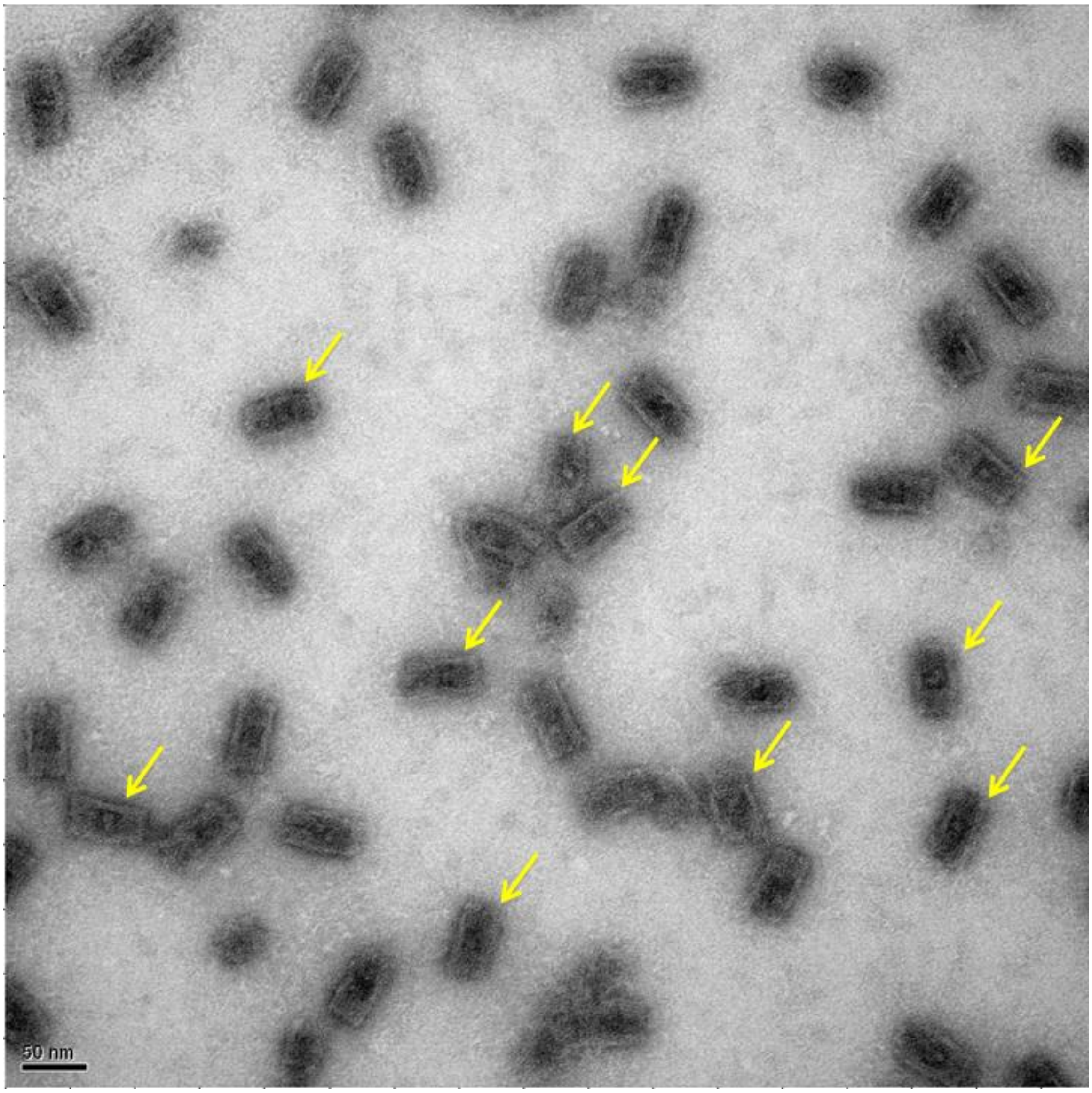

Supplementary Figure 8: TEM image of full-cages with closed-wall design (Supplementary Figure 7a) encapsulating GOx. An encapsulation yield of 38% was estimated from similar images containing ~230 DNA cages by dividing the number of cages with a discernible protein inside by the total number of the cages counted (yellow arrow indicates DNA cage with enzyme inside).

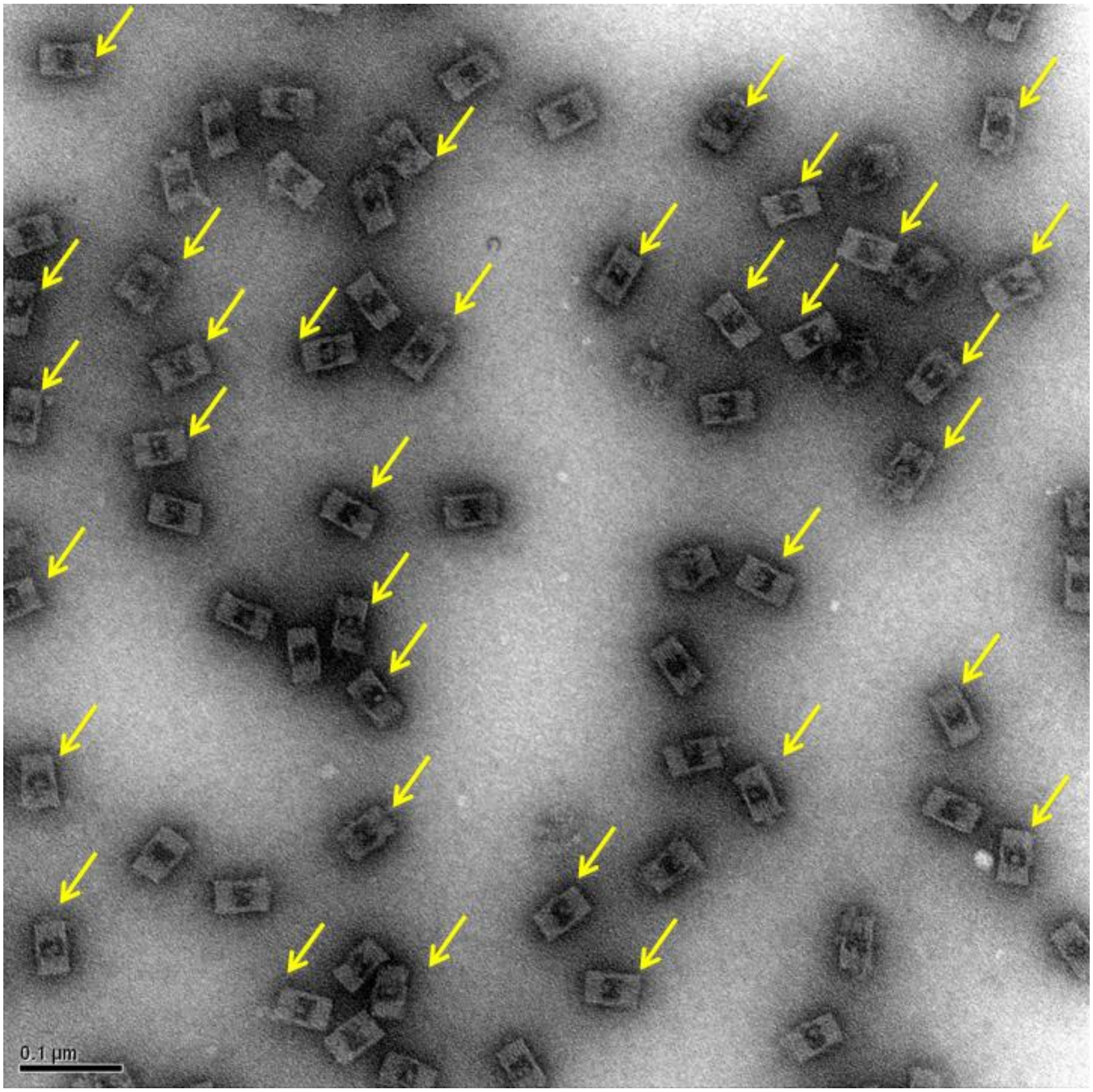

Supplementary Figure 9: TEM image of full-cage with open-wall design (Supplementary Figure. 7b) encapsulating GOx. An encapsulation yield of 77% was estimated from similar images containing ~300 DNA cages by dividing the number of cages with a discernable protein inside by the total number of cages counted (yellow arrow indicates DNA cage with enzyme inside).

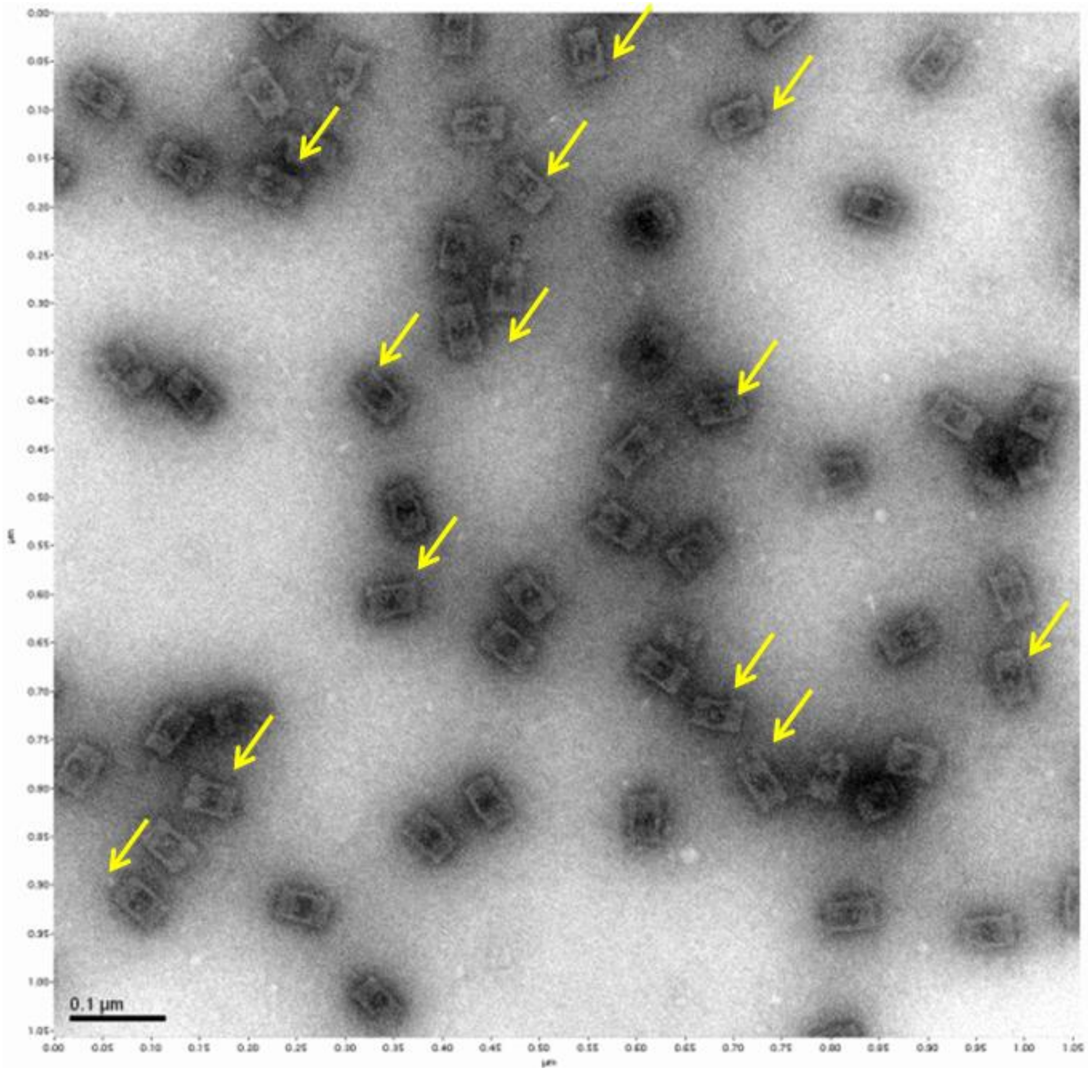

Supplementary Figure 10: TEM image for HRP-GOx enzyme pairs encapsulated in DNA full-cage. Despite variable quality of staining across the field of view, the inner cavity of many nanocages appeared to contain two bright spots, which we interpreted as intact HRP-GOx enzyme pairs (yellow arrow indicates DNA cage with enzyme pair inside).

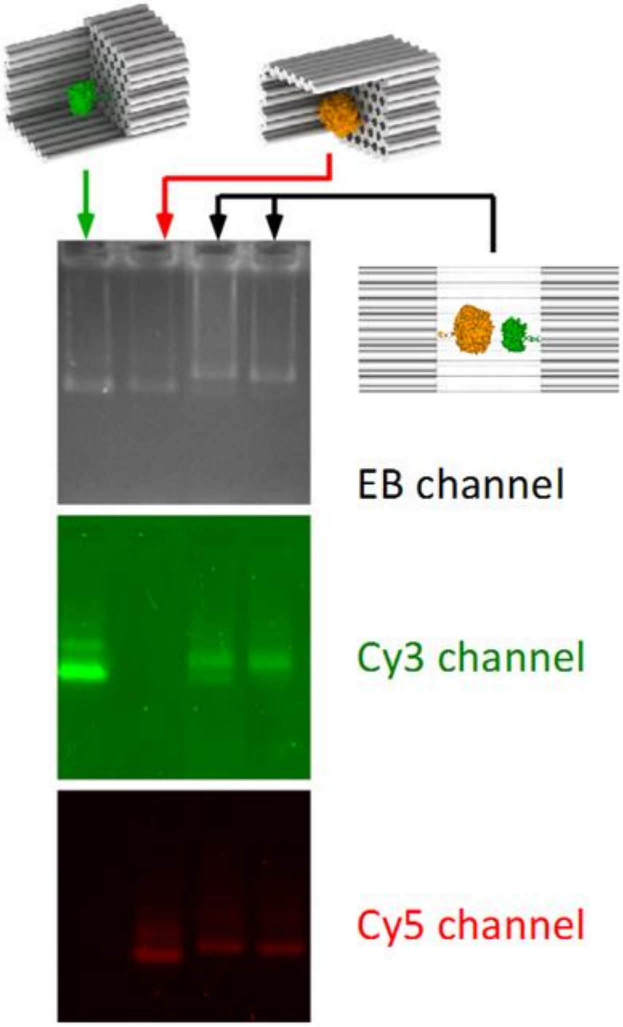

Supplementary Figure 11: Native AGE characterization of a DNA nanocage encapsulating a GOx/HRP pair. GOx and HRP were conjugated with Cy3 and Cy5, respectively. Lane 1 (from left): half-cage assembled with GOx-Cy3, lane 2: half-cage assembled with HRP-Cy5, lanes 3 and 4: full-cage with GOx/HRP. "EB" indicates ethidium bromide staining of the gel to visualize all DNA bands.

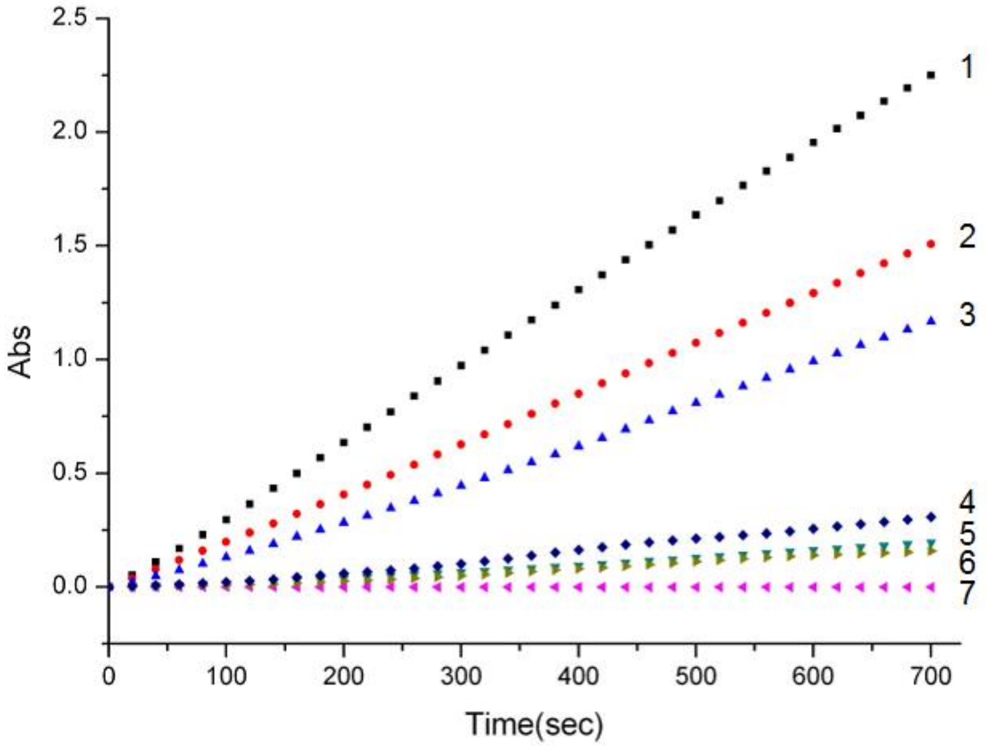

Supplementary Figure 12: Raw activity for a set of DNA cage-encapsulated enzymes. 1: Full[H+G], a full cage-encapsulated GOx and HRP; 2: Full[H] + Full[G], a full cage-encapsulated HRP and a full cage-encapsulated GOx; 3: half[H] + half[G], a half cage-encapsulated HRP and a half-cage encapsulated GOx; 4: Full + H + G, a full cage incubated with a pair of free HRP and GOx; 5: H + G fresh control, a fresh solution of free HRP and GOx; 6: H + G annealing control, a solution of free HRP and GOx that is incubated using the same thermal program as the DNA cage-encapsulated enzymes; 7: substrate background control. Assay conditions: 1 nM enzyme or enzyme-encapsulating DNA cage, with 1 mM Glucose, 2 mM ABTS in pH 7.5, 1×TBS buffer. Absorbance is monitored at 410 nm.

Determination of the Michaelis-Menten constants for enzymes-HRP

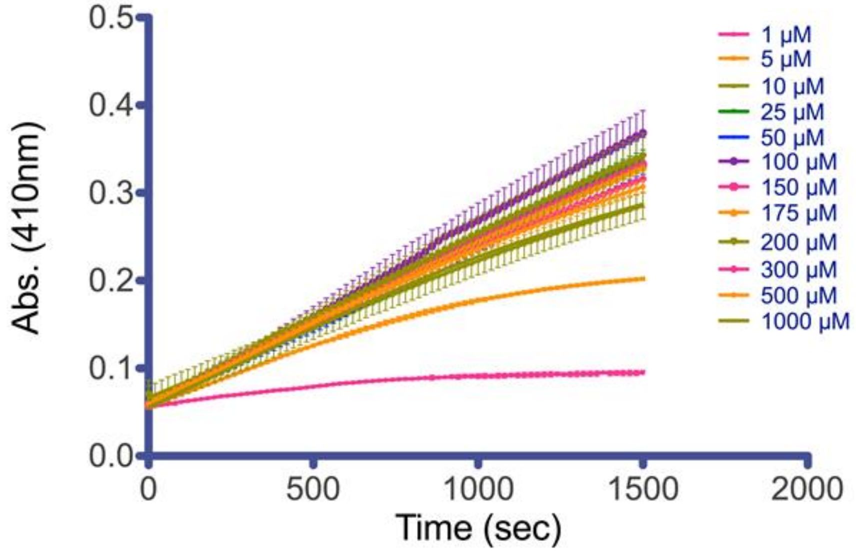

Supplementary Figure 13: Raw activity for free enzyme solution of DNA-conjugated HRP (0.5 nM) with H_2O_2 concentration varied from 1 μM to 1000 μM, and 2 mM ABTS, monitoring absorbance at 410 nm. Error bars were calculated from the standard deviation of at least three replicates.

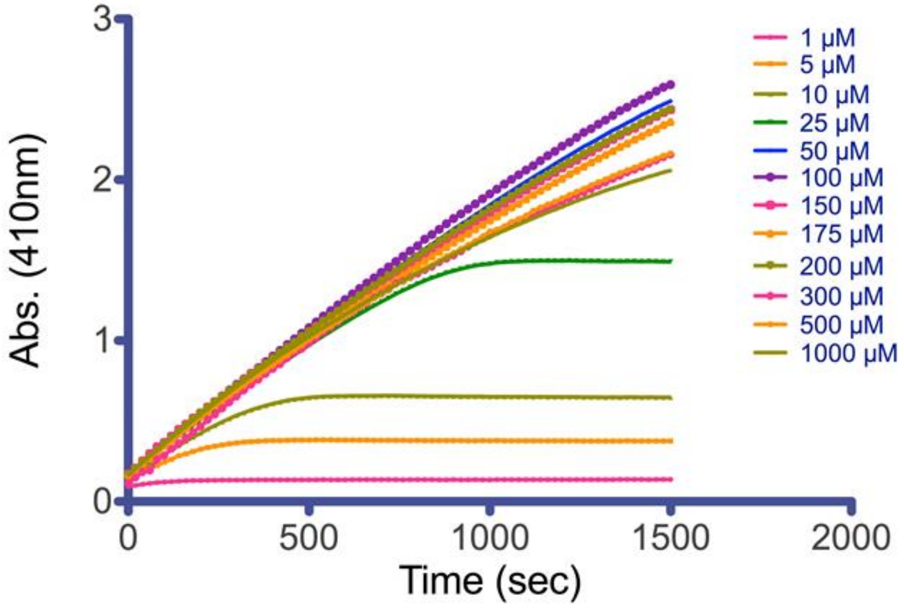

Supplementary Figure 14: Raw activity for DNA cage-encapsulating HRP (0.5 nM) with H_2O_2 varied from 1 µM to 1000 µM and 2 mM ABTS, monitoring absorbance at 410 nm. Error bars were calculated from the standard deviation of at least three replicates.

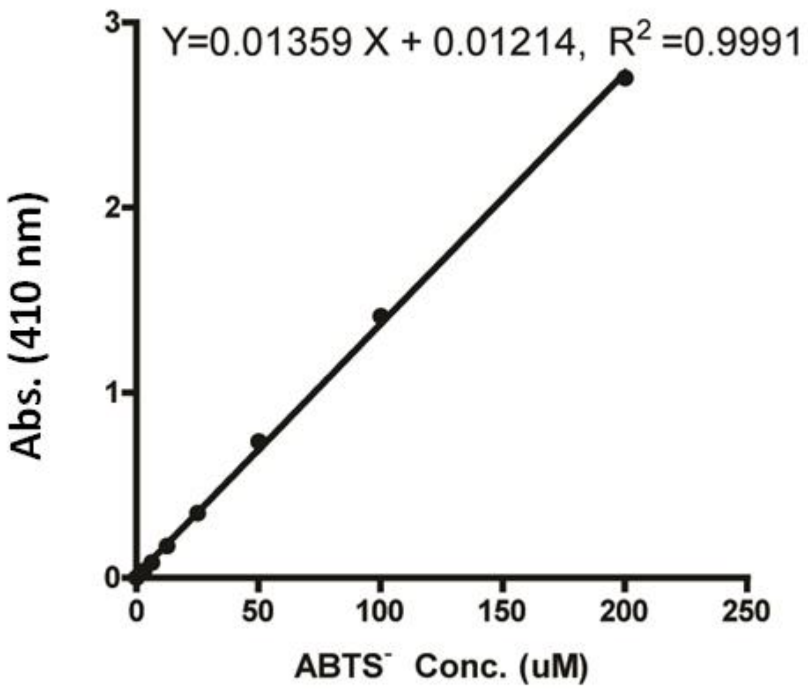

Supplementary Figure 15: ABTS standard curve to calculate k_{cat} value (Y=0.01359X+0.01214).

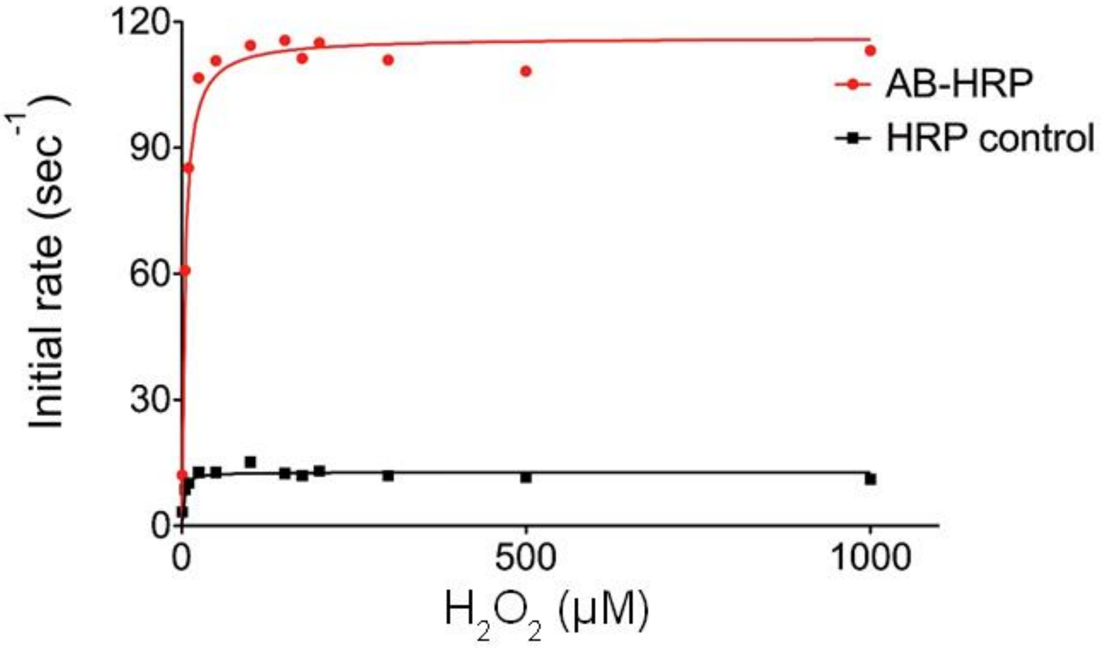

	K_M (μM)	k_{cat} (s^{-1})
**Full Cage[HRP]**	4.3±0.6	290±5
**Free HRP**	2.3±0.5	32±1

Supplementary Figure 16: Michaelis-Menten plot of HRP encapsulated within a full-cage (Full-Cage[HRP], red circles), compared with that of free HRP (HRP control, black squares) using H_2O_2 as the substrate. The solid lines represent fits of the Michaelis-Menten model to the data. Enzyme assay conditions: 0.5 nM enzyme or DNA-cage-encapsulated enzyme, 2 mM ABTS with different concentrations of H_2O_2 ranging from 1 μM to 1000 μM, in 1×TBS buffer (pH 7.5, 1 mM $MgCl_2$), absorbance monitored at 410 nm. The table lists the fit parameters. Full-cage encapsulation of the enzyme caused a ~2-fold increase in K_M and a ~9-fold increase in k_{cat}.

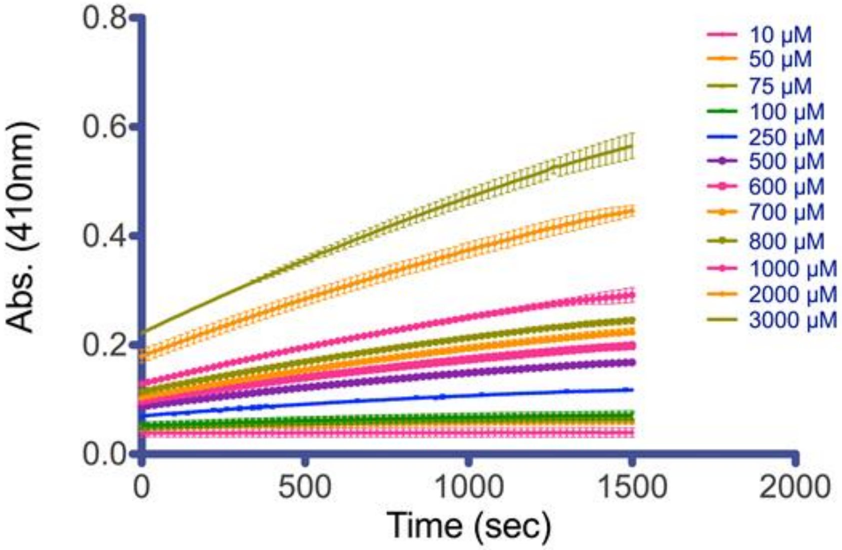

Supplementary Figure 17: Raw activity measurement of Full-Cage [HRP] (0.5 nM) with ABTS concentration varied from 10 μM to 3000 μM and 2000 μM H_2O_2, monitoring absorbance at 410 nm. Error bars were calculated from the standard deviation of at least three replicates.

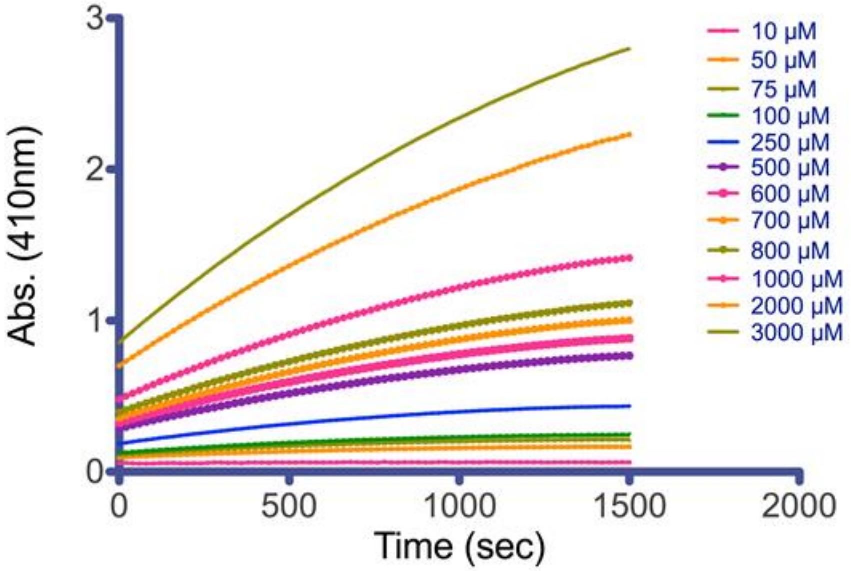

Supplementary Figure 18: Raw activity measurement of free DNA-conjugated HRP (0.5 nM) with ABTS concentration varied from 10 μM to 3000 μM, and 2000 μM H_2O_2, monitoring absorbance at 410 nm. Error bars were calculated from the standard deviation of at least three replicates.

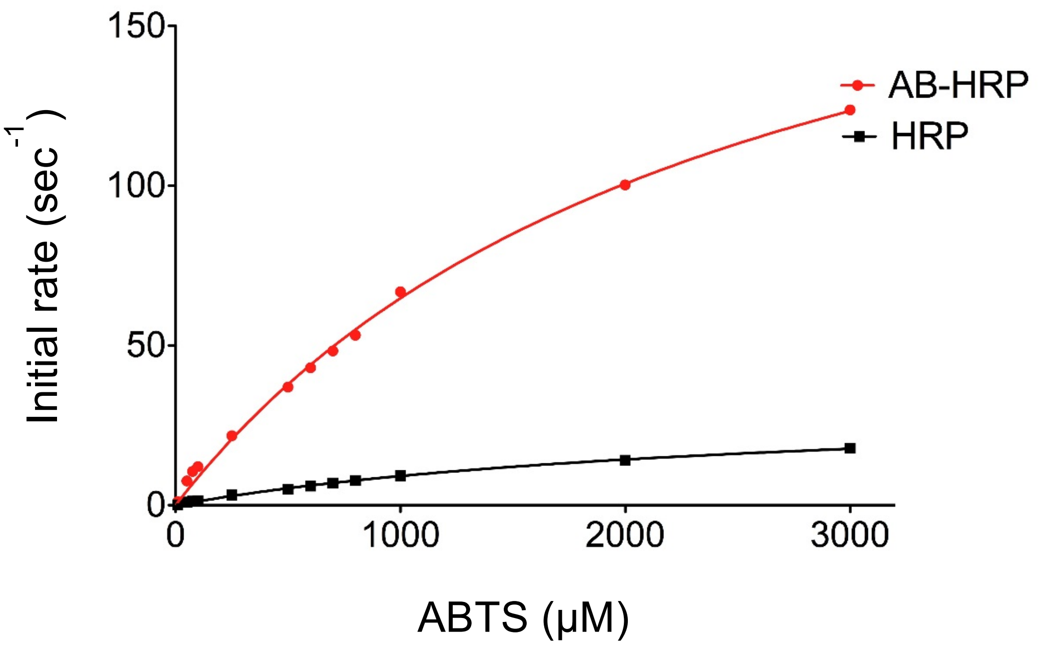

	K_M (µM)	k_{cat} (s^{-1})
**AB-HRP**	2500±200	560±20
**HRP control**	2600±400	59±5

Supplementary Figure 19: Michaelis-Menten plot for HRP encapsulated within a full-cage (AB-HRP, red circles), compared with that of free HRP enzyme (HRP control, black squares) using ABTS as the substrate. The solid lines represent fits of the Michaelis-Menten model to the data. Enzyme assay conditions: 0.5 nM enzyme or full-cage-encapsulated enzyme, 2000 µM H_2O_2 with different concentrations of ABTS, ranging from 10 µM to 3000 µM, in 1×TBS buffer (pH 7.5, 1 mM $MgCl_2$), monitoring absorbance at 410 nm. The table lists the fit parameters. DNA encapsulation of the enzyme caused no change in K_M and a ~9-fold increase in k_{cat}.

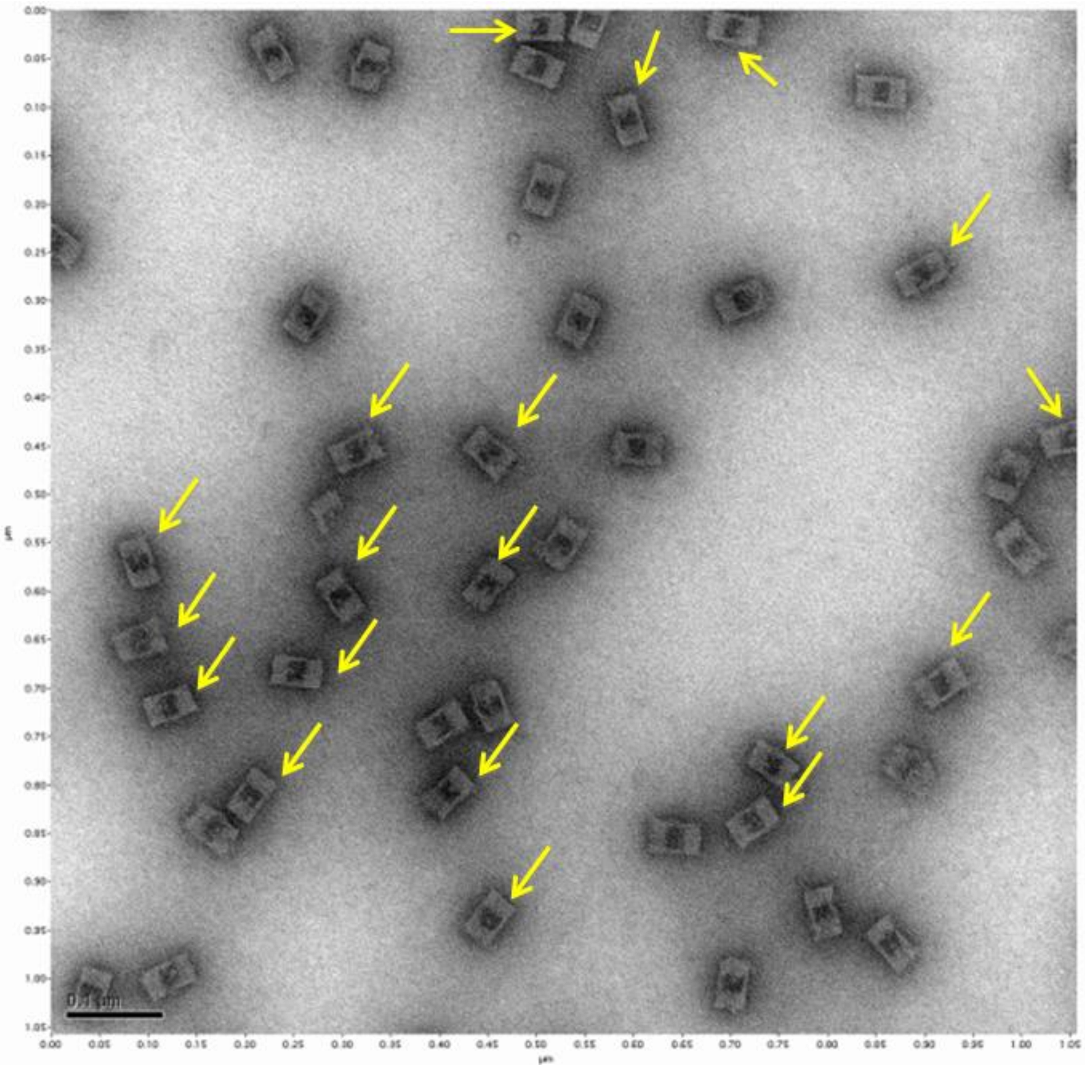

Supplementary Figure 20: TEM image for the purified DNA full-cage with only HRP enzyme inside. Scale bar: 100 nm. The majority of cages showed one lighter spot inside the cavity, representing the enzyme. Despite variable quality of staining across the field of view, the inner cavity of many nanocages appeared to contain one bright spot, which we interpreted as intact one HRP enzyme (yellow arrow indicates DNA cage with enzyme inside).

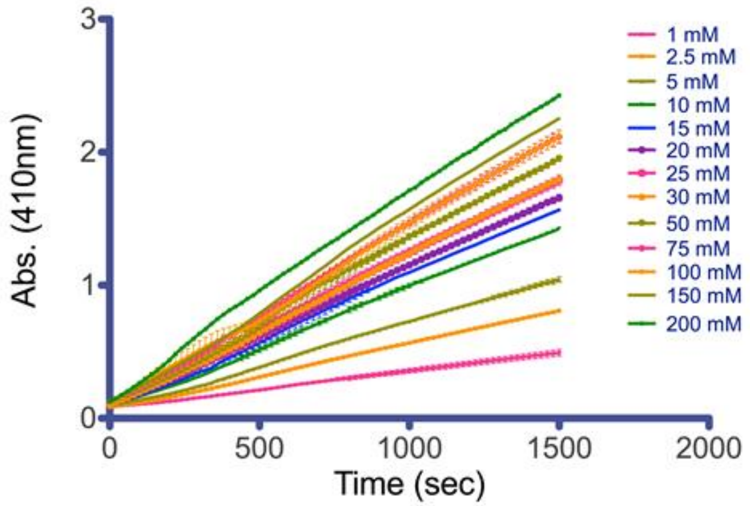

Supplementary Figure 21: Raw activity for free DNA-conjugated GOx (0.5 nM) with different concentrations of glucose ranging from 1 mM to 200 mM. 2 mM ABTS and 100 nM HRP were used to quickly convert H_2O_2 to detectable signal that was monitored at 410 nm. Error bars were calculated from the standard deviation of at least three replicates.

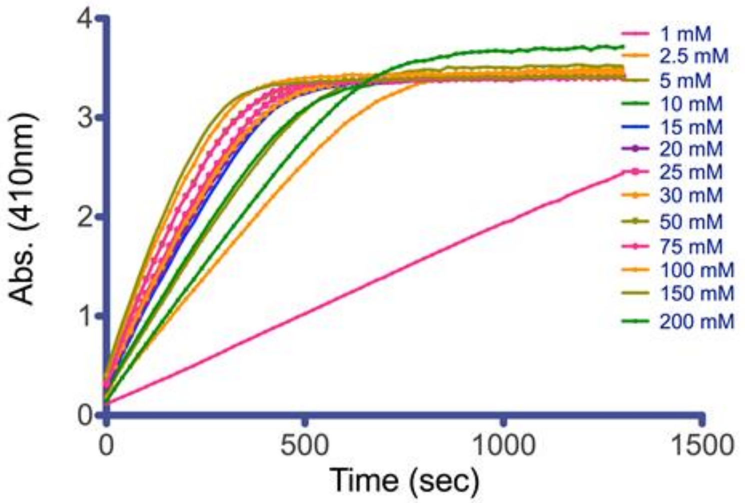

Supplementary Figure 22: Raw activity for DNA cage-encapsulating GOx (0.5 nM) with different concentrations of glucose ranging from 1 mM to 200 mM. 2 mM ABTS and 100 nM HRP were used to quickly convert H_2O_2 to detectable signal that was monitored at 410 nm. Error bars were calculated from the standard deviation of at least three replicates.

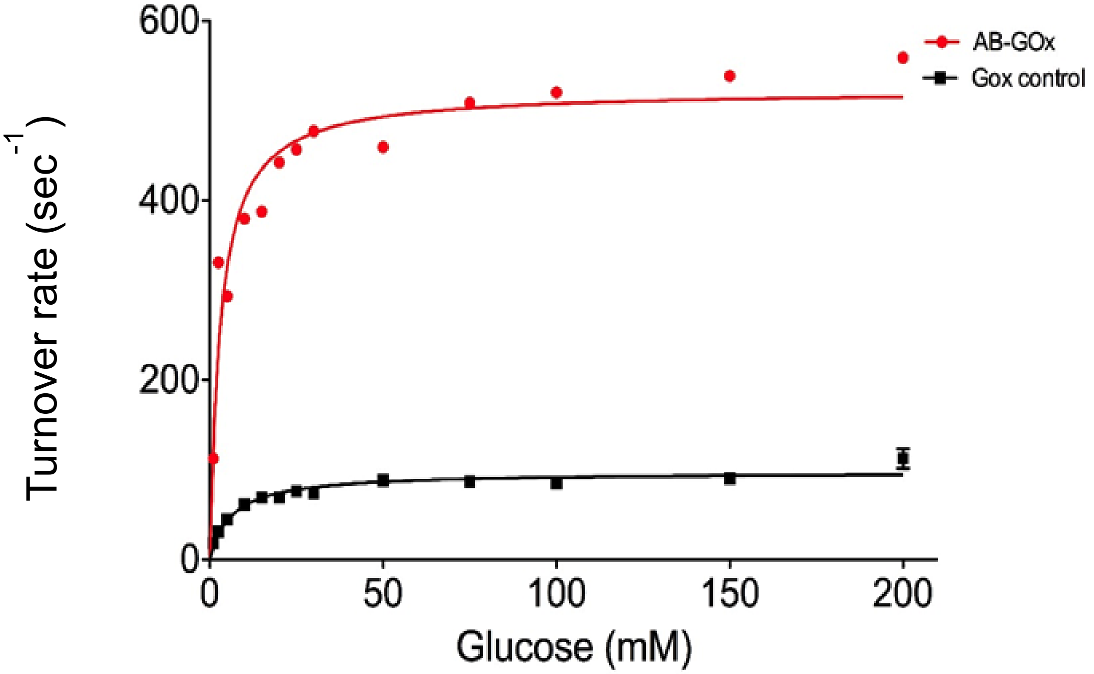

	K_M (μM)	K_{cat} (s^{-1})
**AB-GOx**	3000±600	1300±50
**GOx control**	6200±900	240±10

Supplementary Figure 23: Michaelis-Menten plot of GOx inside the full-cage (AB-GOx, red circles), compared with that of free GOx enzyme (GOx control, black squares) using glucose as the substrate. The solid lines represent fits of the Michaelis-Menten model to the data. Enzyme assay conditions: 0.5 nM enzyme or DNA cage encapsulated enzyme, 2 mM ABTS, 100 nM HRP with different concentrations of glucose ranging from 1 mM to 200 mM, in 1×TBS buffer (pH 7.5, 1 mM $MgCl_2$) monitoring absorbance at 410 nm. The table lists the fit parameters. DNA encapsulation of the enzyme caused a ~2-fold decrease in K_M and a ~5-fold increase in k_{cat}.

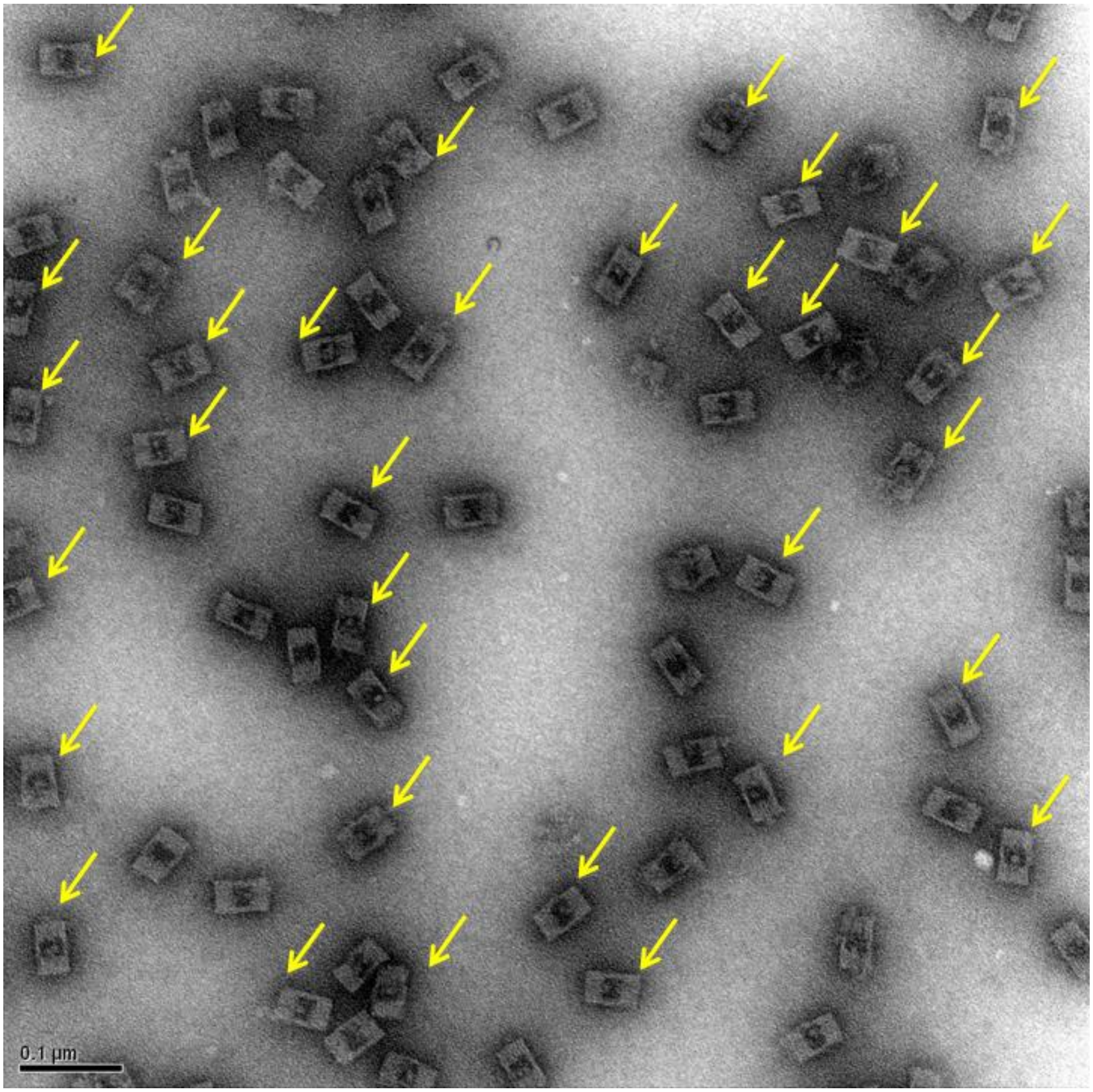

Supplementary Figure 24: TEM image of the purified DNA full-cage with only GOx inside (yellow arrow indicates DNA cage with enzyme inside).

Determination of the Michaelis-Menten constants for enzymes-G6PDH

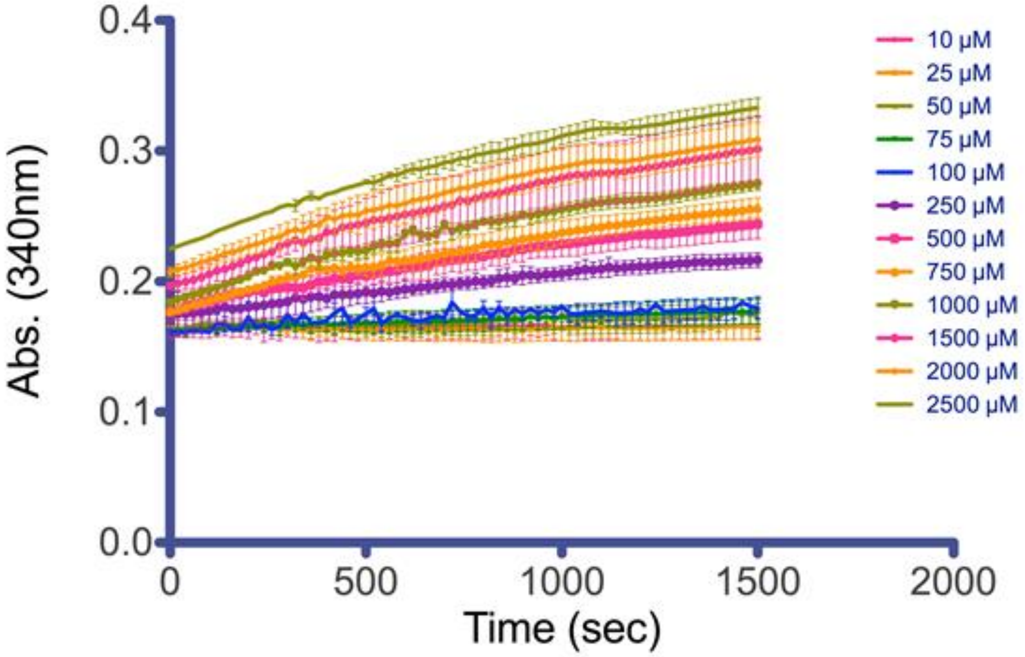

Supplementary Figure 25: Raw activity for free DNA-modified G6pDH (0.5 nM) with 10-2500 μM NAD^+ and 1 mM glucose 6-phosphate, monitoring absorbance at 340 nm. Error bars were calculated from the standard deviation of at least three replicates.

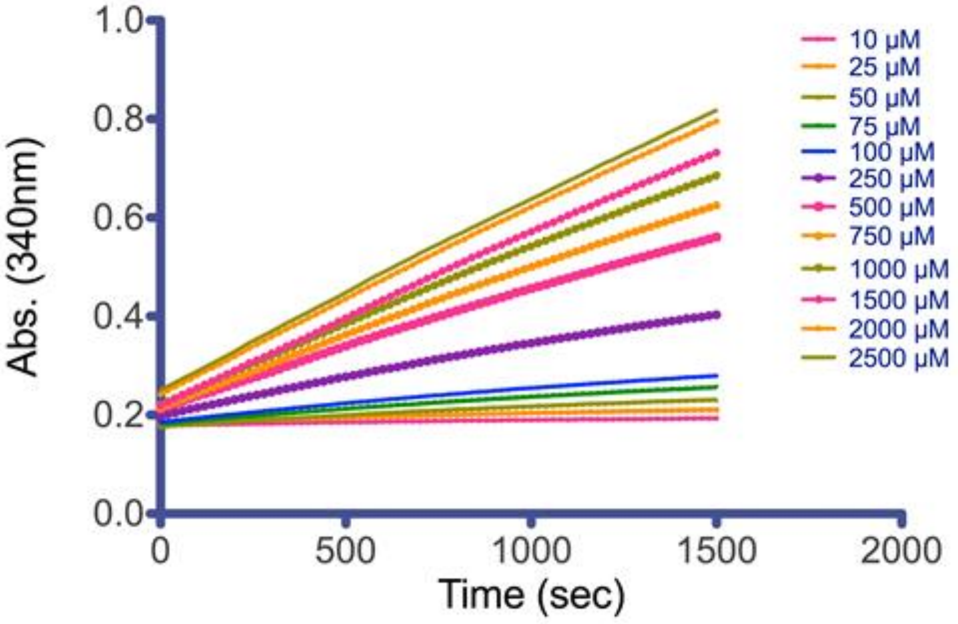

Supplementary Figure 26: Raw activity for Full-Cage [G6pDH] (0.5 nM) with 10-2500 µM NAD^+ and 1 mM glucose 6-phosphate, monitoring absorbance at 340 nm. Error bars were calculated from the standard deviation of at least three replicates.

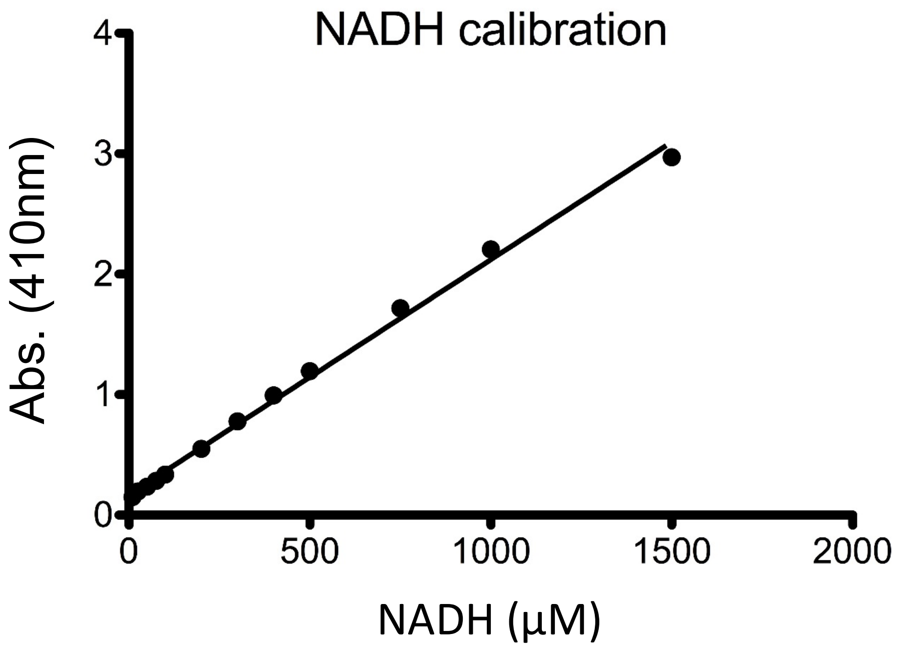

Supplementary Figure 27: NADH absorbance standard curve to calculate k_{cat} (Y=0.001951X+0.1694).

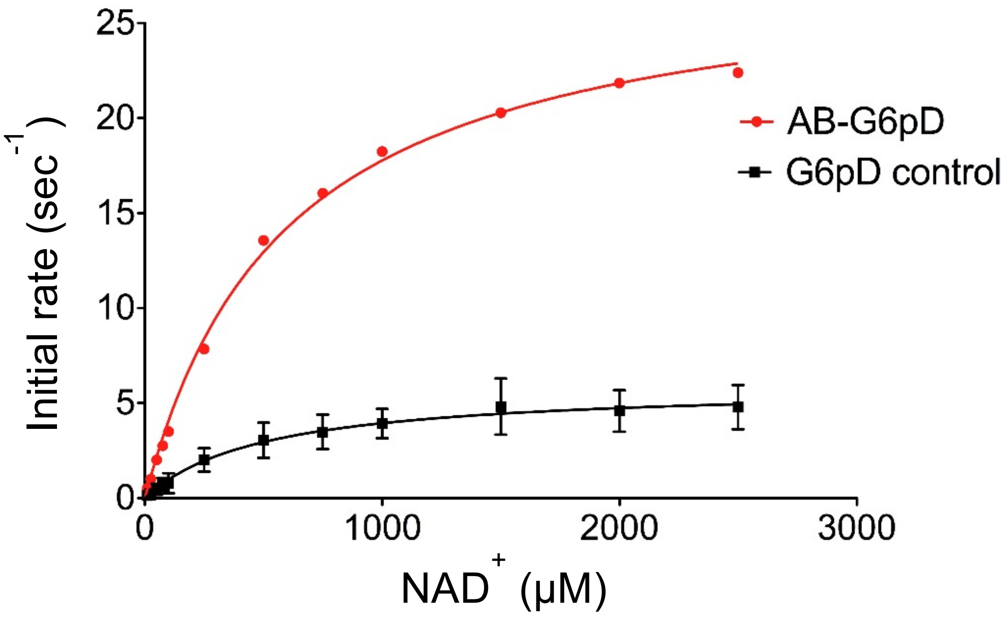

	K_M (μM)	k_{cat} (s^{-1})
**Full[G6pDH]**	590±40	480±10
**G6pDH control**	510±50	100±3

Supplementary Figure 28: Michaelis-Menten plot of Full-Cage[G6PDH] (red circles) compared with that of free G6pDH (black square), using NAD^+ as the varying substrate. The solid lines represent fits of the Michaelis-Menten model to the data. Enzyme assay conditions: 0.5 nM enzyme or DNA cage-encapsulated enzyme, 1 mM glucose 6-phosphate, with different concentrations of NAD^+ ranging from 10 μM to 2500 μM, in 1×TBS buffer (pH 7.5, 1 mM $MgCl_2$), monitoring absorbance at 340 nm. The table lists the fit parameters. Full-cage encapsulation of the enzyme caused little change in K_M and a ~5-fold increase in k_{cat}. Error bars were calculated from the standard deviation of at least three replicates.

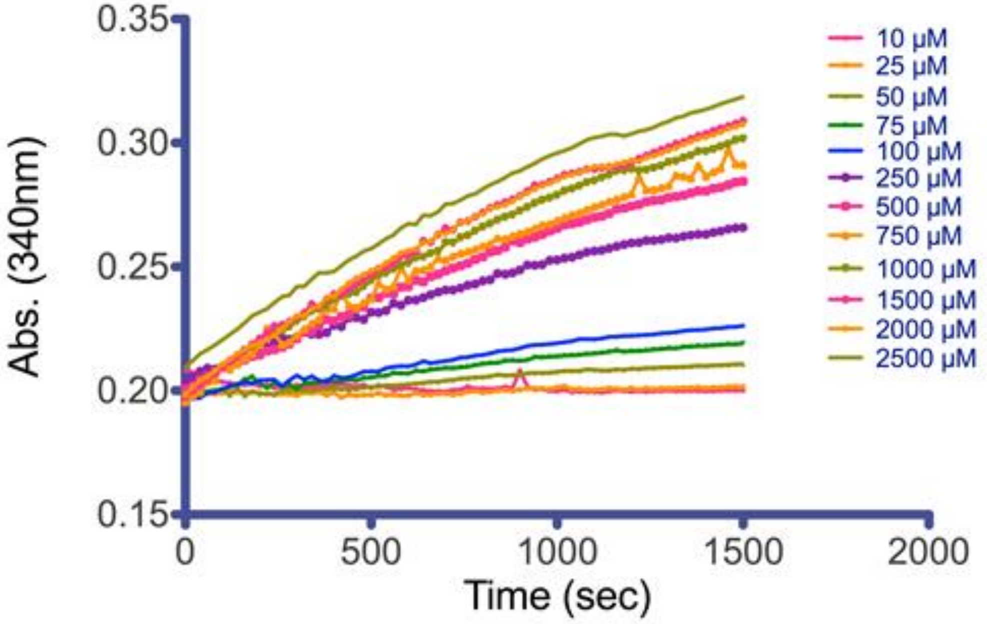

Supplementary Figure 29: Raw activity for free DNA-modified G6pDH (0.5 nM) with glucose 6-phosphate varied from 10 µM to 2500 µM, and 1 mM NAD^+, monitoring at 340 nm. Error bars were calculated from the standard deviation of at least three replicates.

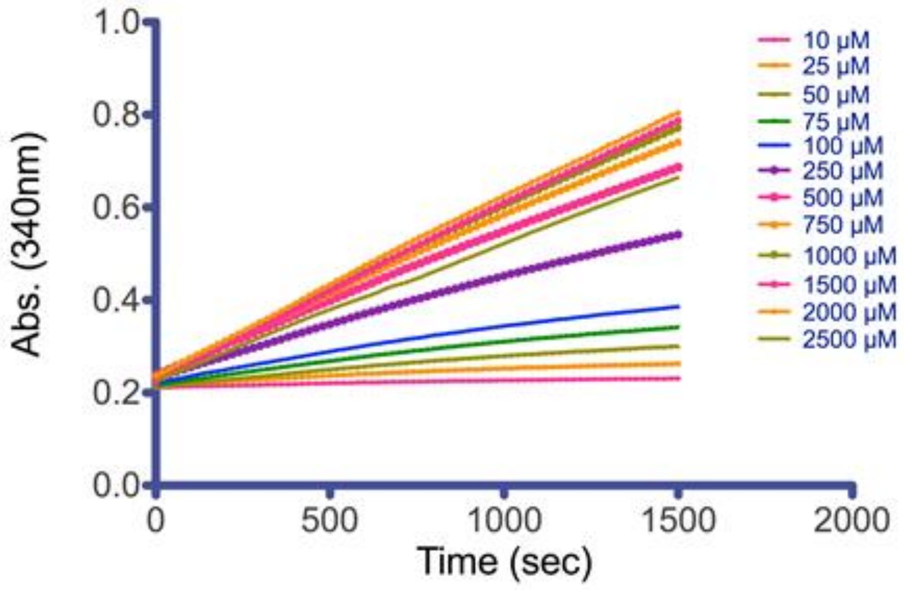

Supplementary Figure 30: Raw activity for Full-Cage [G6pDH] (0.5 nM) with glucose 6-phosphate varied from 10 µM to 2500 µM, and 1 mM NAD^+, monitoring absorbance at 340 nm. Error bars were calculated from the standard deviation of at least three replicates.

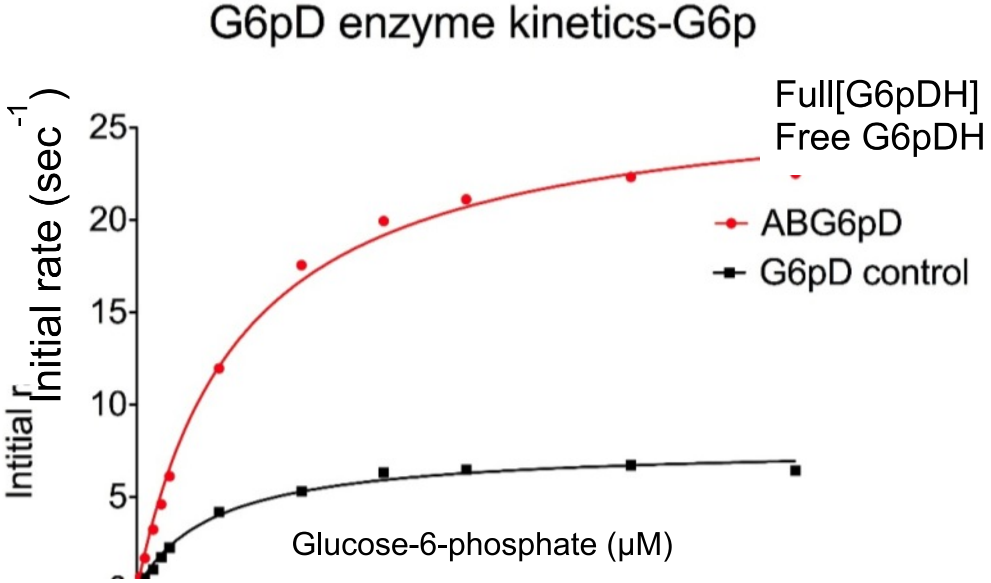

	K_M (μM)	k_{cat} (s^{-1})
**Full[G6pDH]**	310±30	460±10
**G6pDH control**	220±20	130±3

Supplementary Figure 31: Michaelis-Menten plot of Full-Cage [G6pDH] (red circles), compared with that of the free G6pDH enzyme (black squares), using glucose 6-phosphate as the substrate. The solid lines represent fits of the Michaelis-Menten model to the data. Enzyme assay conditions: 0.5 nM enzyme or DNA cage-encapsulated enzyme, 1mM NAD^+, with different concentration of glucose-6-phosphate ranging from 10 μM to 2000 μM, in 1×TBS buffer (pH 7.5, 1 mM $MgCl_2$) monitoring absorbance at 340 nm. The table lists the fitting parameters. DNA encapsulation of the enzyme caused a ~1.4-fold increase in K_M and a ~4-fold increase in k_{cat}.

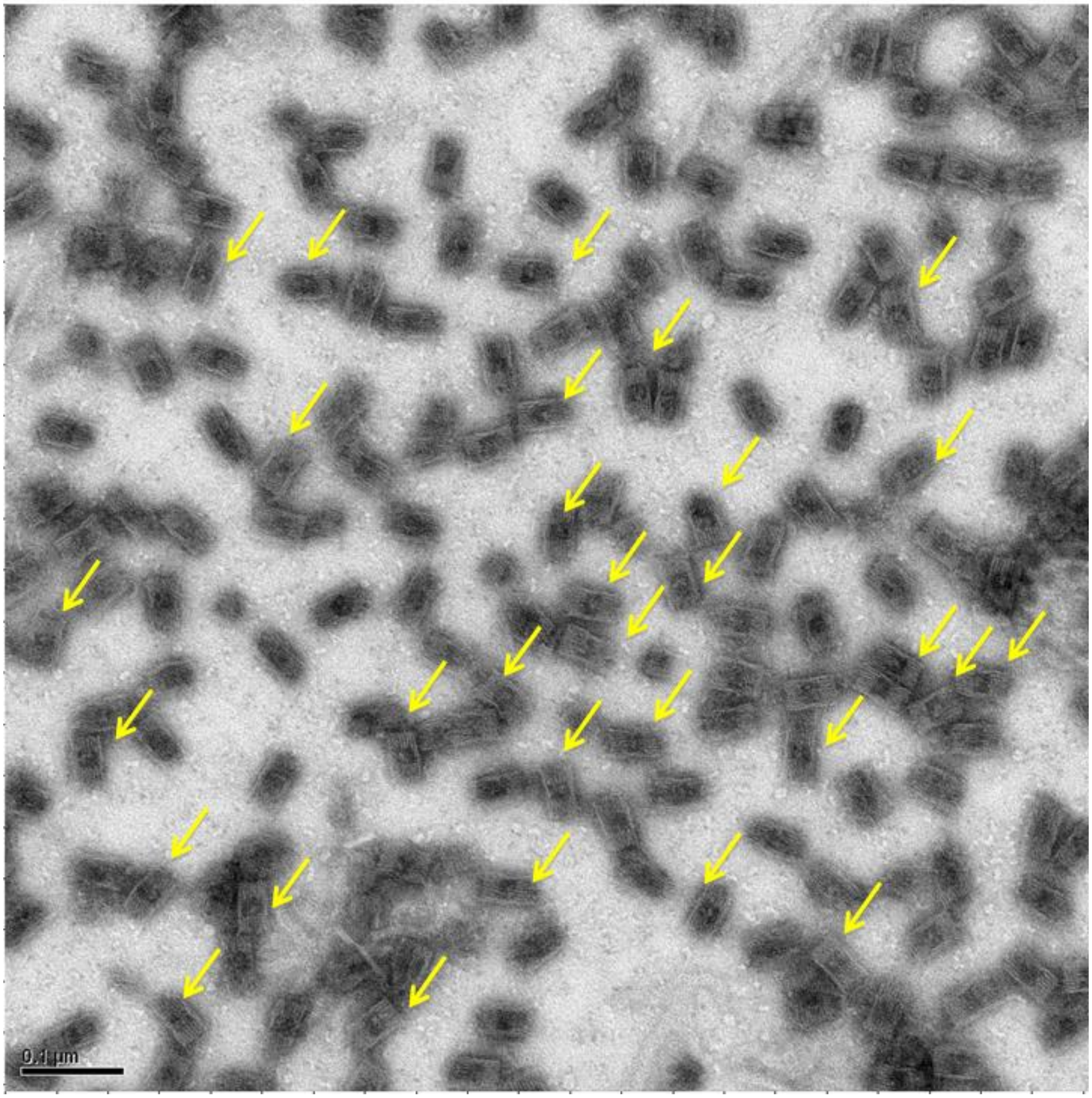

Supplementary Figure 32: TEM image of AGE-purified DNA full-cages with G6pDH inside (yellow arrow indicates DNA cage with enzyme inside).

Determination of the Michaelis-Menten constants for enzymes-MDH

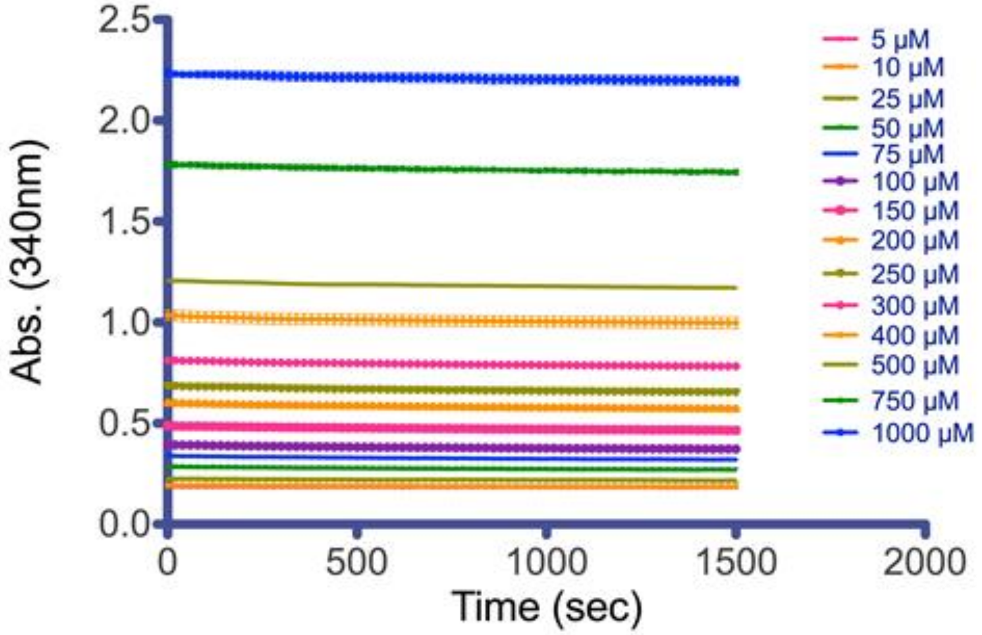

Supplementary Figure 33: Raw activity for free DNA-modified MDH (0.5 nM) with 5-1000 µM NADH and 2 mM OAA, monitoring absorbance at 340 nm. Error bars were calculated from the standard deviation of at least three replicates.

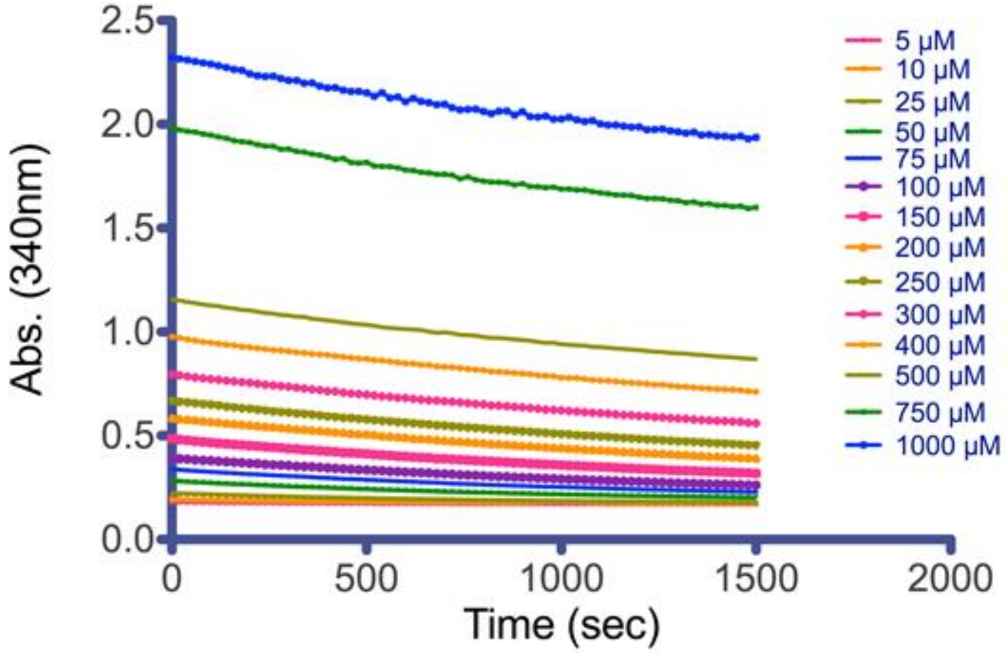

Supplementary Figure 34: Raw activity for Full-Cage [MDH] (0.5 nM) with 5-1000 μM NADH and 2 mM OAA, monitoring absorbance at 340 nm. Error bars were calculated from the standard deviation of at least three replicates.

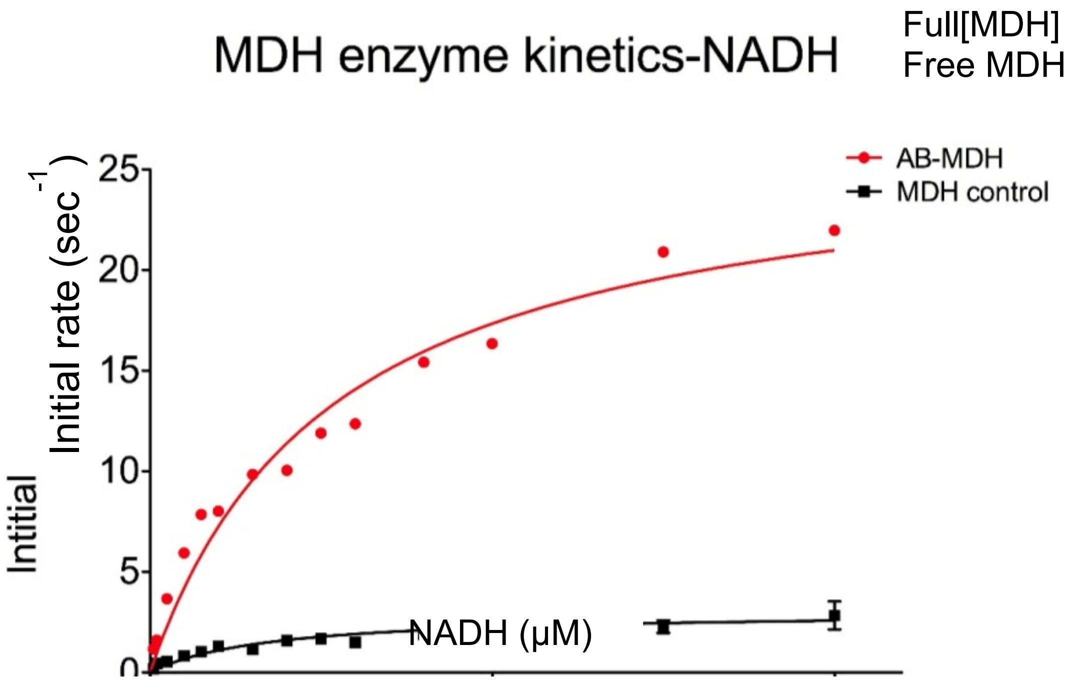

	K_M (µM)	k_{cat} (s^{-1})
**Full[MDH]**	270±50	460±30
**MDH control**	180±50	51±5

Supplementary Figure 35: Michaelis-Menten plot of Full-Cage [MDH] (red circles), compared with that of free MDH (black squares) using NADH as the varying substrate. The solid lines represent fits of the Michaelis-Menten model to the data. Enzyme assay conditions: 0.5 nM enzyme or DNA cage-encapsulated enzyme, 2 mM OAA, with different concentration of NADH ranging from 5 µM to 1000 µM, in HEPES buffer (pH 7.5, 1 mM $MgCl_2$) monitoring absorbance at 340 nm. The table lists the fit parameters. DNA encapsulation of the enzyme caused a ~1.5-fold increase in K_M and a ~9-fold increase in k_{cat}.

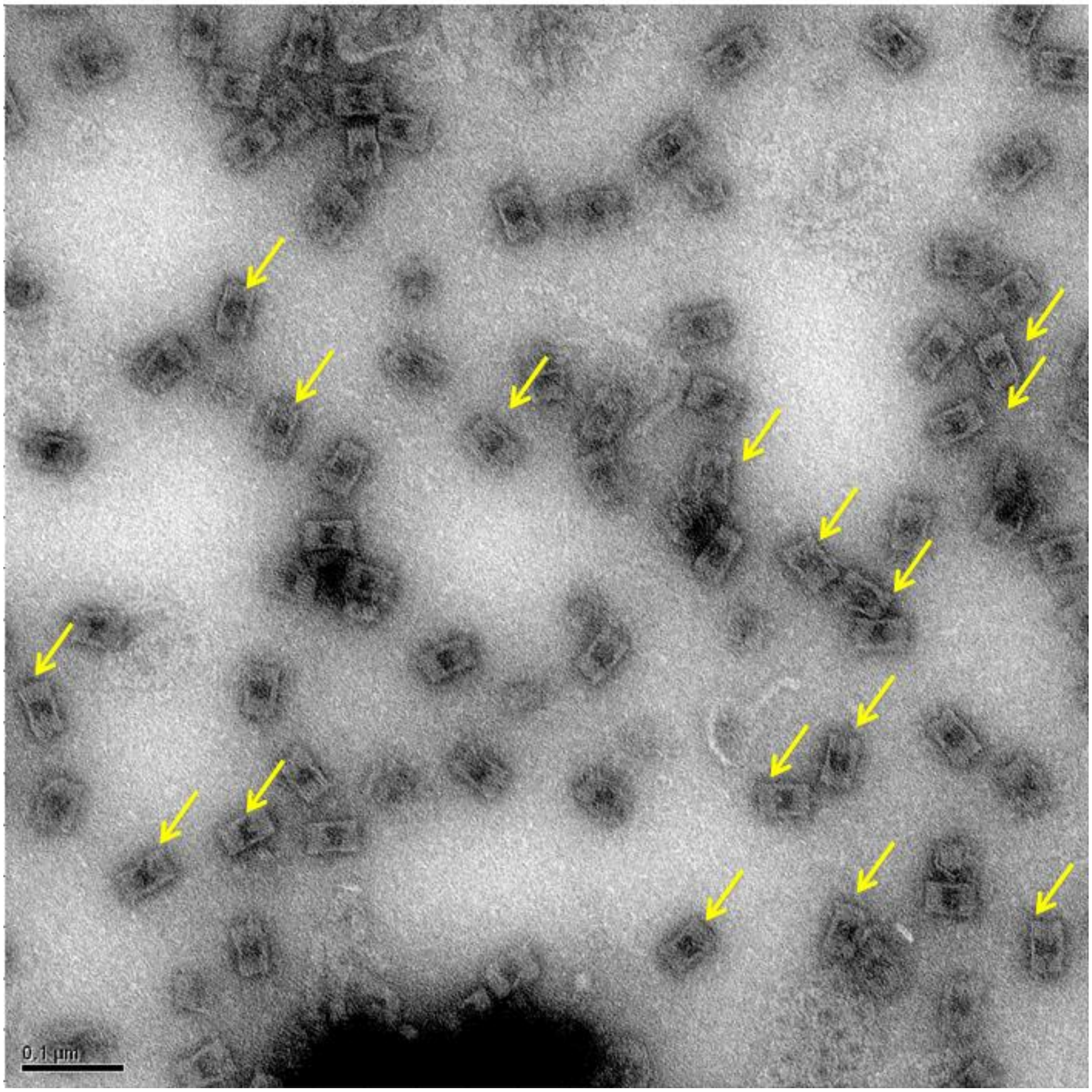

Supplementary Figure 36: TEM image for DNA full-cages with MDH inside (yellow arrow indicates DNA cage with enzyme inside).

Determination of the Michaelis-Menten constants for enzymes-LDH

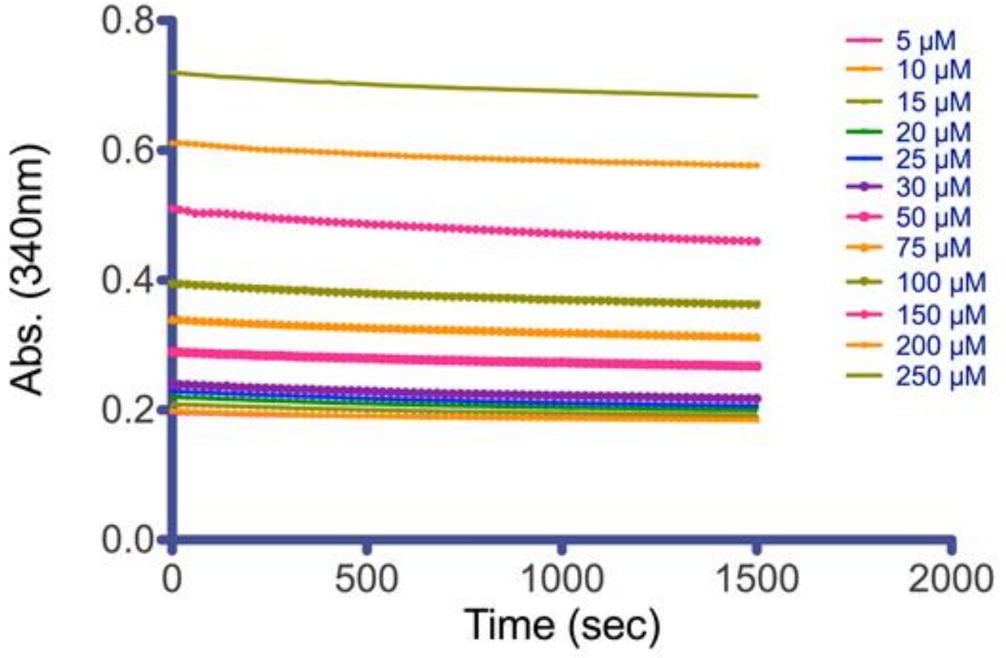

Supplementary Figure 37: Raw activity for free DNA-modified LDH (0.5 nM) with 5-250 µM NADH and 2 mM pyruvate, monitoring absorbance at 340 nm. (Error bars were calculated from the standard deviation of at least three replicates)

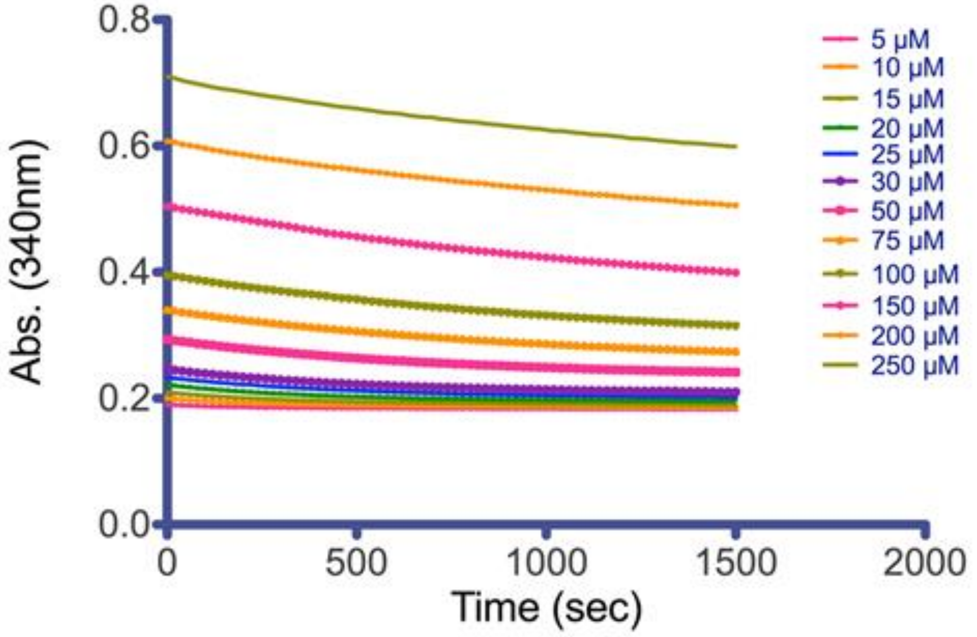

Supplementary Figure 38: Raw activity for full-cage [LDH] (0.5 nM) with 5-250 µM NADH and 2 mM pyruvate, monitoring absorbance at 340 nm. (Error bars were calculated from the standard deviation of at least three replicates)

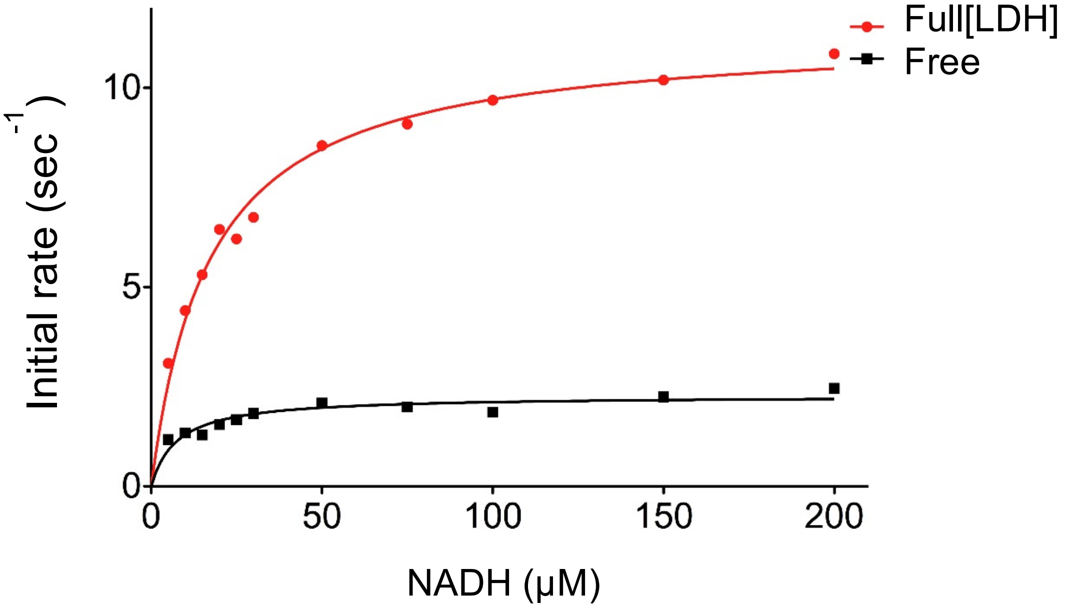

	K_M (µM)	k_{cat} (s^{-1})
**Full[LDH]**	17.0±1.5	190±5
**LDH control**	7.2±1.3	46±2

Supplementary Figure 39: Michaelis-Menten plot of Full-Cage [LDH] (red circles), compared with that of free LDH (black squares), using NADH as the varying substrate. The solid lines represent fits of the Michaelis-Menten model to the data. Enzyme assay condition: 0.5 nM enzyme or DNA cage encapsulated enzyme, 2 mM pyruvate, with different concentration of NADH ranging from 5 µM to 200 µM, in HEPES buffer (pH 7.5, 1 mM $MgCl_2$) monitoring absorbance at 340 nm. The table lists the fit parameters. DNA encapsulation of the enzyme caused a ~2.4-fold increase in K_M and a ~4-fold increase in k_{cat}.

Determination of the Michaelis-Menten constants for enzymes - β-Gal

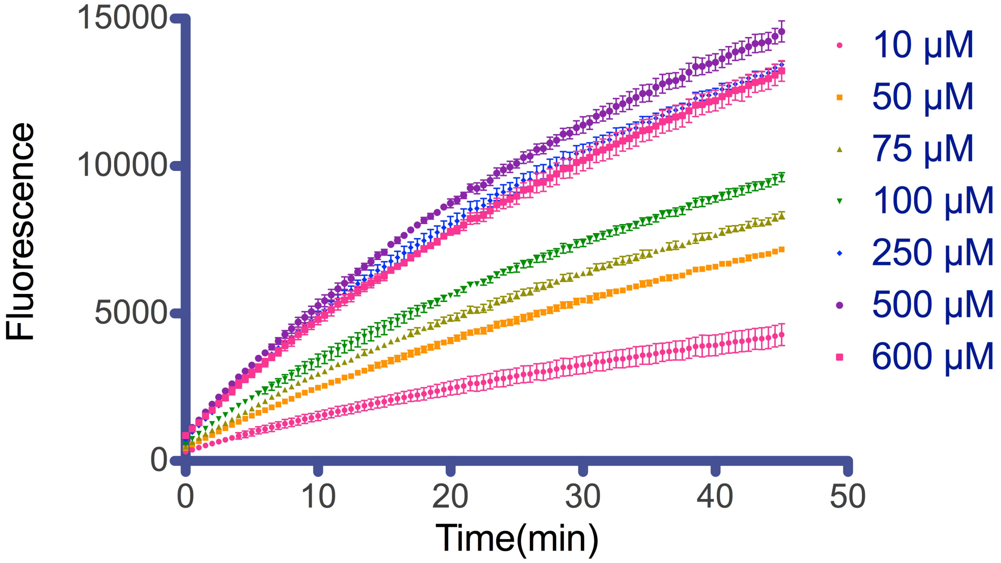

Supplementary Figure 40: Raw activity for free DNA-modified β-Gal (0.5 nM) with different concentration of, ranging from 10 μM to 600 μM RBG, monitoring fluorescence at 590 nm (excitation 532 nm). Error bars were calculated from the standard deviation of at least three replicates.

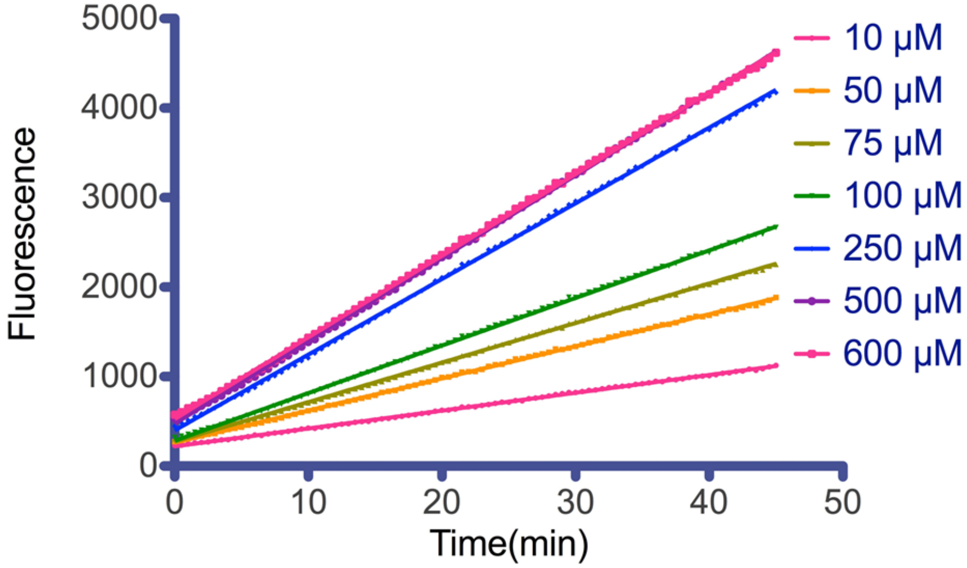

Supplementary Figure 41: Raw activity for full-cage [β-Gal] (0.5 nM) with different concentration of, ranging from 10 μM to 600 μM RBG, monitoring fluorescence at 590 nm (excitation 532 nm). Error bars were calculated from the standard deviation of at least three replicates.

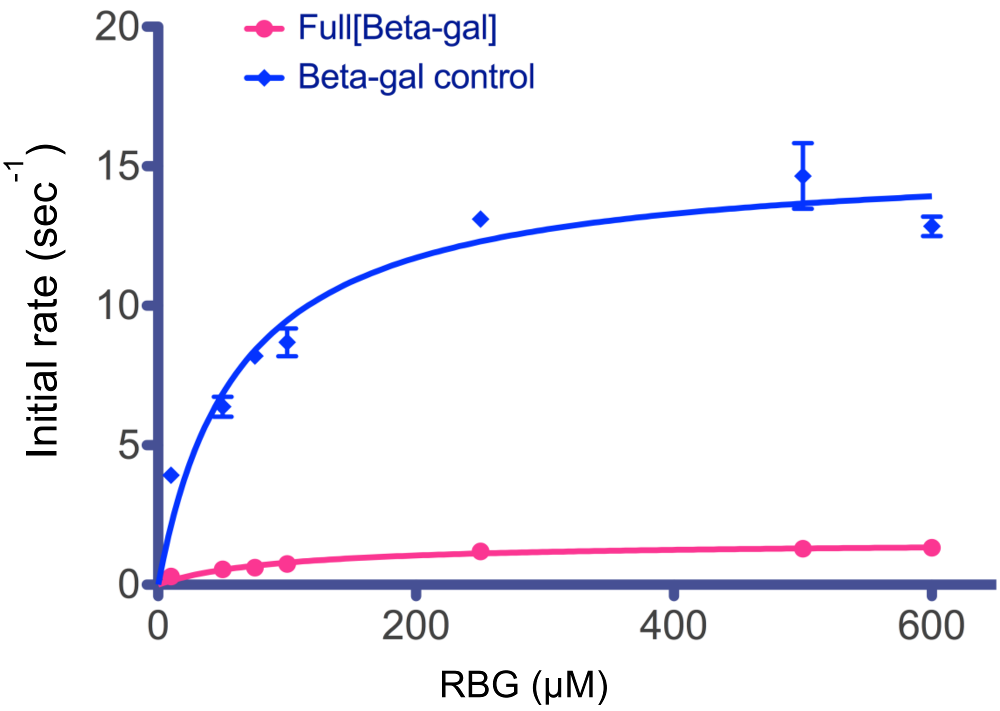

	K_M (µM)	k_{cat} (s^{-1})
**Full[β-Gal]**	95.5±18.9	1.6±0.1
β-Gal **control**	58.7±16.0	8.5±0.6

Supplementary Figure 42: Michaelis-Menten plot of full-cage [β-Gal] (red circle), compared with that of the fresh free MDH enzyme (blue square) using RBG as the substrate. The solid line is the fitting curve using the Michaelis-Menten model. Enzyme assay condition: 0.5 nM enzyme or DNA cage encapsulated enzyme, with different concentration of RBG, ranging from 10 µM to 600 µM, in TBS buffer (pH 7.5, 1 mM $MgCl_2$) monitoring fluorescence at 532/590 nm. The table lists the fitting parameters. DNA encapsulation of the enzyme caused a ~1.6-fold increase in K_M and a ~81% decrease in k_{cat}. Error bars were calculated from the standard deviation of at least three replicates.

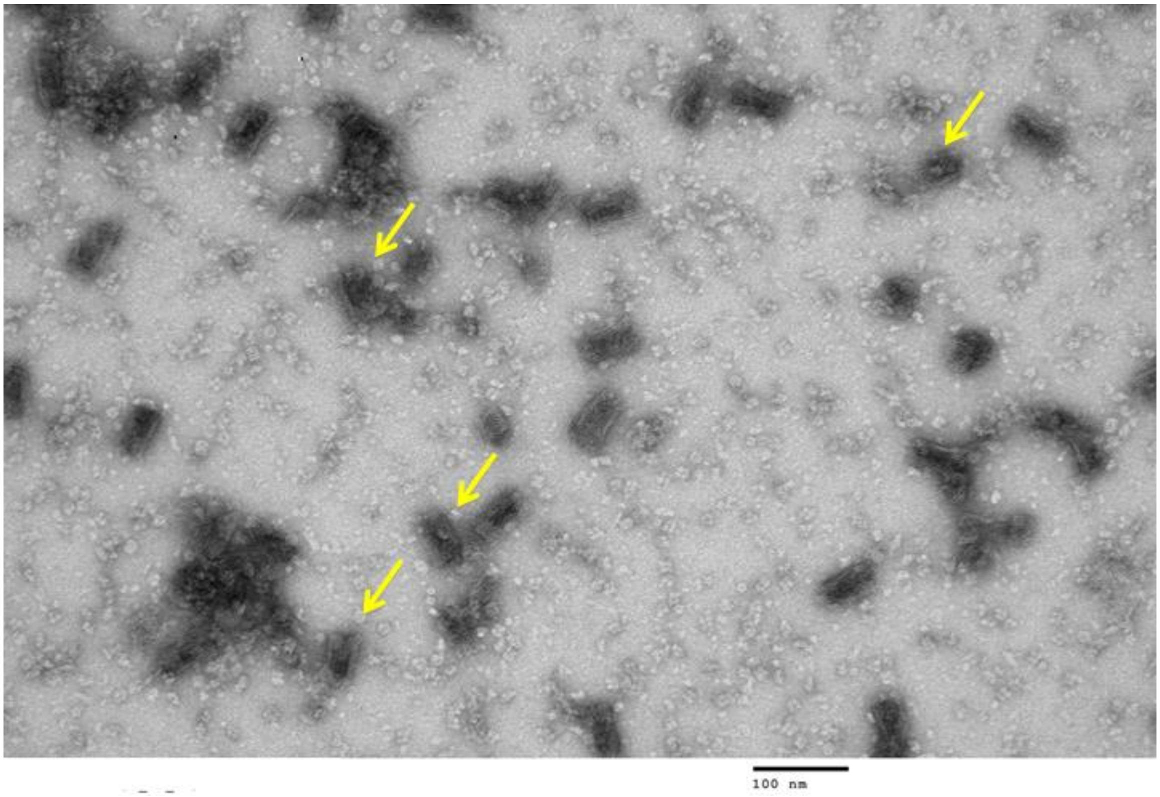

Supplementary Figure 43: TEM image for the DNA full-cages with β-Gal inside (yellow arrow indicates DNA cage with enzyme inside).

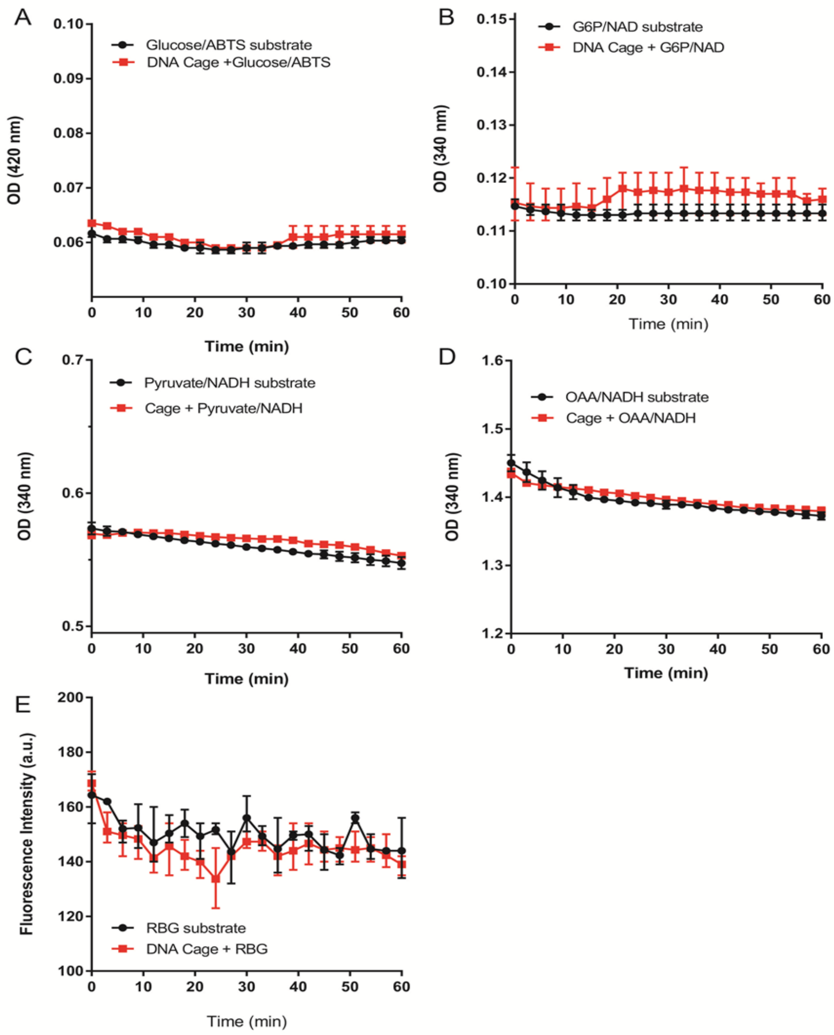

Supplementary Figure 44: Control experiments in which DNA cages were incubated with enzyme substrates. (A) Red curve: 1 nM Cage was incubated with 1 mM glucose and 2 mM ABTS (GOx/HRP substrates) in 1×TBS, pH 7.5; Black: Autocatalysis of 1 mM glucose and 2 mM ABTS (GOx/HRP substrates) in 1×TBS, pH 7.5. (B) Red: 0.5 nM Cage was incubated with 1 mM glucose-6-phosphate and 1 mM NAD^+ (G6PDH substrates) in 1×TBS, pH 7.5; Black: Autocatalysis of 1 mM glucose-6-phosphate and 1 mM NAD^+ in 1×TBS, pH 7.5. (C) Red: 0.5 nM Cage was incubated with 2 mM pyruvate and 0.25 mM NADH (LDH substrates) in 1×TBS, pH 7.5; Black: Autocatalysis of 2 mM pyruvate and 0.25 mM NADH in 1×TBS, pH 7.5. (D) Red: 0.5 nM Cage was incubated with 2 mM oxaloacetate (OAA) and 1 mM NADH (MDH substrates) in 1×TBS, pH 7.5; Black: Autocatalysis of 2 mM OAA and 1 mM NADH in 1×TBS, pH 7.5. (E) Red: 0.5 nM Cage was incubated with 100 μM resorufin beta-D-galactopyranoside (RBG, β-Gal substrate) in 1×TBS, pH 7.5; Black: Autocatalysis of 100 μM RBG in 1×TBS, pH 7.5, 532 nm (excitation)/590 nm (emission). Error bars were calculated from the standard deviation of at least three replicates. All above results indicate that DNA cages at our experimental concentrations do not significantly catalyze substrate conversion.

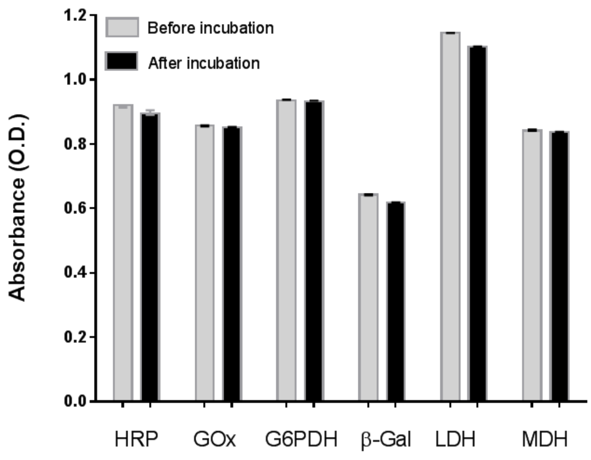

Supplementary Figure 45: Test of the nonspecific adsorption of enzymes onto a plastic 96-well plate. Enzyme concentrations are quantified by UV-VIS spectrometer using the following extinction coefficients: HRP ($E_{405\ nm}$ ~ 100,000 M^{-1} cm^{-1}), GOx ($E_{280\ nm}$ ~ 267,200 M^{-1} cm^{-1}), G6pDH ($E_{280\ nm}$ ~ 118,450 M^{-1} cm^{-1}), β-Gal ($E_{280\ nm}$ ~ 972,093 M^{-1} cm^{-1}), LDH ($E_{280\ nm}$ ~ 202,640 M^{-1} cm^{-1}), MDH ($E_{280\ nm}$ ~ 19,600 M^{-1} cm^{-1}). The UV-Vis absorbance of 100 µL of each enzyme solution was measured before adding to the plates, as well as after one hour incubation within the plates in the dark. These conditions are the same as those of the enzyme activity assay. As shown in the Figure, all enzyme solutions showed only a very slight decrease in absorbance after incubation in the plates, suggesting very weak nonspecific adsorption of enzymes onto the plastic plates. Error bars were calculated from the standard deviation of at least three replicates.

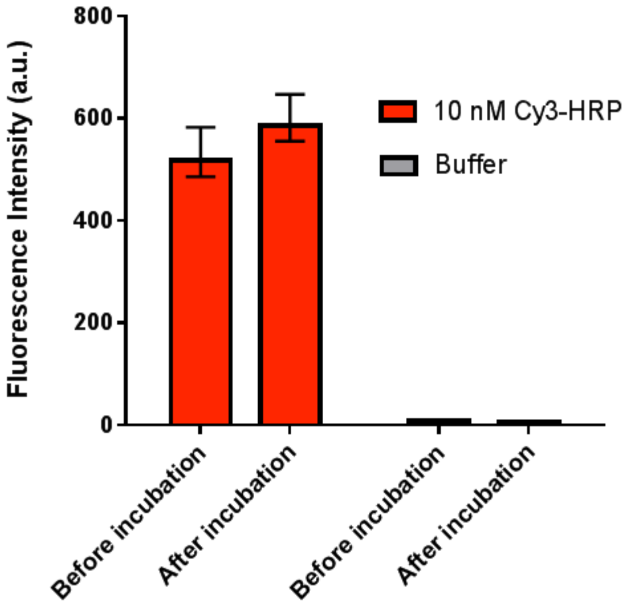

Supplementary Figure 46: Testing for nonspecific adsorption of low nanomolar concentrations of enzymes onto plastic 96-well plates was tested using Cy3-labeled HRP. 100 μL of 10 nM Cy3-labeled HRP was assayed for fluorescence intensity, and then the plate was incubated inside a plate reader for one hour. The remaining fluorescence intensity was tested again. A slight increase of fluorescence intensity was observed, possibly due to the buffer evaporation during the incubation. This result suggests that there is very little nonspecific adsorption of Cy3-HRP onto the 96-well plate. Error bars were calculated from the standard deviation of at least three replicates.

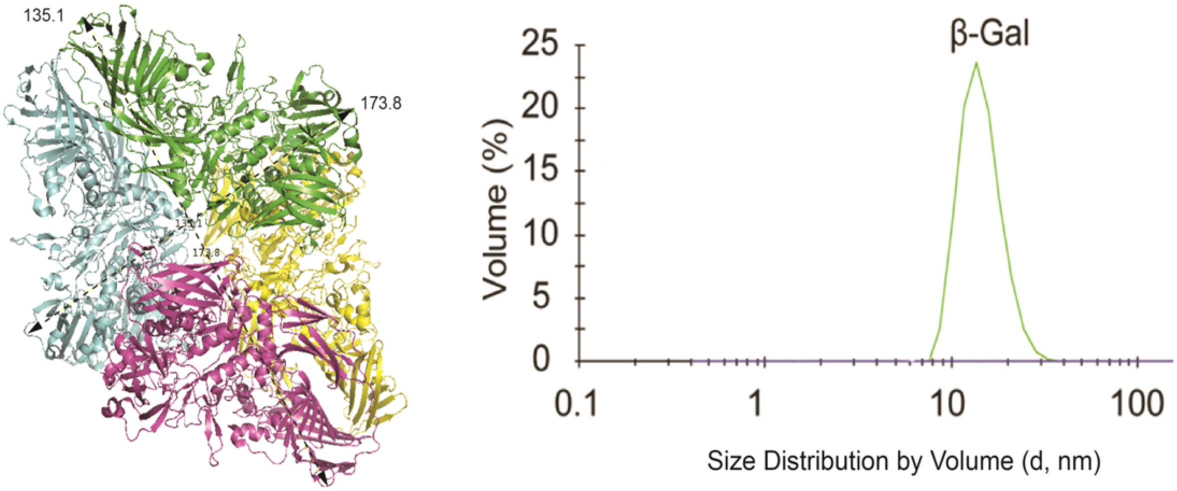

Supplementary Figure 47: The crystal structure of β-Gal shows its dimensions to be ~ 17 nm × 14 nm (left) (Jacobson, R. H. et al. *Nature* **369**, 761-766 (1994)). Dynamic Light Scattering measures a hydrodynamic diameter of ~ 14 – 18 nm.

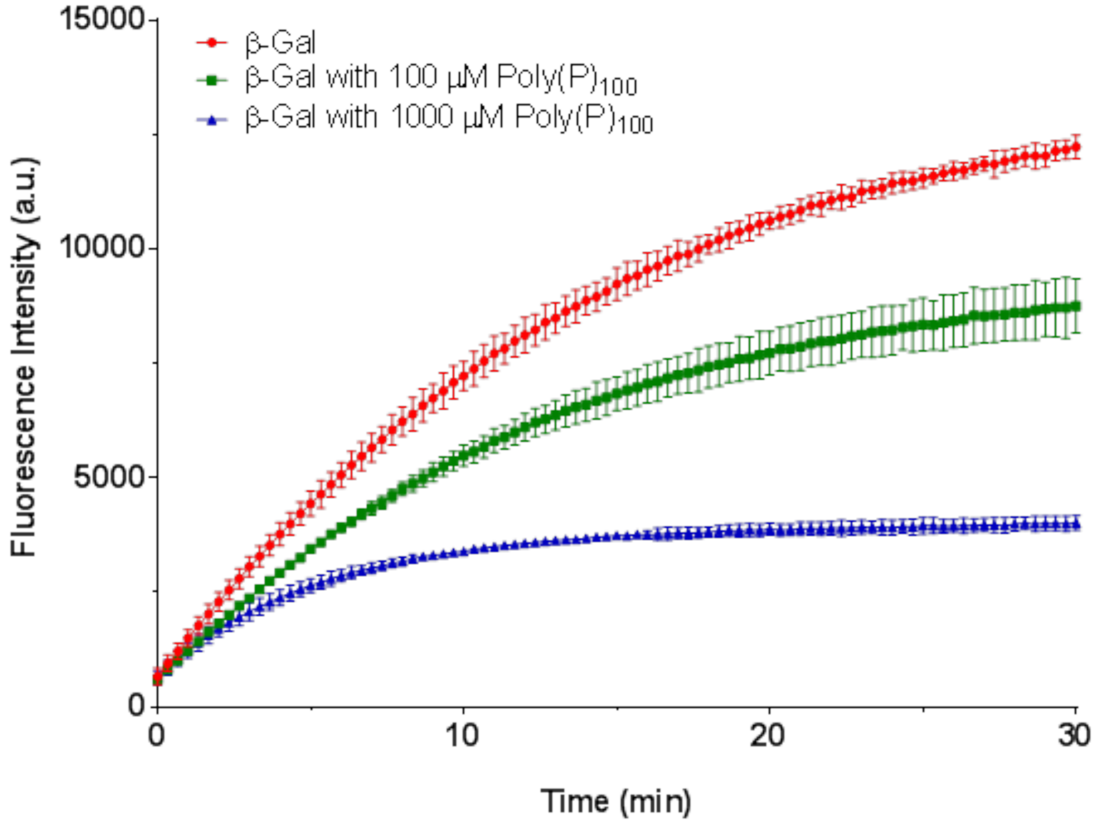

Supplementary Figure 48: Inhibition of β-Gal activity by 100-mer polyphosphate (Poly(P)$_{100}$) in solution. Assay condition: 0.25 nM β-Gal and 100 μM RBG in pH 7.4, 50 mM HEPES buffer. For inhibition assay, β -Gal was first incubated with Poly(P)$_{100}$ for half an hour, then RBG substrate was added before measuring the activity. The control β-Gal was run at the same condition except for the incubation with buffer for half an hour. The activity of β-Gal was significantly inhibited by 1000 μM Poly(P)100. Error bars were calculated from the standard deviation of at least three replicates.

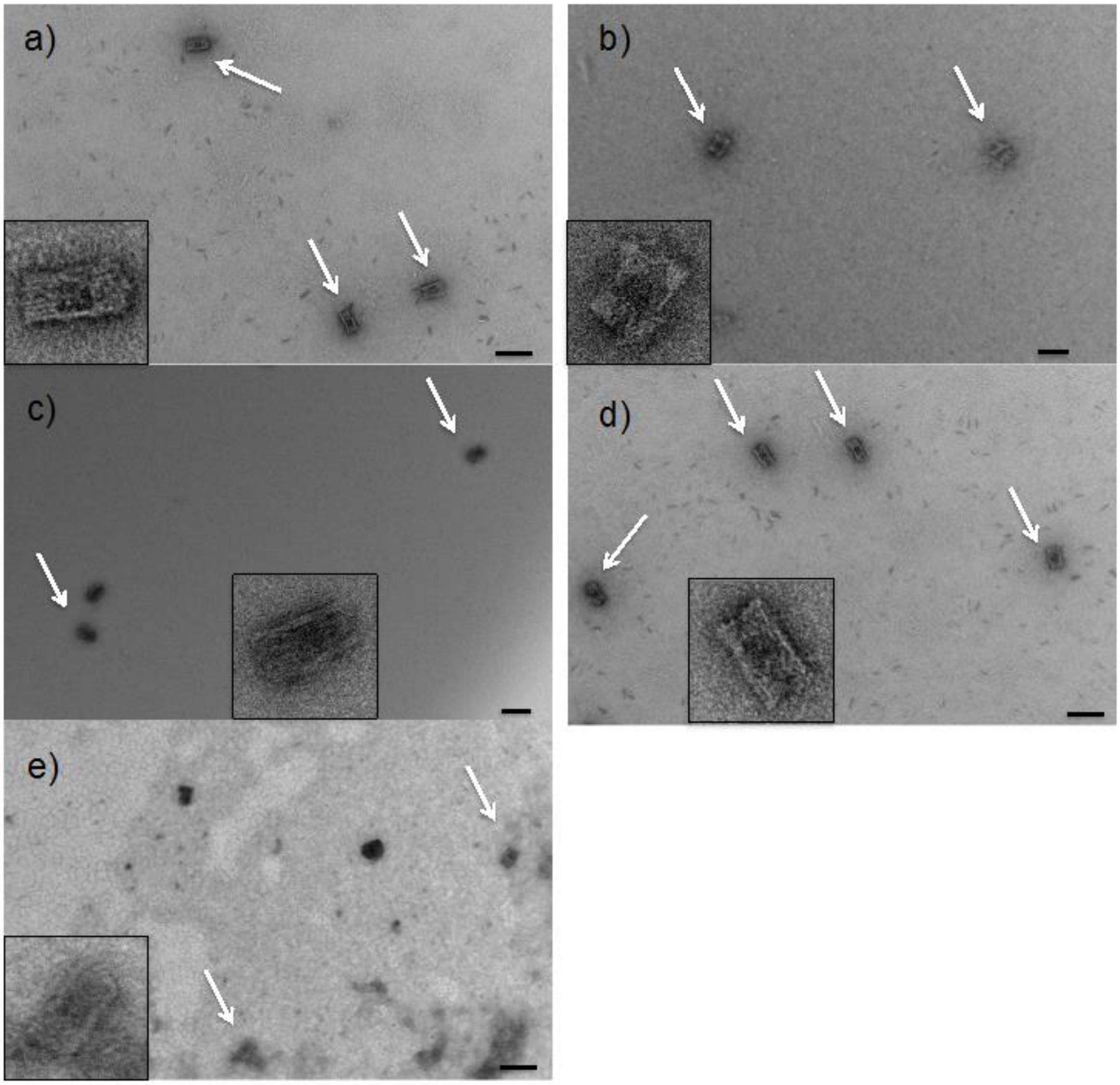

Supplementary Figure 49: TEM image of DNA cages after 1h incubation with a) GOx-HRP enzymatic reaction (conditions: 50 mM HEPES, pH 7.5, 1mM $MgCl_2$, 1mM glucose, 2mM ABTS, 1nM GOx-HRP, 0.5nM DNA cage), b) G6pDH enzyme reaction (conditions: 50 mM HEPES, pH 7.5, 1mM $MgCl_2$, 1mM glucose-6-phosphate, 1mM NAD^+, 1nM G6pDH, 0.5nM DNA cage), c) MDH enzyme reaction (conditions: 50 mM HEPES, pH 7.5, 1mM $MgCl_2$, 2mM OAA, 1mM NADH, 1nM MDH, 0.5nM DNA cage), d) LDH enzyme reaction (conditions: 50 mM HEPES, pH 7.5, 1mM $MgCl_2$, 2mM pyruvate, 1mM NADH, 1nM LDH, 0.5nM DNA cage), e) β-gal enzyme reaction (conditions: 50 mM HEPES, pH 7.5, 1mM $MgCl_2$, 1mM RBG 1nM Beta-gal, 0.5nM DNA cage). (Scale bars: 50 nm)

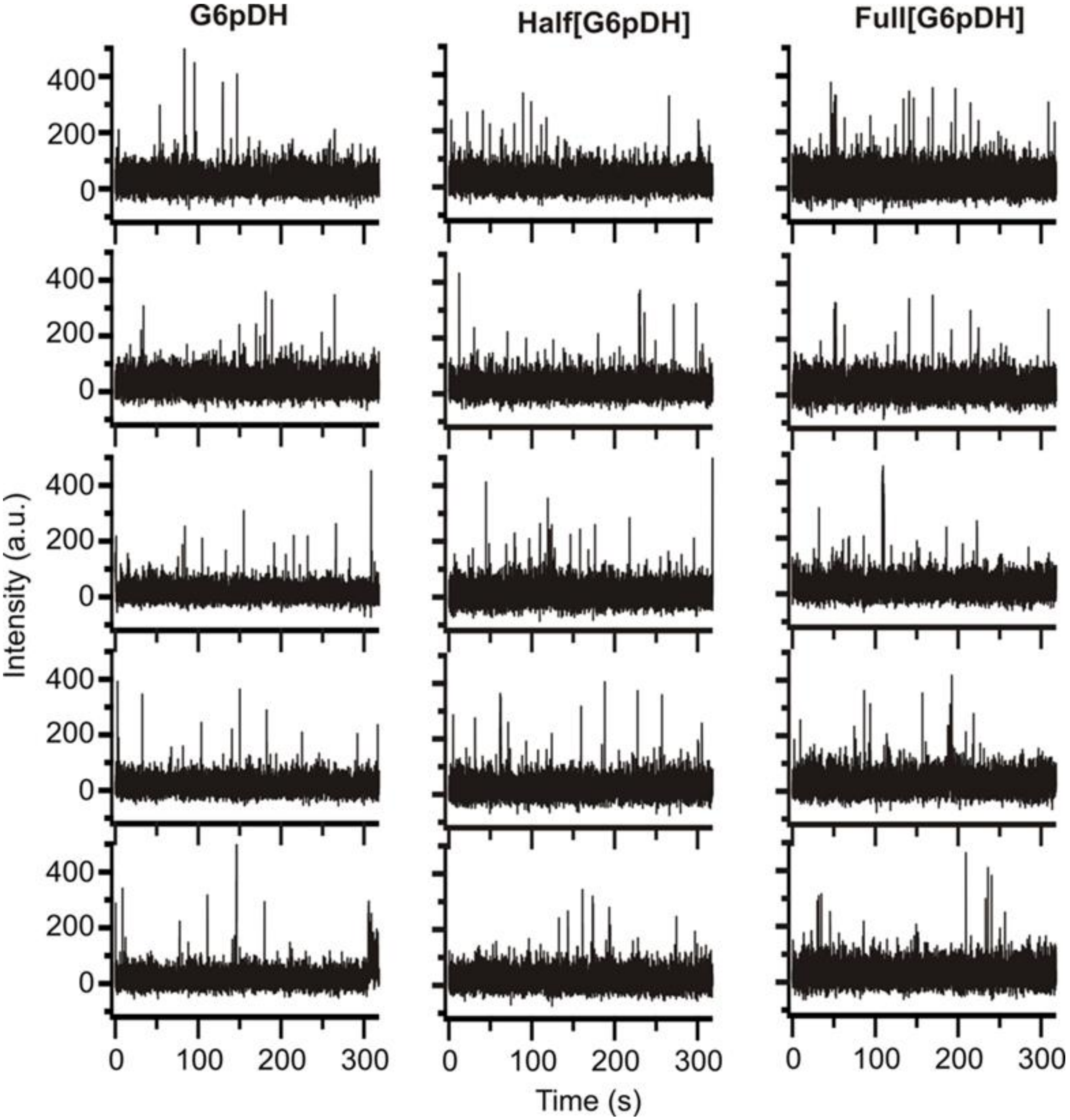

Supplementary Figure 50: **Raw enzyme activity data of single G6pDH molecules.** Representative fluorescence-time traces of free-, half-cage and full-cage G6pDH. Five representative molecules are shown for each sample. The fluorescence intensity of enzyme reaction on the microscope slide was recorded for ~5 min at 35 ms time resolution. The average spikes per molecule for different samples are compared in Fig. 5. All experiments were carried out at room temperature in 1× TBS buffer in presence of 1 mM Mg^{2+}, pH 7.5 (Supplementary Table S4).

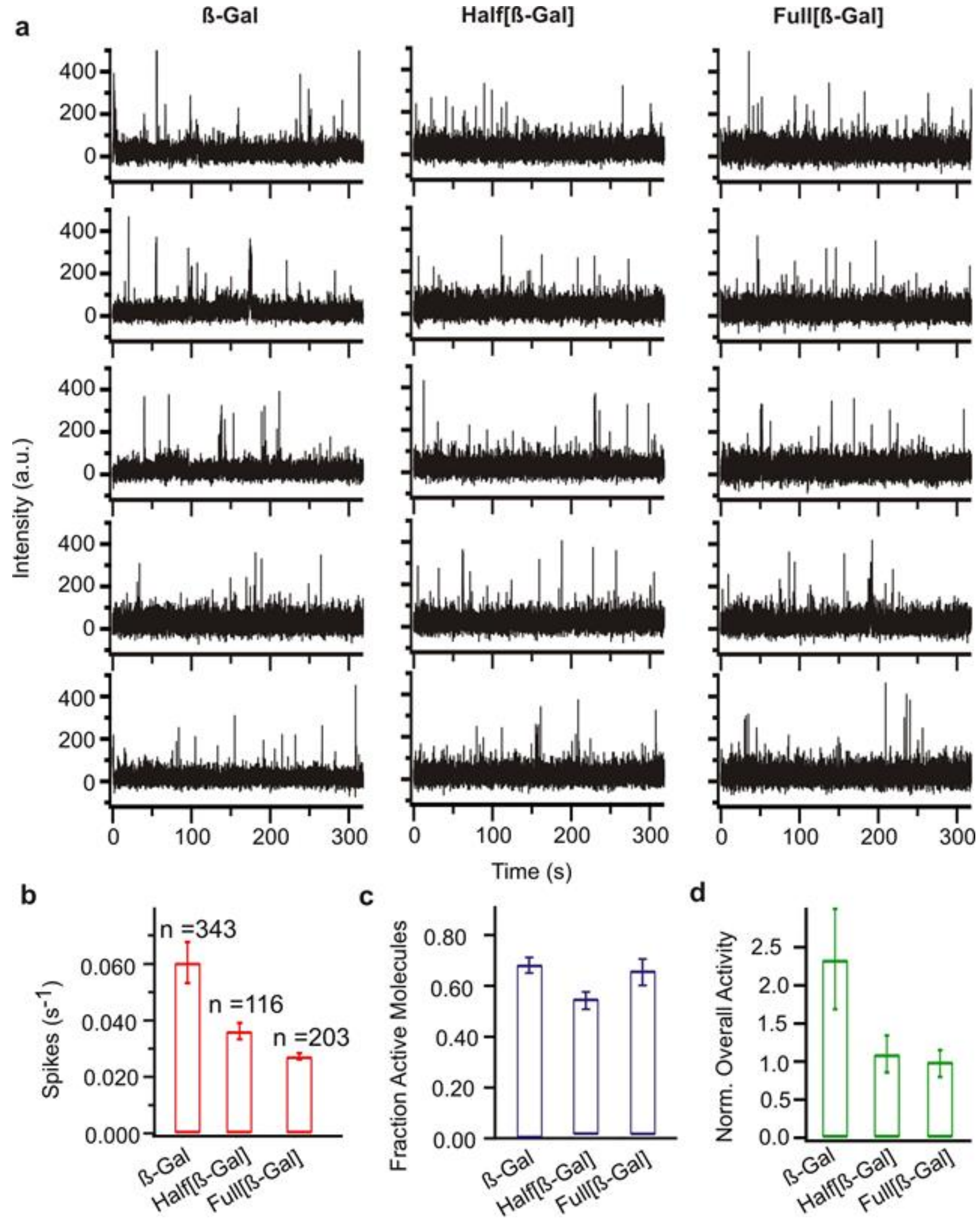

Supplementary Figure 51: **Enzyme activity data of single β -Gal molecules.** (**a**) Representative raw fluorescence-time traces of free-, half-cage and full-cage β -Gal. Five representative molecules are shown for each sample. The fluorescence intensity of enzyme reaction on the microscope slide was recorded for ~5 min at 35 ms time resolution. (**b,c,d)** Statistics of spike frequency, fraction of active molecules, and overall observed enzyme activity. The number of active molecules analyzed is denoted by 'n' in **b**. The standard deviations for spike frequency and fraction of active molecules were calculated after randomly assigning the active molecules into three groups. The standard deviation for the normalized overall activity was estimated from the propagation of errors. All experiments were carried out at room temperature in 1× TBS buffer, pH 7.5 in presence of 1 mM Mg^{2+} and 10% (w/v) PEG 8000.

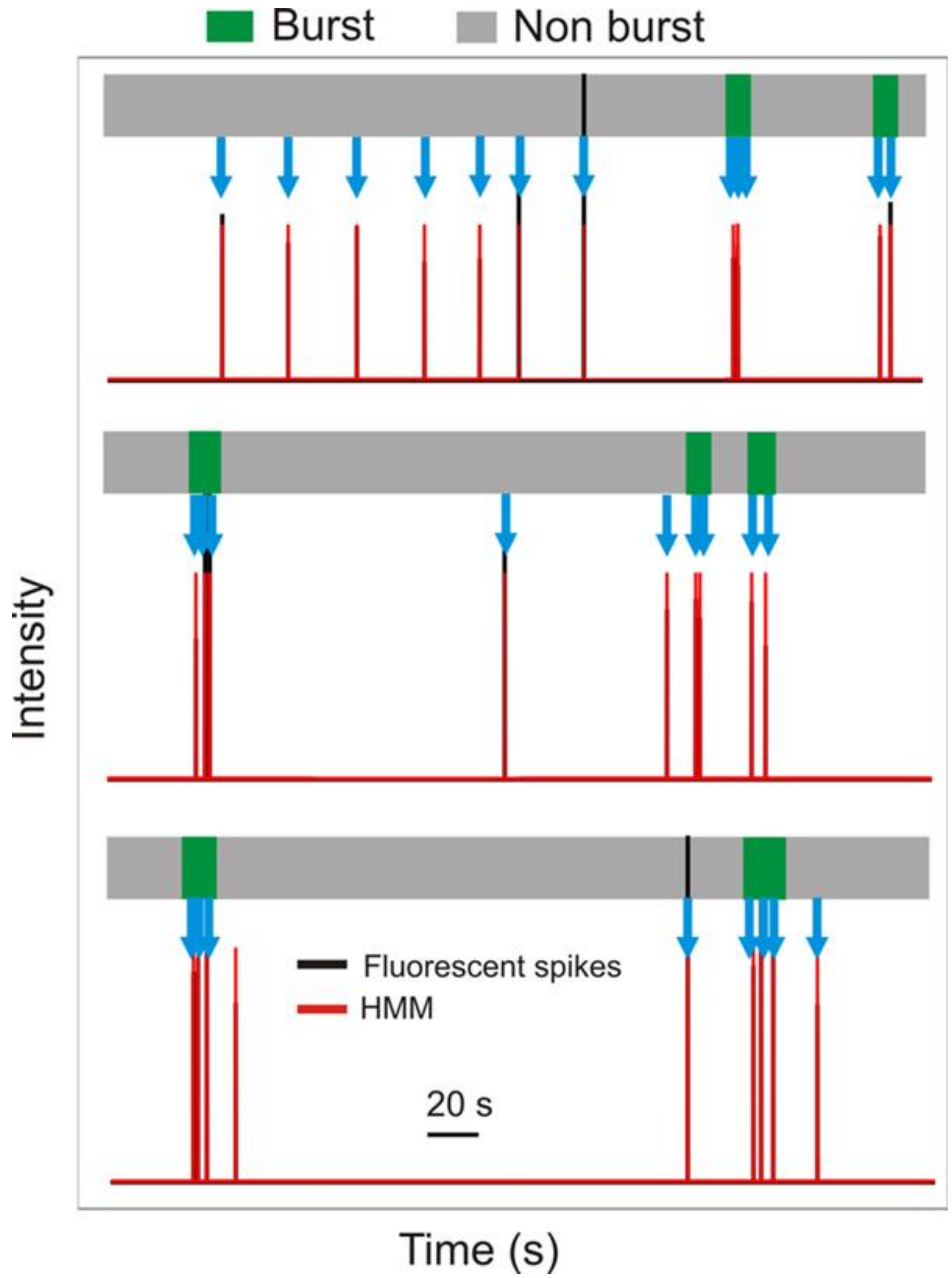

Supplementary Figure 52: Representative intensity-time traces (black) of full-cage enzyme after background correction and Hidden Markov Model (HMM) idealization to a two-state model (red). The fluorescence-time traces of the enzyme reaction on the microscope slide were recorded at 35 ms time resolution over ~5 min.

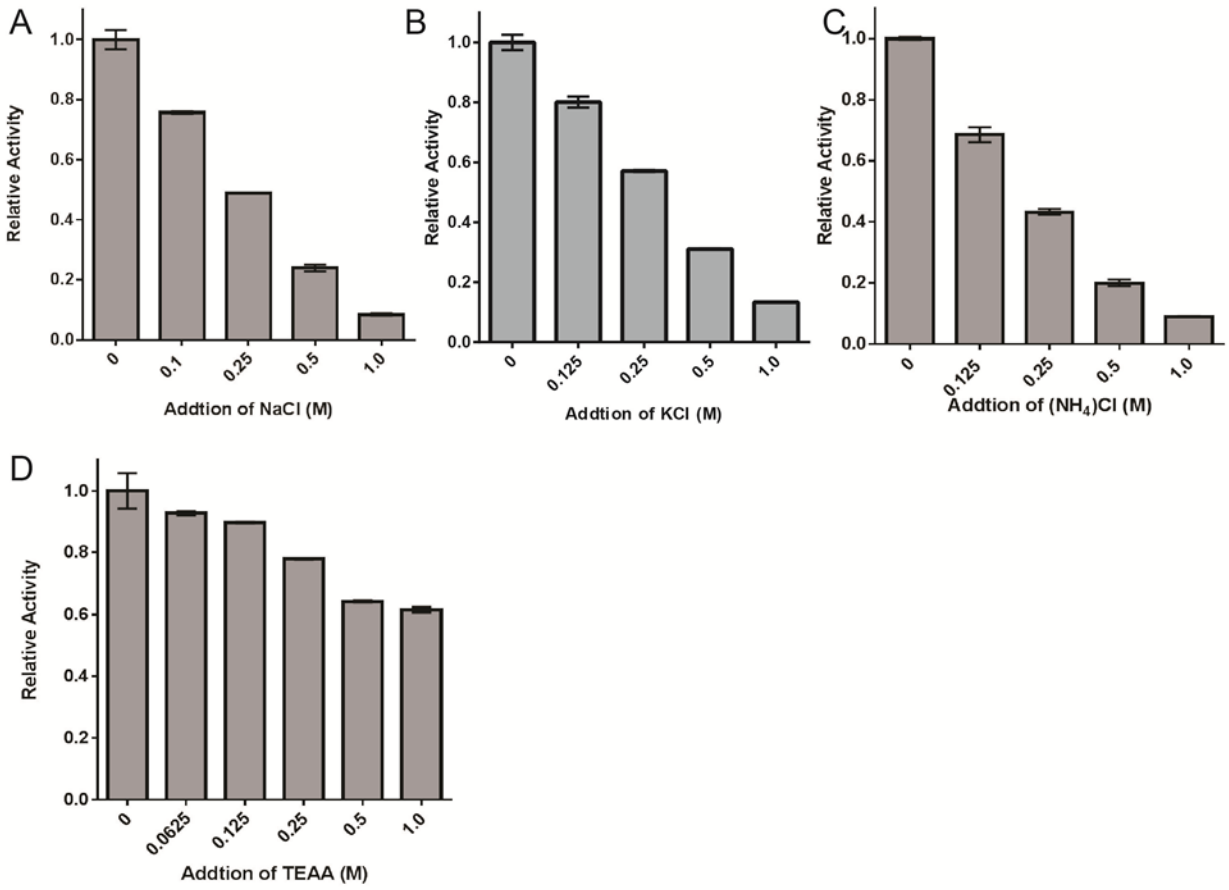

Supplementary Figure 53: Titrations showing the effects of (A) NaCl, (B) KCl, (C) NH_4Cl and (D) Triethylammonium acetate (TEAA) on the activity of free G6pDH. Assay conditions: 0.5 nM enzyme was incubated with a series of ion concentrations from low to high. Enzyme activity was monitored by absorbance at 340nm with the addition of 1 mM Glucose-6-phosphate and 1 mM NAD^+ in 1×TBS buffer (pH 7.5). The results show that high concentration of salts containing small cations such as Na^+, K^+ and NH_4^+ significantly reduce the activity of G6pDH, possibly due to the chaotropic ion effect that disrupts hydrogen-bonded water structures as reported in the previous studies (Zhao, H. *Journal of Molecular Catalysis B: Enzymatic* 2005, *37*, 16; Leberman, R. and Soper, A. K. *Nature* 1995, *378*, 364.). Conversely, the salt containing a bulky organic cation (kosmotropic), triethylammonium, does not strongly inhibit enzyme activity, even at high concentrations. Error bars were calculated from the standard deviation of at least three replicates.

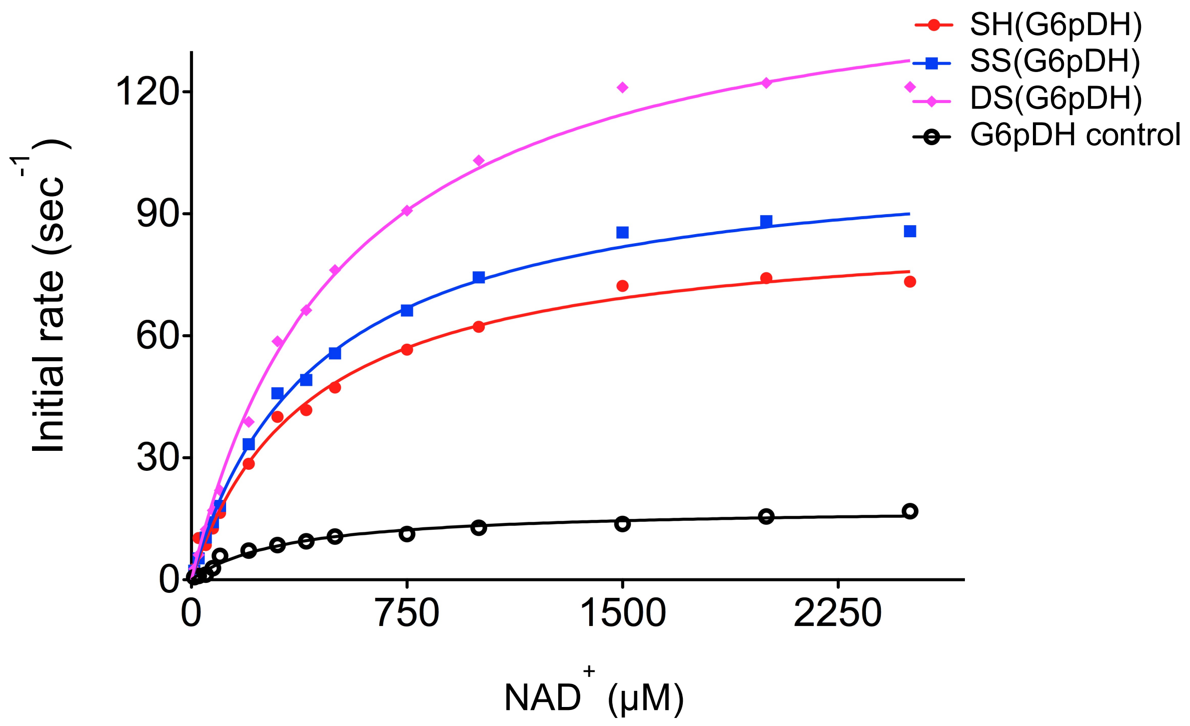

	K_M (μM)	k_{cat} (s^{-1})
**SH-G6pDH**	411±32	520±10
**SS-G6pDH**	436±26	620±10
**DS-G6pDH**	527±37	900±20
**G6pDH control**	340±47	100±10

Supplementary Figure 54: Comparison of G6pDH activity inside three different DNA full-cages, compared with that of free G6pDH, using NAD^+ as the varying substrate. The SH, SS and DS cages are described in the main text. Enzyme assay conditions: 0.5 nM enzyme or DNA-cage-encapsulated enzyme, 1 mM glucose 6-phosphate, with different concentration of NAD^+ ranging from 10 μM to 2500 μM, in 1×TBS buffer (pH 7.5, 1 mM $MgCl_2$) monitoring absorbance at 340 nm. The table lists the fit parameters. Encapsulation of the enzyme in different DNA full-cages caused a ~1.2- to 1.5-fold increase in K_M and a ~5- to 9-fold increase in k_{cat}.

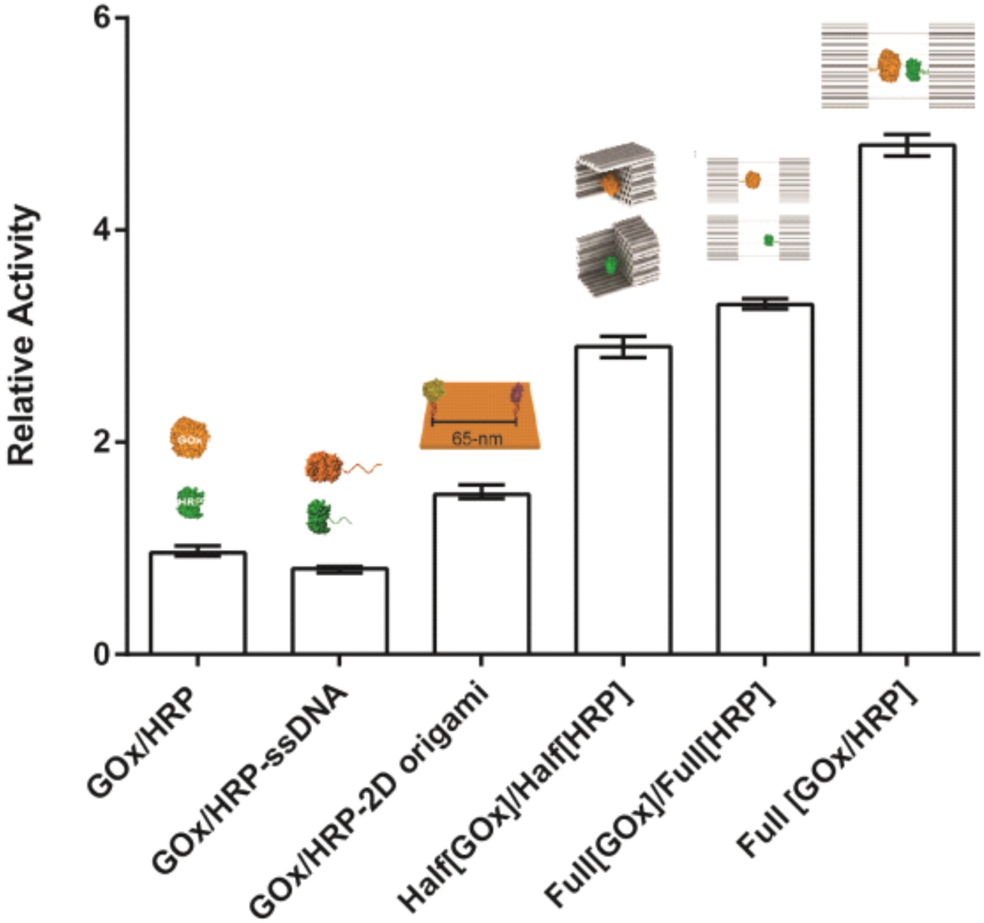

Supplementary Figure 55: The relative activity of a GOx/HRP pair when attached to a variety of DNA scaffolds: enzyme wildtypes (GOx/HRP), ssDNA (GOx/HRP-ssDNA), 2D rectangular DNA origami (GOx/HRP-2D origami), separate 3D half cages (Halg[GOx]/Half[HRP]), separate full cages (Full[GOx]/Full[HRP]) and the same full cage (Full [GOx/HRP]). Enzyme activity is positively correlated to the density of DNA helices within the scaffolds, and partially or fully caged enzymes exhibit activity several-fold higher than that of free and unconjugated enzymes. Error bars were calculated from the standard deviation of at least three replicates. The value for GOx/HRP-2D origami is extracted from our previously published article (Fu, J. et al. *JACS* **2012**, *134*, 5516–5519).

We concluded that the boosted activities of Full[GOx/HRP] cannot be simply attributed to a single factor of DNA density or close proximity, but may be induced by both of the high DNA density and close proximity within a DNA cage.

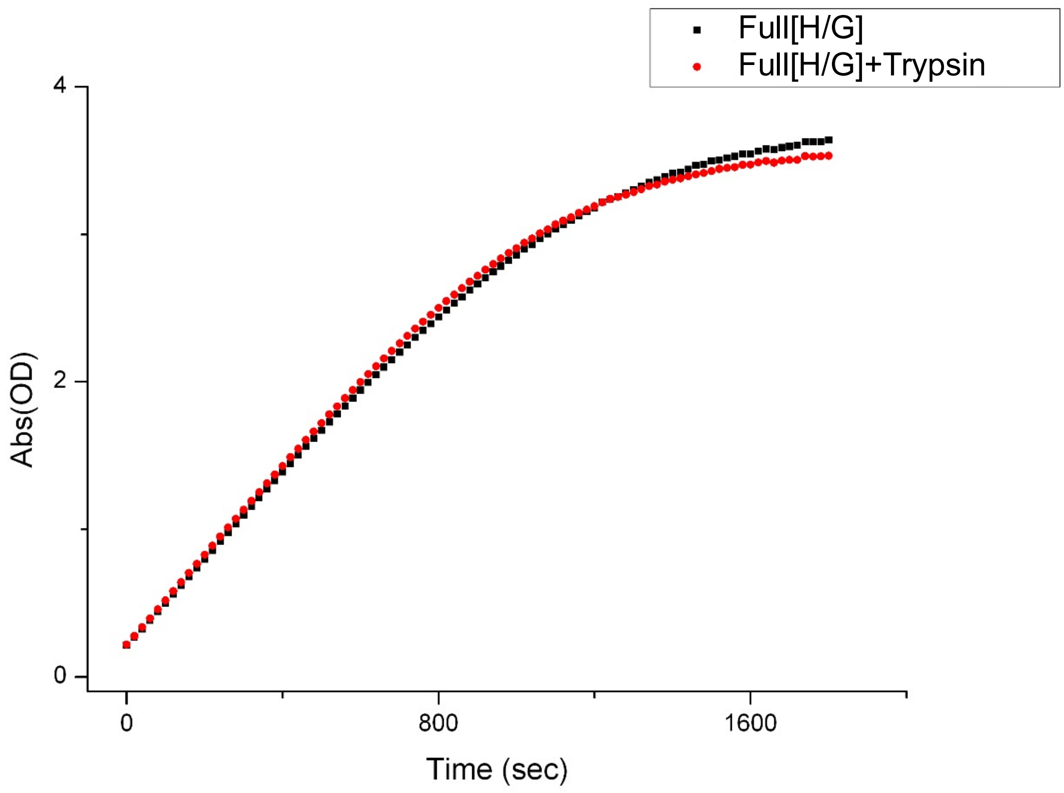

Supplementary Figure 56: Raw activity for full-cage [HRP/GOx] (0.5 nM) before and after trypsin digestion for 24 hours at 37 °C in 1 × TBS buffer (pH 7.5).

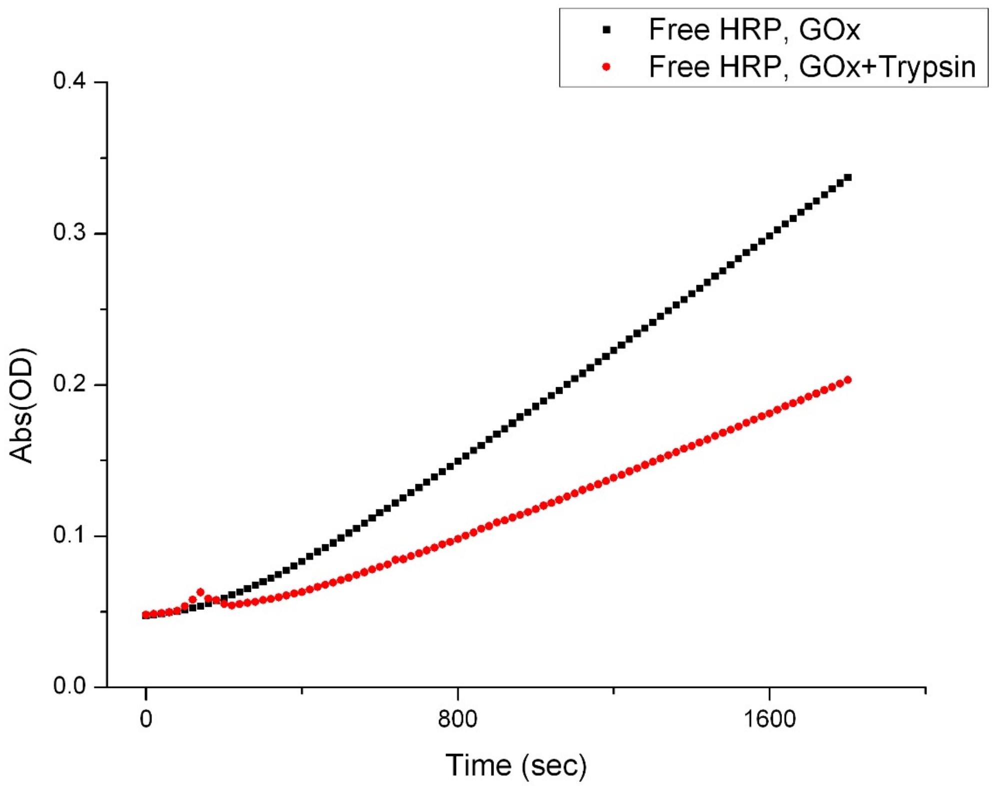

Supplementary Figure 57: Raw activity of a free pair of HRP and GOx (0.5 nM) before and after trypsin digestion for 24 hours at 37 °C in 1 × TBS buffer (pH 7.5).

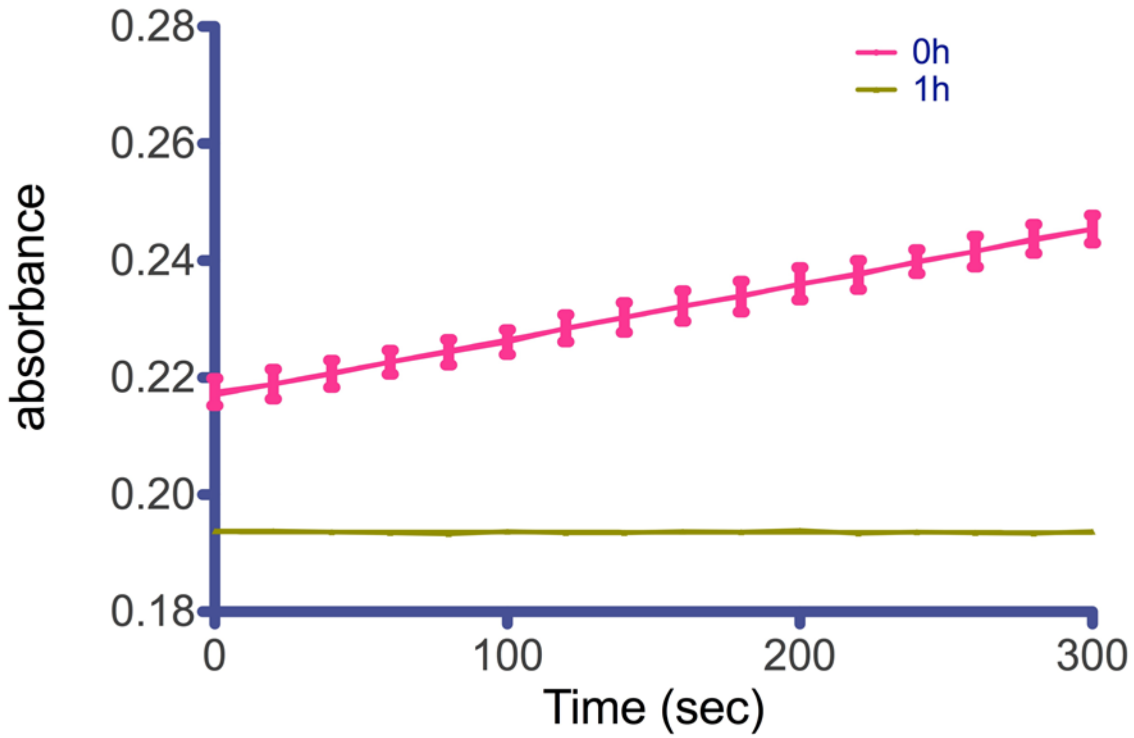

Supplementary Figure 58: Raw activity data for free G6pDH (0.5nM) before and after trypsin digestion for 1h at 37 °C in 1 × TBS buffer (pH 7.5). Error bars were calculated from the standard deviation of at least three replicates.

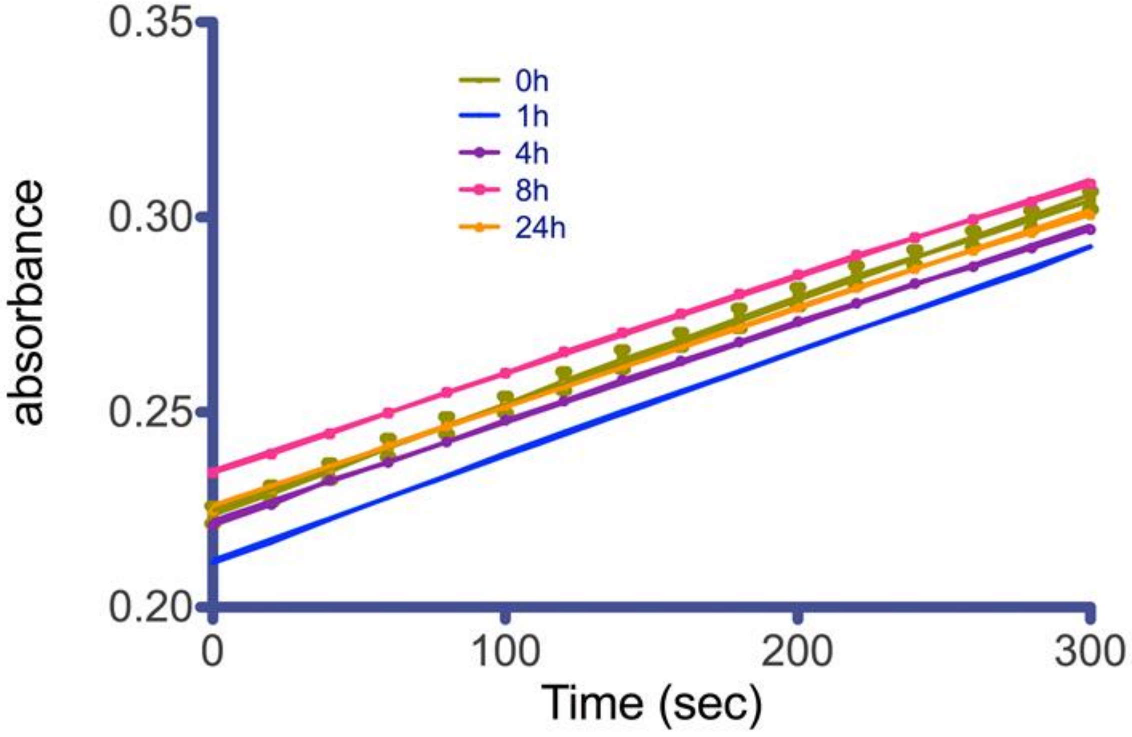

Supplementary Figure 59: Raw activity data for Full[G6pDH] (0.5nM) timecourse trypsin digestion from 0 h to 24 h at 37 °C. Error bars were calculated from the standard deviation of at least three replicates.

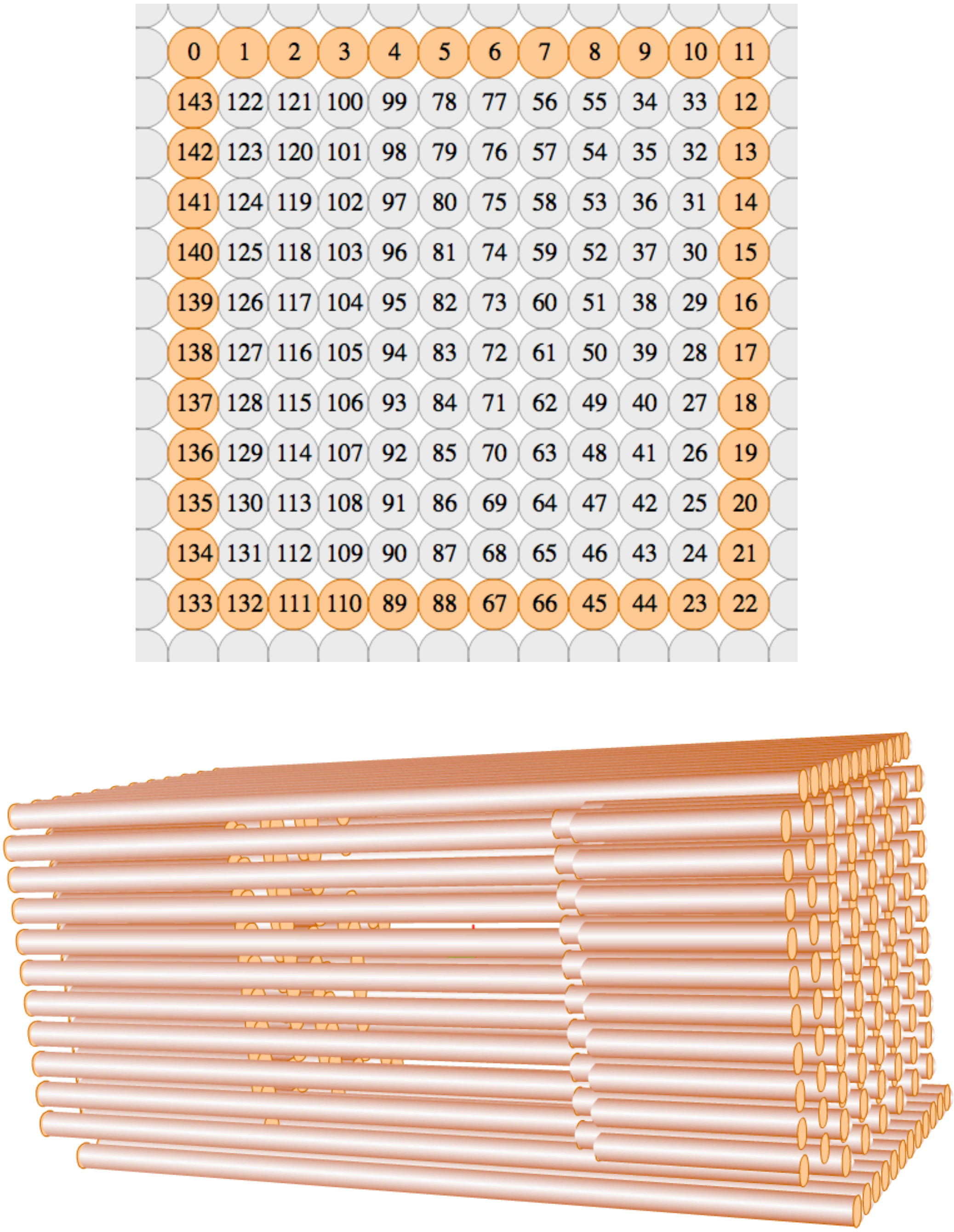

Supplementary Figure 60: Design of SS cage (square lattice arrangement), including cross-sectional view and 3D view.

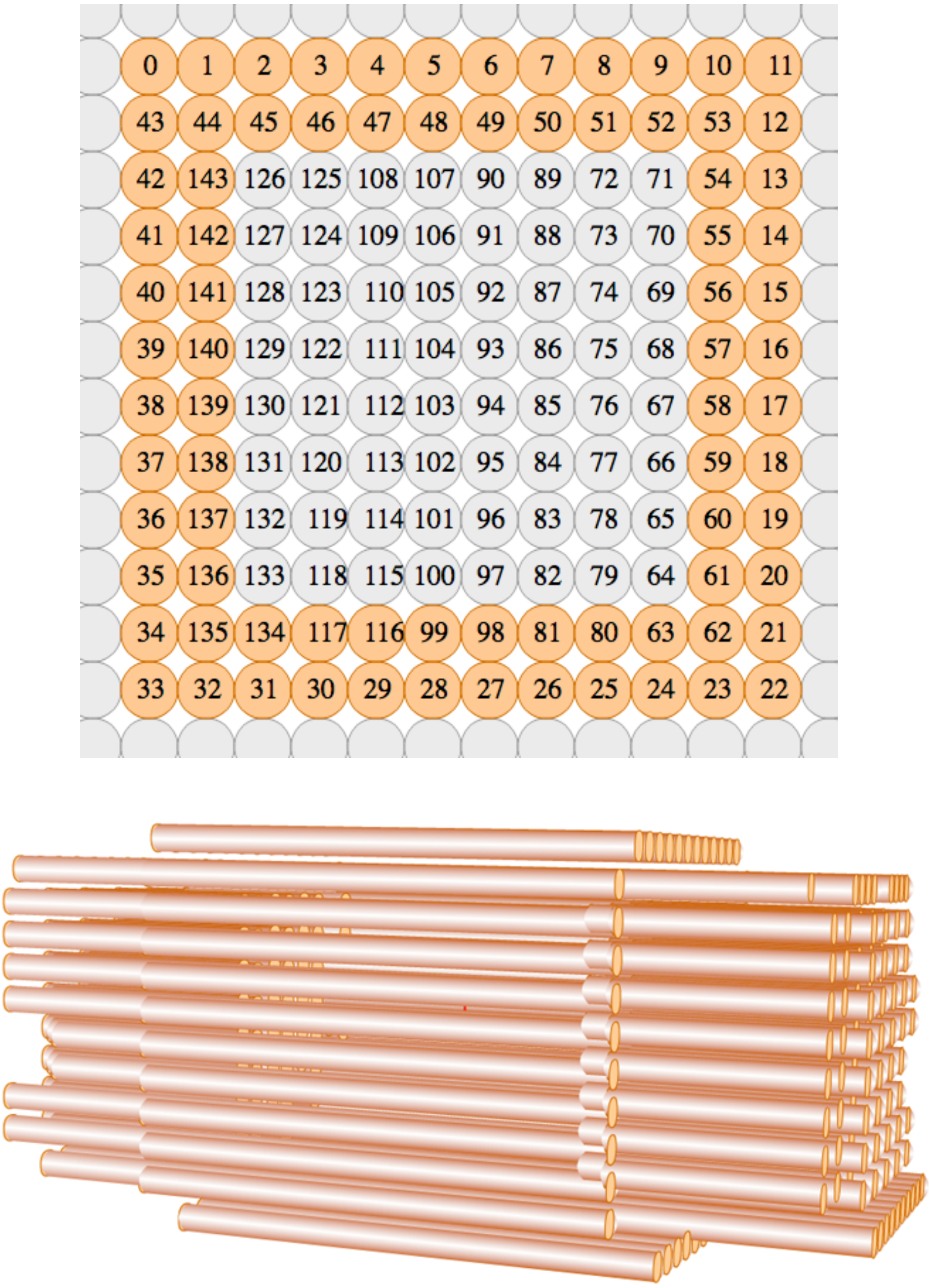

Supplementary Figure 61: Design of DS cage (square lattice arrangement), including cross-sectional view and 3D view.

Supplementary Tables

Supplementary Table 1. Estimation of the concentration and DNA labeling ratio of the purified DNA-conjugated enzymes by measuring the absorbance at 260 and 280 nm

DNA	A260/A280	ε260 (M^{-1} cm^{-1})	ε280 (M^{-1} cm^{-1})	Protein	A260/A280	ε260 (M^{-1} cm^{-1})	ε280 (M^{-1} cm^{-1})	Sample	A260/A280	A260	A280	DNA - to-Protein Ratio	Protein Conc. (uM)	Dye (uM)
P1-Cy3	1.27	115200	90709	Gox	0.63	168336	267200	GOx-P1-Cy3	1.18	13.50	14.10	3.09	25.77	37.00
P1-Cy3	1.27	115200	90709	β-Gal	0.59	573534.9	972093	β-Gal-P1-Cy3	0.63	1.34	2.11	0.74	2.03	1.10
P1-Cy3	1.27	115200	90709	G6pDH	0.52	61594	118450	G6pDH-P1-Cy3	1.00	11.15	11.17	2.30	34.17	53
P2-AF647	1.60	130100	81313	MDH	0.72	14112	19600	MDH-P2-AF647	1.49	1.47	0.99	1.63	6.49	8
P2-AF647	1.60	130100	81313	LDH	0.57	115504.8	202640	LDH-P2-AF647	0.83	2.83	3.41	0.84	12.59	22

DNA	A260/A280	ε260 (M^{-1} cm^{-1})	ε280 (M^{-1} cm^{-1})	Protein	A260/A405	ε260 (M^{-1} cm^{-1})	ε405 (M^{-1} cm^{-1})	Conjugates	A260/A280	A260	A280	A405	DNA conc. (uM)	DNA - to-Protein Ratio	Protein Conc. (uM)	Dye
P1-Cy3	1.27	115200	90709	HRP	0.38	38000	100000	HRP-P1-Cy3	1.24	7.32	5.89	4.53	45.50	1.01	45.26	81 uM

$$A_{260}(DNA - protein) = \varepsilon_{260}(protein) * Conc.(protein) + \varepsilon_{260}\ (DNA) * Conc.(DNA)$$

$$A_{280}(DNA - protein) = \varepsilon_{280}(protein) * Conc.(protein) + \varepsilon_{280}\ (DNA) * Conc.(DNA)$$

$$Ratio\left(\frac{DNA}{protein}\right) = \frac{Conc.(DNA)}{Conc.(protein)}$$

Concentration of HRP-P1-Cy3 was estimated by the unique absorbance at 405 nm.

Supplementary Table 2. Enzyme encapsulation efficiency calculation

	N	N_{coloc}	N_{right}	N_{coloc}/N_{right}
**HRP**	176	156	165	0.94
**GOx**	205	197	201	0.98
**G6pDH**	218	209	214	0.98
**LDH**	1229	826	1008	0.82
**MDH**	363	335	348	0.96
**β-Gal**	284	115	179	0.64

Enzyme encapsulation was calculated by taking the ratio of the number of colocalized molecules (i.e., both enzyme and **right** half-cage) to the total number of molecules containing the **right** half-cage. N is the number of particles analyzed, N_{coloc} is the number of particles containing both fluorophores, and N_{right} is the number of particles showing evidence of the **right** half-cage.

Supplementary Table 3. Calculation of enzyme copies per DNA nanocage

	N	***Cy3 Steps (% molecules)***			μ_{Cy3_Encap}	***Cy3 Steps (% molecules)***			$\mu_{Cy3\_Unencap}$	N_{enz}
		One	***Two***	***Three***		***One***	***Two***	***Three***		
HRP	176	86	13	1	1.15	92	8	0	1.08	1.0
G6pDH	218	87	10	3	1.16	93	7	0	1.07	1.1
β-Gal	284	93	6	1	1.08	88	9	3	1.15	0.9

The percentage of molecules exhibiting a given number of Cy3 photobleaching steps "*Cy3 Steps*" for both the encapsulated and unencapsulated enzymes are provided. The mean number of enzymes per cage (N_{enz}) was calculated by taking a ratio of μ_{Cy3_Encap} to $\mu_{Cy3\_Unencap}$. *N* is the total number of particles analyzed.

Supplementary Table 4. Conditions for the single-molecule enzyme activity assay

Solution	Concentration
10× TBS, pH 7.5	1×
Resazurin	50 nM
Glucose-6-phosphate (G6p)	1 mM
Phenazine Methosulfate (PMS)	12.5 μM
Mg^{2+} ($MgCl_2$)	1 mM
NAD^+	1 mM
PEG 8000	10% (w/v)

Supplementary Notes

Supplementary Note 1: Preparation, purification, and characterization of protein-DNA conjugates

Protein-DNA conjugation: As shown in Supplementary Figure 5, SPDP conjugation chemistry was used to couple enzymes to oligonucleotides as reported previously[1,2]:

a) Enzymes (GOx, HRP, G6pDH, LDH, MDH and β-Gal) were first conjugated with SPDP at enzyme-to-SPDP ratios of 1:5, 1:20, 1:3, 1:5, 1:5, and 1:5, respectively, in HEPES buffer (50 mM HEPES, pH 8.5) for 1 h at room temperature. Different values of SPDP-to-Protein ratio were used due to the varied number of accessible surface lysine residues for each protein. Excess SPDP was removed by washing with 50 mM HEPES buffer using Amicon centrifugal filters (30 kD cutoff). The SPDP coupling efficiency was evaluated by monitoring the increase in absorbance at 343 nm due to the release of pyridine-2-thione (extinction coefficient: 8080 $M^{-1}cm^{-1}$).

b) TCEP-treated thiolated DNA (/5ThioC6-/-TTTTTCCCTCCCTCC (P1), or /5ThioC6-D/-TTTTTGGCTGGCTGG (P2)) was incubated with the SPDP-modified enzymes at an enzyme-to-DNA ratio of 1:10 in 50 mM HEPES buffer (pH 7.4) for 1 h in the dark. Excess unreacted oligonucleotide was removed by ultrafiltration using Amicon 30 kD cutoff filters: washing one time with 50 mM HEPES (pH 7.4) containing 1 M NaCl and three times with 50 mM HEPES (pH 7.4). The high salt concentration in the first washing buffer helps remove DNA nonspecifically bound to the surface of the protein due to electrostatic interactions.

c) The absorbance values at 260 nm and 280 nm (A_{260} and A_{280}) were recorded to quantify the enzyme-DNA complex concentrations and the labeling ratios using a Nanodrop spectrophotometer (Thermo Scientific) (Supplementary Figure 6 and Supplementary Table 1). Extinction coefficients of DNA oligonucleotides were received from IDT-DNA, and extinction coefficients of enzymes were obtained from published data.

d) Dye labeling of DNA-conjugated proteins: The DNA-conjugated proteins were further labeled with spectrally distinct fluorescent dyes, which allow us to use native gel electrophoresis and single-molecule fluorescence to confirm the encapsulation of proteins within DNA nanocages. NHS-ester-modified dyes were reacted with the purified DNA-conjugated proteins from the above steps at a 20:1 ratio in 50 mM HEPES buffer, pH 8.5. Cy3 was directly labeled to the lysine residues on the protein surface. Excess dyes were then removed using 3-kD cutoff Amicon filters. The UV-Vis absorbance spectra of the purified dye-labeled proteins are shown in Supplementary Figure 6 and were used together with the extinction coefficients of the dye (150,000 M^{-1} cm^{-1} for Cy3 at 546 nm; 250,000 M^{-1} cm^{-1} for Alexa647 at 647 nm) and of the protein-DNA conjugates to quantify the concentration and labeling ratio of the dye-labeled proteins.

e) Conjugate proteins to Cy3-labeled DNA: In order to perform the single-molecule enzyme activity assay, selected enzymes (G6PDH and β-Gal) were conjugated to a Cy3-labeled DNA. First, NHS-ester-modified dyes were reacted with the 3'-amine of oligonucleotides at a 20:1 ratio in 50 mM HEPES buffer, pH 8.5. Excess dyes were then removed using 3-kD cutoff Amicon filters. Dye-modified oligonucleotides were then conjugated to proteins *via* the 5'-thiol using the SPDP chemistry described above. Fast Protein Liquid Chromatography (FPLC) was used to purify the protein-DNA-Cy3 conjugates for removing excess DNA-Cy3, and characterized with the UV-Vis absorbance spectra.

Enzyme-DNA cage assembly, purification and characterization:

a) The purified DNA half-cage containing capture strands was mixed with one of several enzyme-DNA conjugates at a 1:15 cage:enzyme ratio and annealed from 37°C to 4°C over 2 h in 1×TAE-Mg^{2+} buffer (containing 12.5 mM $Mg(OAc)_2$).

b) Twenty-four single-stranded DNA linkers were mixed with the two purified half-cages at a 5:1 linker:cage ratio to connect the two half-cages together by incubating at room temperature for 3 h.

c) Agarose gel electrophoresis (2%, 1×TAE-Mg^{2+}) was employed to remove excess free enzymes (70V, 2h). The band of the DNA cage containing the enzyme was cut from the gel and extracted using a Freeze 'N Squeeze column (Bio-Rad). The DNA origami concentration was quantified by measuring the absorbance at 260 nm (A_{260}) using an extinction coefficient of 0.109 $nM^{-1}cm^{-1}$.

Supplementary Note 2: Single-molecule fluorescence microscopy for characterizing DNA cage-encapsulating enzymes.

Yield estimation by TIRF colocalization: All single-molecule measurements were performed at room temperature using a total internal reflection fluorescence (TIRF) microscope on PEGylated fused silica microscope slides. To passivate the microscope slides and functionalize the surface with biotin for selective immobilization of nanocages, a biotin- and PEG-coated surface was prepared by silylation with APTES, followed by incubation with a 1:10 mixture of biotin-PEG-SVA 5k:mPEG-SVA 5k as described previously.[3] A flow channel was constructed as described elsewhere.[3] To prepare the surface for enzyme or nanocage binding, a solution of 0.2 mg/mL streptavidin in T50 buffer (50 mM Tris-HCl, pH 8.0, 50 mM NaCl, 1 mM EDTA) was injected in to the flow channel, incubated for 10 min, and the excess streptavidin was flushed out thoroughly first with T50, then with 1× TAE-Mg.

The right half of the DNA origami cage was labeled with Cy5 dye inside the cavity, *via* hybridization of Cy5-labeled DNA to complementary handles incorporated into the structure. Each of the ssDNA conjugated enzymes (HRP, GOx, G6pD, LDH, MDH and β -Gal) was covalently labeled with Cy3 as described in section 3 (Cy3-Enzyme-5'-TTTTTCCCTCCCTCC), and then linked to the left half of the DNA origami cage via hybridization with complementary handles. Because Cy3 was directly labeled onto the enzyme surface, any observed Cy3 signal of the immobilized DNA nanocages came from the encapsulated enzymes. Linker strands were added to a 1:1 mixture of the two half-cages to encapsulate the enzymes in a full-cage. To capture DNA-modified enzymes in the absence of nanocage (as control) the microscope slide was incubated with 10-20 nM biotin-modified complementary DNA oligonucleotide (5'-biotin-TTTTTGGAGGGAGGG) for 3 min, followed by 10 min incubation with 20-50 pM enzyme sample in 1×TAE-Mg buffer. Excess enzyme was flushed out with ~400 uL buffer (channel volume ≈ 30 µL). For the nanocage experiments, the samples were diluted to 20-50 pM in 1× TAE-Mg and immobilized on the streptavidin-coated PEG surface for 1 min, and the excess sample was flushed out with ~400 µL of 1× TAE-Mg. The DNA-modified enzymes were imaged with illumination at 532 nm (~15 W/cm^2), and the nanocage-encapsulated enzymes were imaged

with simultaneous illumination at both 532 nm (~15 W/cm^2) and 640 nm (~40 W/cm^2) as described.[4] Particle-finding and colocalization analysis were performed using custom-written scripts in IDL and MATLAB, using a threshold of 150 counts per frame for particle identification (typical particles showed 500-1,000 counts per frame in each detection channel). The enzyme encapsulation yield, defined as the fraction of assembled nanocages containing enzyme(s), was estimated by dividing N_{coloc} by the total number of particles containing a right half-cage, N_{right} (Supplementary Table 2).

Estimation of enzyme copy number per nanocage: The number of enzyme copies per nanocage (N_{enz}) was determined by single-molecule photobleaching (SMPB). First, the number of Cy3 photobleaching steps was determined separately for unencapsulated as well as half-cage- and full-cage-encapsulated enzymes. For this, the donor channel data of all single molecules were idealized in QuB (http://www.qub.buffalo.edu) using a six-state model[5]. The histogram of the photobleaching steps was then acquired using a custom-written MATLAB script. Representative intensity traces exhibiting one, two, and three photobleaching steps are shown in Supplementary Figure 2c (more than three photobleaching steps were rarely seen). Finally, the number of enzyme molecules per cage was estimated by dividing the mean number of Cy3 photobleaching steps of the full-cage (μ_{Cy3_Encap}) by the mean number of Cy3 photobleaching steps for the unencapsulated enzyme ($\mu_{Cy3\_Unencap}$). Results are summarized in Supplementary Table 3.

Supplementary Note 3: Single-molecule enzymology

Single-molecule enzyme activity assay: Prior to single-molecule activity measurement, streptavidin-modified slides were incubated for ~2 min with neutravidin-coated fluorescent beads (Invitrogen, 0.04 μm diameter, excitation/emission; 550/605 nm) at 10^6-fold dilution and the excess flushed out with 1×T50 buffer. These beads (~5-8 per field of view) were used as fiducial markers to correct for drift of the microscope stage and/or slide (Fig. 5a,c). Following complete photobleaching of Cy3 in a field of view, the activity of single unencapsulated or nanocage-encapsulated enzyme molecules was imaged on the same field of view. During analysis of the movies, the coordinates of the initial photobleaching movie were registered with those of subsequent movies using the fiducial markers (visible throughout all sequential movies) in a custom-written MATLAB script. This approach allowed us to infer the locations (x- and y-coordinates) of all individual enzymes/nanocages in the field of view even after bleaching Cy3, and to monitor enzyme turnovers (≈ resorufin formation) at these specific coordinates.

To image enzyme activity, 300 μL of substrate solution in 1×TBS buffer (pH 7.5, 1 mM Mg^{2+}, and 10% (w/v) PEG8000) (Supplementary Table 4) was injected into the flow channel. Movies were recorded for ~5 min (9,091 frames) at 35 ms frame exposure time immediately after injecting the substrate solution. In case of G6pDH, the activity was measured in the same field of view under identical laser illumination and microscope settings, with or without glucose-6-phosphate (G6p) (Fig. 5c). Enzyme activity for β-Gal was measured similarly using a 500 nM solution of resorufin β-D-galactopyranoside (RBG) as substrate, which is hydrolyzed by β-Gal into fluorescent resorufin. Fluorescence fluctuations over time were measured for unencapsulated enzyme as well as half- and full-cage-encapsulated enzyme (Supplementary

Figures 57 and 58), and the fluorescence time traces were analyzed for intensity spikes using custom-written MATLAB script. The script allowed us to measure the background intensity of single-molecule traces and set a threshold (mean + 8 standard deviations) to subtract from the raw intensity. Since we often observed one or two spikes above this intensity threshold in the control experiments, only those molecules with ≥4 spikes were counted as active molecules (Supplementary Figure 59) and considered for burst analysis. Due to the low concentration of resazurin (Supplementary Table 4), the criteria we used to determine the fraction of active molecules might have excluded some molecules that are not highly active.

Burst analysis: Burst analysis was carried out using a modified Rank Surprise (RS) method[6] recently utilized to analyze the binding of fluorescent DNA probes to a riboswitch[7]. Briefly, Interspike Intervals (ISIs) were determined by calculating the time in between individual fluorescent spikes for each molecule (Supplementary Figure 59). The RS method was used to demarcate the start and end points of bursts after collecting ISIs for all molecules. Only intensity spikes characterized by an ISI of ≤5 seconds were considered part of a burst; any other intensity spikes are counted as non-bursts.

Comparing bulk and single-molecule enzyme activity: Unlike our single-molecule assay, the bulk measurement of enzyme activity cannot explicitly determine the fraction of active enzyme molecules present in the solution (it is well known that a fraction of enzyme molecules loses their activity during oligonucleotide conjugation, buffer exchange and the purification process). However, the observed bulk activity is contributed not only by enzyme turnover rate but also by the fraction of enzyme molecules that are still active. Both of these contributing factors need to be accounted for to directly compare the single-molecule enzyme activity with the bulk measurements. Therefore, in the single-molecule experiment, the overall activity of free, half-cage and full-cage enzymes were calculated by multiplying the turnover rate with the fraction of active molecules for the given sample.

Supplementary Note 4: DNA sequences of the designed nanocages.

Sequences of staple strands in the SH Full-Cage-Left cage

5[18]	GGTGGAGAGGCGGTTTGCGTTTT
11[18]	CGAGTTGGGTAACGCCAGGTTTT
13[9]	TTTTTCGCCATTCAGG
17[9]	TTTTGCCAGCTTTCATCAACATTCGT
21[9]	TTTTTGGAGCAAACAAGAGAATCGGAAGATTAGC
25[9]	TTTTGGGAGAAGCCTTTATTTCAAAAAGGGACAG
31[5]	GGTGGCATCAATTCATGGGCGCGACCTGTTTGTATAAGCAAATTTT
36[16]	ATATAAAGTAGTAGATGGGCGCTTTT
43[18]	AATCATACTAATAGTAGTAGCATTTT
54[17]	GCTGTCATAGCACCGAGCTCGAATTCGTTTT
55[2]	TTTTTGAGGACTAAAGACTTTCAACACTAAGG
67[18]	CGGTTTTGCTTTGCGCTAGTGAGCTAACTCACATTTT
69[2]	TTTTGAAGGATTAGGATTAGCGGTAGCAACGCGA
83[2]	TTTTAAAAGGGCGACATTCAACCAGGC

95[18]	TGACTAATATGTTTGATGTTTGCCCCAGCAGGCTTTT
97[2]	TTTTAGGCTTATCCGGTATTCTAGTTT
108[20]	CTCAACAAGTATCACATAATTTATTAAAGTTCCAGTTTGGAACATTTT
1[5]	TTTTAGAGTCCACACTAGAAAATT
3[5]	TTTTGAAAATCCTCAGAGAGATTTT
5[5]	TTTTATTGGGCGGAGCCACCATTTT
7[5]	TTTTAATTGCGAAACAACTTTT
9[5]	TTTTAATCATGGCTCATTCAGTTTT
11[5]	TTTTGTTTTCCCAGTCATTTTT
15[9]	TTTTATCGTAACCGTGGCAAAGCGCCATTTT
19[9]	TTTTATTTAAATTGTGGCCTTCCTGTATTTT
23[9]	TTTTGAGACAGTCAAATGCCTGAGAGTCTTTT
27[2]	TTTTAGCCTCAGAGCATAAAGCTTAATACTTTTGCTTTT
29[2]	TTTTAACATCCAATATTAAGCAATAATTTT
33[2]	TTTTAAATGGTCAATAAGCTGAAAATTTT
35[5]	TTTTATTCCCAATGATACATTTCGCTTTT
37[2]	TTTTAAATATGCAACTAACAGTTGTTTT
39[5]	TTTTGCGGATGGCCTCAACATGTTTT
41[2]	TTTTGTTTACCAGACGACGATAATAGCAAAAAATCATTGAGAAAGGCCGTTTT
43[2]	TTTTACATAACGCCAAATCATAACCCTCTTTT
45[5]	TTTTAGAAAGATACTAATGCAGATTTT
47[2]	TTTTGGAAGAAAAATCTATTACAGGTTTT
49[5]	TTTTGAATTACCTGTCAGGACGTTGTTTT
53[5]	TTTTGAATAAGGTAAATTGGGCTTTT
57[2]	TTTTCACCCTCAGCAGGCTACAGAGGCTTTT
59[5]	TTTTATATTCGGTTTGCGGGATCGTTTT
61[2]	TTTTGATACCGATAGTCATAACCGATTTT
63[5]	TTTTAATTGTACTTAAACAGCTTTT
65[2]	TTTTAATAATTTTTTAAGGAGCCTTTT
67[5]	TTTTCAACAGTTAGGAATTGCGAATT
71[2]	TTTTCGGAACCTATGACTCCTCAAGATTTT
73[5]	TTTTGTCAGTGCCCCCCCTGCCTATTTT
75[2]	TTTTCATACATGGCTTTTAACGGGTTTT
77[5]	TTTTCATTAAAGCTTCCAGTAAGCGTTTT
79[2]	TTTTAGGTTGAGGCAGATAAATCCTTTT
81[5]	TTTTCCCTCAGAGAGCATTGACAGGTTTT
85[2]	TTAAGTTTATTTTGAGCGCCAAAGACTTTT
87[5]	TTTTATACATACAACACCACGGAATTTT
89[2]	TTTTGAACTGGCATGAACGTAGAAATTT
91[5]	TTTTCAAAGTTACGAATACCCAAAATTTT
93[2]	TTTTGCAATAGCTATCATAGCCGAATT
95[5]	TTTTAACCCACAAAACAATGAAATATTTT
99[2]	TTTTCGAGAACAAGCAATCAGATATAGATTTT

101[5]	TTTTAGAAACCAATACCGCACTCATTTTT
103[2]	TTTTACGCGCCTGTTTCGAGCATGTTTTT
105[5]	TTTTATAAAGTACAGCTAATGCAGATTTT
107[2]	TTTTTGAGAATCGCCATAAGAGAATTTTT
109[5]	TTTTAGCCTGTTTGTAGGGCTTAATTTTT
1[28]	GACTCCAATAAACACCAGGGAAGCGCATAAGTCAGCGGCAAATGCAGCA
1[77]	GAACCATCGTAAAGCACTAAACTTGACG
2[34]	TAGGGTTCCGAAATAGGGTAAACAAATC
2[55]	GGTCAAAAGAATAGAGGGCGAAAAACCGTAAATAAGAGAATTAA
3[49]	ATAAAAGGAACACCCACCACCGG
3[77]	GGGAAAGGGGCGCTGGCAAGTCGCTGCGCGTAACCTTGACGA
4[34]	AGCGGTCCTTTTCACCCTCAGATTTAGC
4[69]	GTAGCTCTTTAGAGTCGGAACATGGCCCACTACGT
6[34]	CGGCCAACGCTTTCTTTTCTGAATGGCT
6[48]	CTGCATTGACGGGCAGAGAGTATCCCTT
6[76]	GCACGTAAGCTAAACAGGAGGTTTTATAATCAGTGGTAAAAG
7[25]	GCCCGCGCGGGGTTTTTCACGCTGGGTGGTTGAGTGTTGAACGTG
7[49]	GTCGTTTTTCCAGAGTAATCTTG
8[34]	TGGGGTGTGTGTGACAAATCACTCGAAC
8[69]	AGGCCATGAGCGGGTAACGTGCTGGTCA
9[77]	AGTCTGTCACTTGCCTGAGTAATCCAGAACAATATACGCTCA
9[88]	CACGAAGTGTCCGATTAGGTTGCTACCACACGGCG
10[34]	GAGGATCAAACGACAATTGCTCAGTTTGTAGGTCAAAATGTGAATAATT
10[48]	GCAGGTCATCCGCTTAAAGTGGAAACCT
11[49]	TTGCACGTCAGGATGTATCGGGGCGGATCGTCGGAACCAATA
12[34]	TGCAAGGACTGTTGGTGCCGGAAACCAGCATCTGCCCTTTTGTTAA
12[69]	GAAATATTGGTAATGAAGAACACACCGA
12[76]	ATCGTCTATTTACACAGAGATAGCGCAC
13[21]	CTGCGCACGATTAACGTTGTACCCGGGTTGTTTCCCCTAATGCACT
13[42]	CGATCGGAAAGGGGGCCAAGC
13[84]	ATTCACCCATTTTGTACCGCCATAACATCCAT
14[48]	CGGCACCGACGACATAGA
15[63]	AAGATAACCCTTCTAGCCCTAATTAAAAACGCTGAGAGCTCA
15[91]	AGAATACGTCTTTAACCAGCAAACACCGCCTGCAAAAATCTA
16[24]	TGGTCGGTGTCATATAATAGGACAGACCAGAAAAAATCTAAAG
16[69]	AAACAGGAACAAACCCTCAGGTCCAGCCCTCTTCGCTGGATT
17[42]	AACAACCTGACCGTGACTTCAAATAT
17[84]	ACGAACCATGCGCGAACTGATGACCTGATGGCCAATTGGCAG
18[27]	CGCGTCTAAACGTTAGTTTCAACGAGTAAACTTTGTTTT
19[63]	AATCAGCCAGCAGCAATCAACAATTGAGGATTTAGAAGGAGA
19[91]	AAGCATCTCAATATATATCTTTCAATAGATAATACACAATTC
20[69]	AGTTGTGTACCCCGTTTGTTATTTTTTATTCTCCGTGTCGCC
21[42]	GTAAAACATCAGAACAGAAAACGAGA

21[84]	ATCTAAACTGGTCAGTTGGCAAAATGAACAGTGCCATACCGA
22[27]	AGGTCATTCACCATTCATTTGACTGCGGTAACGGATTGA
22[48]	GAGAGATACCGTTCTAGCTGATGCCTGAGAACCCTTGGAAGG
23[63]	ATGCCGTATTAGACATCATTT
23[91]	GACAACTTTTAAAAAATTATCCATCAATATAATCCTGATTGTACCAGAA
24[69]	TGCGGTAAATGCAATAAATTAGGGTAGCTCAATCATAAAAGG
25[84]	GGAGCGGGTTTGAGTAACATTTTTACAAATTTGAGGAAGGTT
26[27]	GACCCTGAAATCGGAAAGAATAAACCAAGTAAGAGTTCA
26[41]	CCTACAAGATAAAAATTTTTAGTAATGTTTTGCCAGAGGG
26[48]	GTTAGAACAAAATTGTAGATTTTCAGAGGCTTTGCTTGTACCAACATAT
26[62]	TGAATAACATATATTTAACAAAGAAACCTTGGATTATACTTC
29[25]	GCATAGCGTCTACGAGGGAATACCACAGCATAGTTAAAAAAC
29[35]	AAGAAGTGTAGGTAATATTCACTACAAAGGTAATC
30[53]	GGTAATAGTAAACCAACCTAAAAC
32[46]	TGAATCCCCCTCAAATGTTCAATATGAAGATTCACGCAAGACATTAT
33[25]	TATTAGTTTGATAAAACGGCTCATACAATGATTCGAGGATAC
34[34]	CAGGTTTATCTTTAAACAGTTAAGCCCCTGTTAAACATCAAAAGCGAGT
35[35]	CCCTGACAAAAGATTAAGAGAAATATTTAAAAACAGATGAACGGCTATC
36[57]	AAAGCGGATTTAAATTGTTGATATAGCATGTATTTTT
37[35]	CCCGAAAAATGGGAAGGGGACGCTTCTGGGAAGGG
38[53]	CGCGTTTTAATTGA
39[25]	TTGACGTAACTGACGAGTATGGGAAGTGAGAAACCGCCCAGA
39[35]	CAGACCGTACCTTTGGCCAGTGATGTGC
40[44]	GAGGAAGCAAGGATATTATCAAGACGTTAGTTCTAAAGCCTC
40[57]	GGGCCTTGCTACGCCAGCTGGCGTGCGGGCAGCTTTC
43[35]	GAAGGCAATGTTTAATAAATATTCAT
44[53]	GAAAGAGGCAAAAGAGGGTTGATA
45[25]	GATAACCATCGGCTTGCTACTGGTACAGTGCCAGTATGGGCA
45[35]	TCATCTTAAGTACAACGGAACAATCGTCGACTGGAAGTGCAA
46[57]	TACCAAGCGCGAAACATGACCCCCAGCGATTA
48[16]	CCATATGCGAAAACTTTAATCATTGT
49[25]	AAGAAAAAAGATCAGCTATATTCAGAAAGCGAGAAAAGAAAC
49[35]	TACTTAGGAACCGAGTGTACACGAGCTTCAAAGGATGGGAAG
50[57]	ACGGTCAATGTCAGAAGC
51[2]	GAGATGGTTTAATTTCAAGGCTGTAGTTAGAGCATAAGAGGTCA
52[34]	TAGGCACATGAACGACTGACCGACTTTA
52[43]	CCACAACGCCTGTA
53[35]	GACCTTCCATTACCAATTGTTGACTCTA
54[57]	ACCCACACATCAAACTATTGCCT
55[25]	TGAACGAGGGGGTTTTGTATTAAGGATTGAGTCATATGAGAACGCCCAA
55[35]	TCCATTAATACGTAATGCCTAAATAGCGAGGTTTAACGTCAGGGGTAAA
55[42]	AACGGAGTACCAAGTTACAAGGCGGAGAGGAAGTT
57[18]	AAGACATTCATCATCAGACAACATTACGTTAACCATTATCTGCGATTCC

57[35]	GCCGTCGAGAATACACTAAAGCAACTAC
58[53]	TAAGTATAGCCCCACCGTCACCGA
60[46]	TTTAGTACCGCCACCCTACTTAACAC
61[35]	CCGCCACCGCGACCTGCTCCTGAGATTTGTATCATCAAAAAT
62[16]	TTTTCGGTTTGCTCCAACACGTTGCGAGTAGCTTGCCCAAA
63[35]	GGGATAGTGAGTTTCGTCAAAAACATGT
66[23]	GAAGATTGGCCCAGAGCAGCCCTTTAATAAGCAACGCCGCCAACG
66[53]	GCATTCCACAGACA
67[35]	GTAACGAAAATGAACAGTCGGTAAAGCC
68[57]	CTAGAATCAACGAGCCGGAAGCACACAATTAAGAACCACTCCAACA
70[16]	TGATATTCTGGGCCGCTTCGCTGAGCCCACGTGCGCCGTATA
70[34]	CATTAAGCTCAGTACCAAATCGCGCAGAAGACGGA
71[25]	ATGATAAACACAATAGAAAAGAAATTTATTTGGTATTA
72[34]	AGAGCGTAAAAGGTGAATTATGGAATAGGTGTAGGCGTAAGT
72[53]	CTTGAGCCATTTCGGGAGGTTTTG
74[34]	CAATGTGAGTCACCGTACTCAGGAGG
74[57]	ATTAGCAAGGCCGGAACAGTAGCACCATTACC
75[25]	AGGCTCTGAATCCTTATACGCAATATAGATATAAACAA
76[34]	GTTTGGTAGAAACCATCGATACACCACCCTCATCTCACAGAA
76[53]	TCAGTAGCGACAACGAGCGTCTTT
79[35]	CGTTTGCCAGCCCTCATAGAGCCCCAGTACAAACTAGGCGCA
80[43]	TCAAATAGCAGCCT
81[25]	ACCTTGAGCGGTTAAGCCCGGAATTATGCGTTATACAA
81[35]	ACCGGAAGCCGCCACCAGTGAAATGAAT
82[44]	AGACCAGAGCCTGAACATAGACGGGGCGTTATGACCTA
82[57]	AAGATTGCCCTTTCCTCGGCCAG
83[42]	TATTGAAAATTACATTTAATAGCGAAATGGAGGGAAGGTAAAAATTATT
84[16]	ACCTCACAATGTTAATGTTGAGTAAATAAGTTTTGATGTGAA
86[53]	AAGCCTTAAATCGAGTGAATAATTTTCCATTCC
87[25]	ACACCCATCCTCGGCTGTCTTTCCTTATCCTAAGAAAA
88[46]	CAATTTTATCCTGAATCCGCCCAGCAAAATCACACGTCAC
89[18]	GACTTTACCGCAGAATGCAAACAAGTCAGACCAACTAATCAG
90[53]	CCAGAGCCTAATGTGAATTTTAACCTCCAGACGACGACAAAGTCCTG
91[18]	AGGTAAGCAGTTACCGACGCCGCCCCGCCACACCCTCACCAG
91[25]	CGATTTCGAGAGGTAAAGTAATTCTGTCCGGAGAGGCA
92[34]	TTTAATACACCTTTAGCGTCACATAGCCCCCTTTGTGTTTCA
92[57]	TCCAAATAAGAAACGAATATTATTTATCCCAA
94[34]	AAAACAATTCGTCAAAAATGATTTTCATAATCACACTATTAG
94[53]	TTACAGAGAGAAAAAGAACATTTCAT
96[57]	CTCCCCCGAACCGCCTGGCCCTGAACAGCTCCGCCTCTTTTGTCGT
97[42]	TCATTTGTCAATATATTCATT
98[23]	TAGCAAGCAAAGCCGTTCGCAAAGTAAAGGTTTAGCAATTAA
99[35]	AGGAAGTAAGATTAGTTGCTAAACCTCCCGACTTGGGGAATT

100[20]		AACCAAGTCAATAATAATTTAATCAACAAATAACGCAGA
100[34]		AAGAACGTCATCGTACCGCGCGAGGCGTTTCAATT
102[34]		ATAATATTATATTTTGCACCCAGCTA
103[35]		AACAATATTGCCAGTTACAAATATTACCAACGCTAGAATCAA
104[20]		CATGTTCCGACAAACCAGTAATATTTAAAGCAAGAGAAT
106[34]		GAGGCATGGAAATAAACAGCCTTTTTTG
108[41]		CTTACCAGTATAAAAACATGTAATTTACTAACATA
109[35]		ATTCTTCAATAAGAACGTCAACCCGAGA

4[86]		CTACCGGCGAGAGGTGCCACCCAAATCAAGTTTT
12[107]		TTTTGCTCATGGAAATACCTAAGTCACATAAAAGGGACATTCAAGCGTA
91[46]		TATAATCGCACTTAGGTTGGGTTATACCTTTTATCAAAATCATAGTTTT
101[49]		CTTGAAATATTAATTAACCTTGCTTCTGTTTT
102[65]		TTTTTAGATTAAGACGCTGAGAAGAGTCTAGAATC
106[65]		TTTTAATGCTGATGCAAATCCTTATCCCAA
109[49]		AATTTAATTAGTTAGCGAGAAAACTTTT
110[67]		TTTTCCGACCGTGTGATCTATCACCTAAAG
2[96]		TTTTGGGGTCACGTGGCGAGAAAGGAATTTT
4[107]		TTTTAGAAAGCGAAAGGAGCGCCGCCGCGCTTAATGCGTTTT
6[107]		TTTTTACAGGGCGCGTACTATAAGGGATTTTAGACAGGTTTT
8[107]		TTTTGTACGCCAGAATCCTGAGCAAATTAACCGTTGTATTTT
10[107]		TTTTTACTTCTTTGATTAGTAAGCCATTGCAACAGGAAATTTT
42[62]		TTTTTACCTTTTACATCGATGAATATACAGTATTTT
70[62]		TTTTATTACCTGAGCAAAGGCGAATTATTCATTTTT
98[62]		TTTTAAACAGTACATAAAAATTACCTTTTTTAATTTTT

Sequences of staple strands in the SH-right cage

14[176]		TTTTATAACATCACAATATTACTTTT
16[176]		TTTTCGCCAGCCATTGCAACTCCAGAACTTGCCTACTTCTTTGATTAGTATTTT
18[176]		TTTTATCGTCTGAGGACATTCTTTTT
20[176]		TTTTGGCCAACAGAGATAGAATAAAAGAATGGATTACATTTTGACGCTCATTTT
22[176]		TTTTAATATTTTTTAAAAATACTTTT
24[176]		TTTTCGAACGAACCACCAGCTCGCCATGAATGGCAATACGTGGCACAGACTTTT
26[176]		TTTTCCTGCAACAGTGCCACGTCAGTATTAACACCGTTTT
28[169]		TTTTACCTCAAATCAAATCAACTTTT
30[169]		TTTTAGTTGAAAGGAGCACTAACAATTTT
32[172]		TTTTCTAATAGATTAGGAAGTATTATTTT
34[169]		TTTTGACTTTACACCGAACGTTATTTT
36[172]		TTTTAATTTTAAAAGTAACCACCAGTTTT
38[169]		TTTTAAGGAGCGGGGCAATTCATCATTTT
40[172]		TTTTATATAATCCTGATGTTTTTATAATTTT
42[169]		TTTTAAACATAGCATAGTGAATTTT
44[169]		TTTTATCAAAATCATATTAGAGTCAGATAGCTCCCTTAGAATCCTTGATTTT

46[172]	TTTTAACCTCCGGCTGATGCATTTT
48[169]	TTTTAATCCAATCATATATTTTAGTTTT
50[172]	TTTTTAATTTCATCTTCGTGTGATATTTT
52[169]	TTTTAATAAGGCGCTAGAAAAAGCCTTTT
54[172]	TTTTGTTTAGTATCAGAGCGGGAGCTATTTT
56[169]	TTTTGGAATCATTAAGGCTTATTTT
58[169]	TTTTCCGGTATTCACTTGCGGGAGGTTTT
60[172]	TTTTGAAGCCTTTACAATTTT
62[169]	TTTTATCCTGAATCTAATTTGCCAGTTTT
64[172]	TTTTACAAAATAAAAACGATTTT
66[169]	TTTTGTTTAACGAATAACATAAATTTT
68[172]	TTTTAACAGGGAAGCGGCGCCGCTACAGTTTT
70[169]	TTTTGACATTCAAAAATTATTCTTTT
72[169]	TTTTATTAAAGGTGGAATTAGAGCCTTTT
74[172]	TTTTAGCAAAATCACCCGTCACCAATTTT
76[169]	TTTTGAAACCATTCAAGTTTGCCTTTT
78[172]	TTTTAGCGTCAGACAGCCCCCTTTT
80[169]	TTTTATTAGCGTTCAGAGCCACCACTTTT
82[172]	TTTTCGGAACCGCCTCAGCGGGCGCTAGTTTT
84[169]	TTTTAAGAGAAGGGCGGATAAGTTTT
86[169]	TTTTGCCGTCGAGTATCACCGTACTTTT
88[172]	TTTTCAGGAGGTTTAACCGCCACCTTTT
90[169]	TTTTCTCAGAGCCTAGGAACCCATGTTTT
92[172]	TTTTACCGTAACACTGTAGCATTCTTTT
94[169]	TTTTCACAGACAGGTCGTCTTTCCATTTT
96[172]	TTTTGACGTTAGTAAAGCCCCCGATTTATTTT
98[169]	TTTTAAAATACGCAGCGATTATTTT
100[169]	TTTTACCAAGCGGACGGTCAATCATTTT
102[172]	TTTTAAGGGAACCGAGTAATCTTGTTTT
104[169]	TTTTACAAGAACCCTTGAGATGGTTTT
106[172]	TTTTAATTTCAACTTCTACGTTAATTTT
108[169]	TTTTAAAACGAAGATACATAACGCTTTT
110[172]	TTTTCAAAAGGAATTAGAACCATCACCCTTTT
1[154]	ACTACGTCGAGGCAAAGTTTTCCCTCATAACGCCTGAGTTTCGACA
2[132]	AGTGTTGAGGGCGAAAAACCGCTATCATTGAGAAT
3[133]	GATAGACTGCTAAAGCCGCCACCAGATCCCCTCAGGGAAGGGTGCGCGT
5[158]	AGGCCTCAGAACAGAGAGTCAAAAAATAAGACAGCCATTTTT
6[139]	AAGAGTTGCAGCAAAATCCTGTTTGAAAAACCGCCAGCGCTA
7[133]	TGAGACCGAACACCTTAATTGAGAATACATTCTTAGTGCTTTAGACAGG
7[151]	CGCGTCACGCAAGAAAGGGCGAACGAACCCTCGAGGTGATGGCCC
7[158]	AATCATTAGAATAATTATTAAATATACCGACCTGA
10[139]	AAAATCGGCCAACGAGGGTGGTTTTTACCCAGTATAATTATT
10[160]	ATTAAAGTGAGAAGTTGTTTGGGTAATAAGGAAAAAAATACCTATTTAC

11[133]	TAATGCGATAATGGCAATTCCAATCATGCCCCGGGCGGCCAG
13[147]	ACCGTTGAAGAGTCAGAATCCGGATTTTCCTCGTTTTGACGACCGC
14[132]	GAGCCGGCCGCTCAAAGGGTTAGAAC
14[153]	AGAACTCAAACTACCAAATTA
15[147]	CCTTGCTGATTATAGATTATCTATACAACGGA
16[153]	ACGCTCGTTGCGGAATCA
18[132]	TGCCAAGCACGACGAGATGAATATAC
18[153]	ATTGGCATCACACGACATTATATTAAATAAATTTAGAAAACTATTA
20[153]	TGACCATCATTTGACGACAACAACTATAAAAGAACATTTTGCACGC
21[158]	AAGTATTAGTCTTTAATATAGCCCAATAGATTAAA
22[132]	GGAAGGGCGCCATTTCATTTCAATTA
22[146]	GCGCGCAGAAAGGGGGTGAAA
24[128]	GTGTTTCATTAAACAAAATTCCAACAATAATCATACATAGTATGTAGTT
24[153]	AAAACGCATCTGGTGGAAGGTGCTGAGATACGAGCCAAATCAGCGA
25[158]	GCGGCTGAGAGCCAGCAAATCTAACCTC
26[132]	CCAGTTTTGGGCGCAGTACATCTGTAAACAAATTG
26[146]	GCAAAGTCTCCAGCCAAGAGG
27[67]	ATTCTCCGTGGGAACAA
27[84]	ACGGCGGTAAATGTAAATAATTTTTGTTAATCAGAGGTA
27[105]	GATAGGTGAAGCCAGCTTTCATCAACATATTGACCGTAATGG
28[149]	AATTAATTTTTAGATTAAAGCCGTCCAA
30[139]	ATCAAGATGAATTACCTTTATTTTCCGGCGAACTG
31[121]	CCTGAGCAAAAGAA
31[140]	TGAAATCAAGAGGCGAATTATCAGGCTGCACCGCTGATCGCAGCATCTG
31[151]	ATATTGGGTTCCATCCTGATTAGTTAGC
33[140]	GCGCATAGTGCTGCACACCAGGATTCGATACCGAGCTCATGG
35[121]	AGTAACAGTACCAAAGTACCGACA
35[140]	TCGGGGTCAAGGTTTAACGTCTTGTAAAAAGGCGAAGCTGGCACTGTTG
37[128]	AAATTGCGTAGATTTTCTTAATTCGTACATCGG
38[149]	TATTTTGAAAAGAATAACAATCCAATGAAAAGCAT
39[121]	CTACCATATCAAAAAGCCAACGCT
39[140]	TGCACCTCTTCTGAGTACGCCTGTCCATCATTGCGCTCACTGGCTGCAT
41[99]	GGCCTTCCTGTGTTTGTTAAAGGAAGAGTAACAAGAGCATT
42[125]	TAAACGTCCTTATCATTAATTAC
42[139]	AGTACCGTCGTCGCTATTAATCATTTAATGGAAACATCGTAACCTGAAA
45[131]	CATAACGGAATACC
47[117]	ATGCAGAACCCTGATTGC
47[140]	ACAATATTCAGAAACAATAACCAAAATC
49[158]	CAAGCAAGACTGTAAATGCTTAGGTCTTTAGGAATTGACAGTTGGATCA
50[156]	CCTCCTTTGCAACAATTGGATTTAAGCCGTCTAAAACAAGAAGATTGAG
52[139]	TATTTAATTCGAGCCAGTAGGTCGTAAAACAGAAATAAAG
53[121]	CAACAGTAGGGCCTGAACAAAGTC
53[151]	ATCCGGGAGAAGCCTTTGCCGCCAAAAATCATCTG

54[156]	CGTAGATGATAATTATCACAAAGATTGAGTAACCAGTAACCCTTCGCGT
55[126]	ATTTACTCGCAAAGAATAGAATTACCAT
56[86]	ATCAGGTCATATGCCGGGTAGGTATTTTTAGAATACTTGAGCATA
56[97]	GATGAACAAAGCCCCAAAAACAATTCGCATTAAATTCGCGTCT
56[149]	TAGACCAGCGAGGGAGGGTATTAATTAGCGGTGAGGAAACTACGAA
57[117]	TAGGAATAATTGTATAAGCAAAT
57[140]	GCAACAGATGTAGATAATATCATAGATAAGTCCTGAAGATGA
58[149]	GGCGTAAATACACCGTCTTGCTCAGATATAATCATCTTAAGTACA
59[121]	CAAAAGAACTGGTG
59[151]	GAACCATTACACTTGAGCACCCTCAGCCCGGAACTTTGCGGAACGA
60[139]	GCAATAAAGAAAAAAACCAATGAACGGGTATTAACTACAAAC
62[139]	TTTAAGAAAGGAAACCGAGCTGCCGACGACAATAATTTATCA
62[149]	TAAAGCAAGGAGCACCGCCGCCACTCATTTTGACCTTCCATTACC
63[121]	ATAGCAATAGCTCACAAACAAATA
64[139]	AAGAGCAATTCTGTCCAGAGAATATAAGAGAATATTTTTACA
64[149]	TTTCGTCTTTCGTTTTCCAGTAGCGTCACCAGATA
65[128]	GAATTGAGTTAAGCCCACCACCGCCA
67[121]	AGAGGGTAATTGACCCTCAGAGCC
67[140]	ATATCGCATTAACTACCACACGCACGTATACTTTTCACCAGTCTGGCCC
69[140]	ATGGTTTCAATATAAAAGAAACATCGAGAACAAGCAGAACCA
70[111]	ACCGTTCTGAGAAACATTCAACGCAAGGATAAAAAAAGATTCA
70[125]	CAATATGTATAAACTAGCAAACG
71[117]	CGATATTCAATTTTGTCACAATCACACCACGAAAATACGGCTGTCT
71[130]	AATCTTGAGTTTTGCGGGGCTTGCCAAAAGACATCGCC
72[139]	TCAGTGCGCCCCCTGCCTAAGGTTCCTTATTACGCAAAGGTG
73[131]	GTAATGACAACAAC
73[140]	TTTAACGCAACAGGAGTGTACCATGATTAAGACTTTGGAAAC
77[121]	AATCCTCATTAATCTCCAAAAAAA
77[140]	ATGGAATATGGCCTTGATATTATCTTACCGAAGAGATATAAT
77[158]	GAACGATAGCCCGGAAAAGTAGCACCTCCCGTAAGAACGATATAGACCG
79[140]	CGATTCGTAAGAGAGATAACCCACAA
79[151]	TTTTTTTCATAAACTACAGTTAGCTTGGGAAAACAACA
81[121]	ACCACCCTCAGACAACTTTCAACA
81[158]	AACTGCCATCCGGTCATTGTAGCGCCAGAGCCTTACCAACCCAGCAAAT
83[99]	AAAGGGTAGCTGATAAATTATGCCTGAGAGTCTGGAGAATC
83[126]	AAATCATACAGCATTGAGGACAACGAAA
84[139]	GCGAAAGTCTGAAACATGAAAGATTTCGGAACCTAAATTCAT
85[117]	AAAGGGTAGGGCCGGAGACAGTC
87[121]	CATCGCCCACGCAACGGTGACCTGCT
87[140]	ATATAATAGAGTTGCGCCGACAATAAGT
89[117]	TGAATTTCTTAAACAGCAGCTTGGACCAGGAAAGCTG
89[140]	CCGATAATAAAGCGCAGTCTCGCTTTTGATGATTTCGCCCTT
90[156]	CCCCCTCAGAGTACCGCCCATTTGGAATTATTTGACGGCCGA

91[121]	AGGCTCCAAAAGTTAAGAACTGACGA
91[140]	TAATTGGGTTCACGTTGAAAAAGCCAGA
91[151]	GCATGTGAATAGTAGTAA
93[128]	TGCGAATAATAATTTTTACAAACCACCACCAGAGGTCAGA
95[121]	GTTTCAGCGGAGAACCCTCGTTGAGA
95[140]	AGAAAAAGGGATTTTAAATCGGTGGCGAGATGGTGGTTCCGAAGCCCGA
96[149]	TATCGATCTATAGTAAGATTCAAC
97[98]	AGAAGCCTTTATACAAATTAAGCAATAACATCCAATAAATCAAATAACC
97[119]	GAGGCTTCGGAACGGGCCGCTAACAGTGCC
98[125]	GAAAAGAATTAGCAGGCTACA
98[145]	GGCACCAAAACACGTTTCGGTCGCTGAGATCGTCACCCTTTACCGGGG
100[104]	TGTTTAGTACATTTAAGTTTCGTAGCTCAACATGTAGAGAGT
100[118]	TTGTGTCTACAGGCAAGGCGGAGGGAGTTA
100[125]	TGATAAAATCCGCGTAACTAAAGTACGGTGTCTGGCGCAAATGGTCGAA
100[146]	ACGGAGAACTTAGCAAAGAGGGCTGGCTCAGTATCGGTTTATCTTGATA
101[133]	CCATGTTTTTGTATATACACTAACCTAATAAAGACTTTTTCAGGCAGCA
102[90]	ATAACAGGTTTGACCATTAGACTATATTGCATTAAAGCCTCATTGCGGG
104[104]	ACCTTTACTCCAACGAAGCCCTATTATAGTCAGAACATTGAA
104[125]	CTCATTCAGTGATTTTTAAATATGCACACTTTCGAGG
104[146]	CAAATCACAGAACGTACCTTACAGGACGGTGGAACAACTAAAGGAAT
105[133]	GAAACACACGTAACCGCATAGACAGATGATAACCG
105[155]	ATTGGGGGATATTATCAAGAACTGACCAATAGGTGAGGGTTGTAC
106[90]	TTCAAATAGACCGGAAGCAAAATTGCTCTAATGCTATTCCAT
106[111]	TTAAGAGAGGTCAGGAATAAGGCTTGCCCTGCATC
107[119]	GGATTGGCTCATTAAAGATTCATGTCATAAATATTGCAAAGCAAAAAGA
108[104]	TCCCCCTTGGATAGACCAAAATAGCGAG
108[146]	TTATTACATACCACAGCAACATCTATCACCGTAAAGCGGTTG
109[133]	TTTAGGAAGGTAGATACCAGTTGCGATTGAGCCTT
109[154]	TAATGCACTAACGGGAAAAATTAATCATAGCCCAAACCA
110[90]	AGGCTTTTAAAATGTTTAGACCAAATGCCCCTGACGAAAGAC
110[118]	AGACGACGATAAAACGTCCAATACTGCGGAATCCAGTTTACC

1[105]	TTTTGAACGTGGACTCCAACGTCAATTCCAGTTTGGAACAAGAGTCTTTT
15[116]	TTTTAAATTGTTATAAGCATAAAGTGTAAAGCCTTTT
17[116]	TTTTGGTCGACTCTAGAGGATGTCATAGCTGTTTCCTGTTTT
19[116]	TTTTCCCAGTCTTGCATGCCTTTT
23[116]	TTTTAAGCGCCATTCGATCGGTGCTTTT
41[80]	TTTTCAAGAGCGAGTAACAACCCGTTTT
55[67]	TTTTATGTACCCCGGTTGATAAATTTT
69[81]	TTTTATCGTAAAACTAGCATGTCATTTT
83[67]	TTTTCAATGCCTGAGTAATGTAGATTTT
97[65]	TTTTAAAAACATTATGACCCTGTAACCCTCATATATTTTATTTT
109[70]	TTTTAGAGGGGGTAATAGTGCAAAAGAAGTTTTGTTTT

13[106]	TTTTCCTAATGAGTGAGCTAACTCACATTAATTGCGCGAACATAC
21[116]	TTTTCTCTTCGCTATTACGCCTTAAGTTCAA
25[116]	TTTTAGTATCGGCCTCAGGAATCTGTTTT
29[117]	ATTATATGTCACGTTGGTGTAGAGAGGGGACGATTTT
33[117]	TTTGAATACGGGTAACGCCTTTT
43[117]	TTTAATATTGAATAACCTTGCTTAAATCAATTAACAACCGGAAACCA
98[83]	AAGCTAATACTAATAGTAGTATTCATTTGGGGCGCGAGCTGAAAATTTT
101[70]	TTTTAACGAGTAGATTTATTGATTCTTAATTG
103[84]	CTGAATACTTTTGATAAGAGGTCATTTTTGCTTTT
105[70]	TTTTCTTCAAAGCGAACCATCGCGTAAATCAG
107[84]	GTCTTTATTTAAACAGTTCAGAAAACGAGAATTTT

2[172]	TTTTAAATCAAGTTTTTTGGGGTAAAGGGATGAATTTCCGG
4[172]	TTTTGAGCTTGACGGGGAAAGCCCGAA
6[172]	TTTTGGCGCTGGCAAGTGTAGCGGCTT
8[172]	TTTTGGCGCGTACTATGGTTGCTAGAATCATATG
10[172]	TTTTAACAGGAGGCCG
12[172]	TTTTCAGTGAGGCCACCGAGTAATAGCAATGAGTAGA
28[156]	AACAGCATCACCTTGCTGATTTT
56[156]	CGCTTTTATTTTCATCGTATTTT
60[156]	CAAAAGGTCTGAGAGACTACCTTTT
70[156]	TTGCCAAAGACAAAAGGGCTTTT
85[158]	CAGATTAGGAGAGGCTGAGACTCCTCTTTT
101[155]	GGCGCACGAAACATGACCCCTAATGCCGTTTCCATTAAACGGGTTTT

AB-Linker strands

1[97]	TTTTTAAACACTATTT
3[108]	GGTATAAATCAAAAGAATAATCGGCAAAATCCCTGA
5[108]	CCTGCCCCAGCAGGCGAAGCGGTCCACGCTGGTTGC
7[108]	AAGATTGCCCTTCACCGCGAGACGGGCAACAGCTCG
9[108]	GCTTGCGTATTGGGCGCCCGCGGGGAGAGGCGGTAA
11[109]	AGAAACCTGTCGTGCCACCCGCTTTCCAGTCGGAC
14[105]	GGGAGTAACGACCGTG
16[115]	TGTTTTGAATGGCTATTAGTGGCACAGACAATATTG
18[115]	TGTGAGGCGGTCAGTATTGAAGATAAAACAGAGGCA
20[115]	AGAATATCAAACCCTCAAACCTTGCTGAACCTCAGG
22[115]	GGTAATAGATTAGAGCCGTAGGAGCACTAACAACGC
24[115]	GGCCCGAACGTTATTAATCGTATTAAATCCTTTGCA
26[115]	CGTCAGATGATGGCAATTATCATATTCCTGATTAAC
28[66]	TCGAAATAAAGAAATTGCATTTGCACGTAAAACAGG

41[63]	ACCCAATAGGAACGCCACAGCTCATTTTTTAAAG
56[66]	ATGCCTGATTGCTTTGAAAAACAATAACGGATTCCA
69[63]	TTGAGATCTACAAAGGCTGGGTAGCTATTTTTGACA
84[66]	AATCAAGAAAACAAAATTGATGATGAAACAAACATG
99[63]	TATGGCATCAATTCATCGGTTGTACCGG
100[66]	GGAATCGTCGCACATAGCGATAGCG
103[63]	GTATGGCTTAGAGCCCAATTCTGCT
104[66]	GGCTGAGAGACTATAACTATATGAG
107[63]	TCACCATAAATCAATTTAATTCGTA
108[66]	TGAAATATATTTGGTTTGAAATACC

SH-probe strands. The red- and green-colored portions of the sequences are complementary to the ssDNA conjugated to the enzymes, and are located in the Left and Right half-cages, respectively.

34[53]	ATGACCATAAATCGCCTGATAAAT GGAGGGAGGG
48[53]	TGTGTCGAAATCCCTCAGAACCGC GGAGGGAGGG
62[53]	CACCCTCAGAGCGCAGCACCGTAA GGAGGGAGGG
51[117]	TTTAGGCAGAGGCATTCAACGCCAACATGTAA CCAGCCAGCC
61[117]	CGAACAAAGTTACCAGAAAGTAAGCAGATAGC CCAGCCAGCC
75[117]	GTAAGCGTCATACATGTGAATTTACCGTTCCA CCAGCCAGCC

Sequences of staple strands in the SS-left half-cage

0[55]	TTGCTTTGACGAGCACGTA
0[79]	GCCGCTACAGGGCGCGTGGTCAAT
1[37]	TAACGTGCTTTCAATTCTACCACCGAGTAAAAGTT
1[72]	AACCTGTTTAGCTAGCTTAGTTTGACCATTAG
1[104]	AGGGCGCTGAACGTGGCGAGAAAGGGGAGCCCCCGATTTAGGTCGAGG
2[55]	GGTGGCATCCTCGTTAGAATCAAATACTATGG
2[87]	GAAATATTTTCATTTGAGTACGGTGCTGAATA
3[37]	ATAGTAGTAGCCTAAATCGAAACTATC
3[72]	GCAAGGCAAAGAAGGAGCTTAATTGTCTGGAA
4[55]	GCATAAAGATTAACATCATGAGTCTGTCCATCAGCAAAATCAC
4[87]	ATCTTAGCAAAATTAACAGGATTAATTCGAGC
4[103]	TGCCGTAAGTCTATCAGTGAACCATTGGAACAAGAGTCCAAAAGAATA
5[37]	GTACCAAAAACAAAATTTTAATACCTA
5[72]	GGGAACGTCAAAGGGCGCGTTTTAGAGAGTAC
6[55]	CAAGGATAATTATGACCCGTGCTGGTAATATCGCGCAGTCTCT
6[87]	GAACGTGGACTCCAGATAGTCAGACGAGAATG
7[37]	AACCCTCATATAGGCCGGAGAGGGGGTGCTTTTGCTATTCGGTTATT
7[72]	TGTGGCAAAATCCCTTTCAGAAAAAGCAAAGC
7[104]	GCCCGAGAGCAGGCGAAAATCCTGAGAGAGTTGCAGCAAGTTTTTCTT
8[55]	GGTGAGAAATTTTAAACAGTGACGCTCAATCGGGGATAGCAAG
8[79]	AAATCGTAAGCGTCCACCAGACGA
9[37]	AGTCAAATCACCTATTTTTTATTTTTGATGTCAATCATAT
9[72]	TAGGCCCTTCACCGCCCTCGTTTAATACTGCG
10[55]	GAGGGTAGCATCAATAAGCGAGAGAATAGTAA
10[79]	TGATTCTGCTAATGCAGAGAATCGGAAGATTGTATTAACTCACATTAA
10[103]	TTCACCAGGCGGGGAGAGGCGGTTTGCGTATTTCCAGTCG
11[37]	AGATCTACAAAGGCTATCAAAACTAGCAATATTTA
11[72]	TCTGGAGCAAAAATCGGCCAACGCTGAGACGGGCAACAGC
12[63]	TAATCGTAGGTCATTGATGCCGGA
13[35]	GTACCCCGGTTGATAATCAGAATATTTTGAGATGCGA
13[96]	TTGCGTTGGTCGTGCCAGCTGCATTAATGCAAGATACATAACAACATT
14[63]	AAACGTTAAAAGCCCCTTCATCAGTTGAGGGCCGC
14[95]	CTAATGAGTGAGCAAGAGTCAGGAGGTTTAAT
15[35]	TAAATTTTTGTTAAATCAGCTTAATTCGCTTGGTAAC
15[80]	GTTATCCGCATAGCTGGCTTGCCCTCTTGACA
16[63]	ATCAAAAACATTTTTTGTGAATTACCTTAAGAAGC
17[35]	GTAGCCAGCTTTCATCAACATTCCGTGGGCCGAACTGCGCAGACGACG
17[80]	TGCCAAGCACGACGTTAACGGTGTGACCTGCT
17[96]	CTGCAGGTAATTCGTAATCATGGTCTCACAATTCCACACATGGGGTGC
18[63]	TCGGATTCTAAATGTGTACCCAAATCAACCTGCGG
19[35]	TTGACCGTAATGGGATAGGTCCATCTGCCGACCCCCA
20[63]	TAACCGTGACGTTGGTGAACGAGGACCAACTT

20[95]	TGGGAAGGAGCTGGCGAAAGGGGGCAGGGTTTTCCCAGTCTTGCATGC
21[35]	GACGACAGTATCGGCCTCAGGAAGATCGCACTCCAGCGCGCATCG
21[88]	CTTCTGGTGCCGGAAAGCAACTGT
44[71]	ATACATTTGCAACTAAGGGCGCGATCATACAG
45[88]	GTTTCATTGAGTAGATGAAAGGAGCCGCCGCGCTTAATGC
46[71]	TAATGCTGCAACAGGTGCAATAAAACTTTTGC
47[48]	GGCCTGAAGCAAACTCTAGCTCAACCAATAAAGCTGAAAA
47[88]	CTTTAATTGGCTTAGAACCCTAAAGAAGGGAA
48[23]	CCAGCCATTCACTTGCCCATATTTAAGGCTTACAATAGCACGAATTCA
48[71]	TTCAAAGCGACTATTAAGCCTTTACTGAGTAA
49[48]	CATTTGTCTTTACCCTGAACCAGACCTGTAATGCCTCAGA
49[88]	GGATTGCACAAATATCGAAAAACCAGCACTAA
50[23]	TACATTGGTGCAACAGTAATTTTCTTAATTGAAAAGCCAAGAGGACGA
50[71]	ACCATAAAGACTGGATGGTAAAGAAACCGTTC
51[56]	AATGTTTATCAAAAATTGCAATGCTTTCAACG
51[88]	GAATCGTCTAAACAGTATAAATCACTATTAAA
52[23]	AACAGAGAAGTAATAAGGATTATATCGTCGCTAGTGAATATAGCCCTC
52[71]	CGATAAAAACATTCAAATAAATTACCTGAGAG
53[56]	GGAATACCACCAAAATTGATATTCTTCAAAAG
53[92]	CAAATCATAACCTGGCCCTGTTTGATGGTGGTTCCG
54[19]	CCCTAATCCTGACAGATGATCTATTGAT
54[31]	GCGAACTGTACGTGGCTTCTGGCC
54[43]	AGTCCCACCAGCTTAAAATTCGCAT
54[79]	ATTACAGGATTATACCCAAATATTGTGAAATT
55[56]	TTTTAAGAACTGGCTCTAGAAAGAAAAAACAGATGAACGG
56[31]	CGGTCAGTAAAAATACAAGGCCGCTTGCGCAT
56[79]	TTCAACTTTGAATAAGTTTCCTGTACGGCCAG
57[56]	AAAGCTGCTCATTCAGTAATCATTAACCAATATAAATTGT
58[19]	ATCTATCATTTTAATTTTAATAAAAATC
58[31]	CCCTCAATAAATGAAACCACCAGATTTTGCGGTTTCTTAA
58[79]	AGAACCGGGACAGATGGTAAAACGTCTTCGCT
59[64]	TGAAAGAGATATTCATAGCGAGTAGGAACGCC
60[31]	AGGTTATCTCAACAGTTAAAGACTGCGGAACAGTATGCGT
60[79]	CCATGTTACGAAACAATGCGGGCCCAGCTTTCCGGCACCG
61[24]	GTCAACACCTACGAAGTTTTCATGTTTTTCAC
61[56]	GCGATTATACCAAGCGCTTAGCCGGTAGATGGACAACCCG
62[23]	AATACATTATTCGACAGCACCAACAAGATTGCTTTGAATATCATTTCA
62[40]	GGCAAAAGAATATAGAT
64[50]	TTGGTAAAATACGTT
66[50]	CTACAGAGGCTTCCATTAAGTCAATCATCATCTTTAGTTTGAGGGGAC
67[8]	TTGTAACATTGGTTT
67[40]	GGTAGCAAAACCTCAATAAGGGAAAACAAACGGCGGA
68[50]	GAAAGACAGCATCGGAAAAAATCTAAAAGGTGAGG

70[50]	TGAGGCTTGCACCCTCAGCTAAAACAGGCATCACCGTCTGGCCTTCCT
71[32]	AACCGATAAAAAGAAGACAGACAAGAG
72[44]	GACAACAACCATCGCCCATTTAAGGGACAGGATTATT
73[24]	ACCGATAGCCGTAACAATTACCCT
74[23]	ACAGCCCATATATGTGTAATGGAAAGTGAATT
74[44]	CTTTCGAGGTGAAGATCGTCAGGGAGTTACGAACGAATTTAATGC
75[24]	GAAATTCGACCTTTTTCTGAGTTTTTTAGTAC
76[23]	AGATTTTCATTTAACACATCAAGATTAGGCGG
76[44]	GGAGCCTTTAATTAAGACGAG
77[24]	TCCAAAAAAGTTTTGTATTTCATTCCCAAATC
78[23]	GTTGACAGGAAACAAAATTACCTGTGATGCAA
78[44]	TGCGAATAATAATAGGAAGTTTTGAGGACTGAAAGGAATATCAAA
79[24]	GCCTGGAATAAACAACCGTCTTTCTTGCTCAG
81[24]	TTTCAACATCCATCGCAAGACAAAGTTAATTTTGAAACATCCAAGTCC
82[50]	TTTCTGTATGGTTTT
84[50]	AGTTAGCGTAAAGTAAATGAAT
86[50]	AACGCCTGTAGCATTCATTGTTTATCAGCTTG
89[32]	CATTTTCATCTGAAATTTTTGCCAGAC
90[44]	GCCACCCTCAGAGCCACCAATGAAAAACGTATTACCG
91[24]	AGAACCGCACCAGTATGAAGCCAG
92[23]	CGCCACTTCATATGCGTACTAGAAAAAGTACC
92[44]	CGTACTCAGGAGGCGTCACCACCCATGTATTGCGCCGACAAT
93[24]	TATCAGGAGTACTGGTTTATACAATTGAGGCA
94[23]	ATAGGTCTATCATAATCGTTAAATCATCCCTC
95[24]	ATAAGTGCAAATAAGGAATAAGTTCCAAAGGT
96[23]	TACCAGGTTTTGAAATCATCTTCTTCAACAAT
96[44]	GATTAGCGGGGTTCAGACGTTCGATCTAAAAAGGCTCCAAAA
97[24]	ATCCATCATATTATTCACCGACCGCTCAGAAC
100[50]	TTTCCCTGCCTATTT
102[50]	GAGTAACAGTGCCCGTGATCGTCGAGAGGGTT
106[50]	GAATTTACCGTTCCAGCTTCACCCTCAGAACC
107[32]	AATGGAAACAGAACAACTCATGGATAG
108[44]	AAATAAATCCTCATTAAATCGCTGAGTAGTAACCGTT
109[24]	TTGGCCTTAGCAAGGCTCCGGGAA
110[23]	GGTCAGCAACCGCGCCTTTATTTTAGATTAGT
111[24]	GACAACCAAAGCCGTTCGGAAACGATTGACGG
112[23]	AAAGTAATACCGCACTCCAAGAACCGCAAATT
113[24]	AGAGCCACACTGTAGCCATCGAGAACATTTTG
114[23]	CGCCAGTTTTATCATTCAATCAATCCAGTTAC
114[44]	CAGAGCCGCCACCTGTATAAACAGTTAATGCC
115[24]	AGATACCGCCATCTTTGCGTTTTCCACAATCA
116[23]	TGAACAAGGTAGAAACTCATAATCCGCAAACA
118[50]	TTTGCCCCCTTATTT

120[50]	AGTTTGCCTTTTTTCGGTCATA
122[50]	TAGCAGCACCGTAATCAGCGAGCCGCCGCCAG
124[50]	CAGTAGCACCAGAAACCATCGA
125[32]	TTAGAGCCACGCAAATAAGAACTCGTT
126[44]	CACCGACTTGAGCCATTTGGTTTTATAATAAGGGATT
127[24]	TTAAAGGTATAATAAGTACCGAAG
128[44]	AGGGAAGGTAAATTCACCAATTTACCATTGATATTCACAAAC
130[44]	AGACAAAAGGGCGACAAGTAGCGACAGAATCA
132[23]	ATAGATAATACAGAGAGTCAAAAAT
132[44]	AAGTTTATTTTGTATCGGCATAGCGTCAGCACCCTCAGAGCC
134[23]	GCCATATTTGTTTAACATACATAA
135[32]	AGGTGGCACAAACGTAGACACCACGGAAT

45[5]	TTTTCAGAATCCTGAACTTCTTTAGATATAGAACAACGCCAACATTTT
65[8]	TTTTTTGCCCGACTTTAGGAGCACTTTTT
69[8]	TTTTTCATATTCCGCCTGCAACAGTTTTT
73[3]	TTTTGAAGGGTTAGAACGGCAATTCTTTT
75[3]	TTTTTGCACGTAAAACAAATTATCATTTT
77[3]	TTTTGATGAATATACAGAAGTTTGATTTT
79[3]	TTTTGGGAGAAACAATATAAATCCTTACAAACATGAGGATTTAGAAGTATTTT
83[8]	TTTTAAGATGATTACCTTTTACATCTTTT
85[8]	TTAATTACAGGTTTAACGTCATTTT
87[8]	TTTTTAAATCAATATCAAAATTATT
91[3]	TTTTGAAAACATAGCGAACCTTGCTTTTT
93[3]	TTTTGAGAAGAGTCAATACAGTACA
95[3]	TTTAACCTCCGGCAAACAAAATTTT
97[3]	TTTTATATGTAAATGCAGCAAAAGCGAATTATCCAAGTTACAAAATCGTTTT
101[8]	TTTTAATGGTGGGTTATATAACTTTT
103[8]	TTTTACACCGGAGAGAGACTACCTTTTTT
105[8]	TTTTTTTAGTATAGATTAAGACGCTTTT
107[8]	TTTTAGTAGGGCCCTTAGAATCCTTTTTT
109[3]	TTTTTGTAATTTAGGCACGCTCAACTTTT
111[3]	TTTTAATAAGAGAATATAAAGCCTGTTTT
113[3]	TTTTCGACAATAAACAAAAGAATAATTTT
115[3]	TTTTACGCGCCTGTTTAGACCTAAAATATTTTAGAACGCGAGAAAACTTTTTT
119[8]	TTTTGTCTTTCCCAGCTAATGCAGATTTT
121[8]	TTTTAACCAAGTTCTGTCCAGACGATTTT
123[8]	TTTTGAATCATTTTTTCGAGCCAGTTTTT
127[3]	TTTTTTAGCGAACCTCCAGCAAATCTTTT
129[3]	TTTTAAGCCTTAAATCACATCGTAGTTTT
131[3]	TTTTTGAATCTTACCAAGGGTATTATTTT
133[3]	TTTTAGAGCCTAATTTGAATCGGCTACGAGCATAAAAATAATATCCCATTTTT
137[8]	TTTTGCAGCCTTCGAGCGTCTTTCCTTTT

139[8]	TTTTATTAACTGTACAATTTTATCCTTTT
141[8]	TTTTAGATAACCGCGGGAGGTTTTGTTTT
143[32]	TTTTCCCTTTTTGCCGATTACAGTGAGGCTATTTT

44[23]	TTAGACAGGAACGGTAATAGCAATAACGCGAGGCGTTTTTT
46[23]	GTAGCAATGAAGTGTTATTCTAAGAGCTATCTAGCAAGAAACAATGAATTTT
47[5]	TTTTTAGTAATAACA
50[15]	CAGATTCATCTGTAAACTTCTGAATAATGTTTT
51[5]	TTTTGTCACACGACCTAGAACCCATCAATATAAA
53[5]	TTTTTGACCTGAAAGCGTAAGAAATAG
55[13]	TTTTACATCGCCATTATTAACACCTGATTATAGGAGCGGGAAATAAA
57[3]	TTTTGCCACGCTGAGAGCCAGCAGCCAAT
59[13]	TTTTCAGTTGGCAAATAAAATATACGTTATTACTCGTATACGGATTC
61[3]	TTTTAACAACTAATAGATTAGAGCC
63[0]	TTTTTTAGACTT
81[0]	TTTTCGCAGAGG
99[0]	TTTTTTTCAAAT
117[0]	TTTTCCTAATTT
128[23]	AAATTCTTCACAAGAAAGCGCTAATATCAGAGTTTT
130[23]	CACCCAGCAACACCCTCGCATTAGACGGGAGATTTT
135[0]	TTTTATAAGAAA
136[14]	GAAAATACGATTTTTATTTATCCCAATCCAATTTT

2[122]	TTTTGGAAAGCCGGCGGCAAGTGTAGTTTT
4[122]	TTTTAAGTTTTTTGGGAGCTTGACGGTTTT
6[122]	TTTTGTTGTTCCAGTTCACCCAAATCTTTT
8[122]	TTTTGGTTTGCCCCATAGGGTTGAGTTTTT
10[122]	TTTTCGCCAGGGTGGCGGTCCACGCTTTTT
14[119]	TTTTCATAAAGTGTAAAGCCACATACGAGCCGGAAGTTTT
16[119]	TTTTCCGGGTACCGAGCTCGCGACTCTAGAGGATCCTTTT
18[119]	TTTTTTAAGTTGGGTAACGCATGTGCTGCAAGGCGATTTT
20[119]	TTTTTCGCCATTCAGGCTGCCCAGGCAAAGCGCCATTTTT
56[92]	TTTTGAGATCGTTGGTTTT
58[93]	TTTTGAGTAATGACGTTTT
60[92]	TTTTTCCGCACAGACTTTT
0[122]	TTTTCGGTCACGCTGCGCGTAACCACCACACCGGGCGCT
12[111]	GGAAACCTCGCTCACTGCCCGCTTTTTT
19[80]	ATTACGCCGCGATCGGAGTACAACGGAGATTTT
45[56]	TTAAATATCGCAGAGCGGGAGCTAAACAGGAGAAGAAAAGTTTT
54[93]	TTTTAACGGAACGC
80[40]	TTTTGAGAATAGACTAAAACGTAATGCCATAAAACACATTGAGGA
98[40]	TTTTGGCTGAGACGTTTCAGCGATTTTGCCAACTAAAGGAAT
116[40]	TTTTCCAGAGCCAGAAAGTATTCGGAACCAGAGAAGGATTAG

129[24]	TGCTATTCGGTAATTGTTGAGTTAAGCAGTTACCAGAAGGTTTT
131[24]	TTTTCATATGGTCAGGGAAGGAACAAAGTCAATAACGGAATACC
133[24]	AAAATAAAGAAAATACGAATAACATAAAAGACTCCTTATTTTTT
134[40]	TTTTTAAAAGAAAAAAATCACAGCGTTTGGAACCGCCTCCCT
136[48]	TTTTGTATGTTAG
138[48]	TTTTGAACTGGCATGATTAAATTACCAGCGCCAA
140[48]	TTTTGAGGAAACGCAATAGAGAACCGATTGAGGG
142[48]	TTTTAGATAGCCGAACAACCAGAATTATCACCGT

Sequences of staple strands in the SS-right half-cage

22[116]	GCGTATTGGGCGCCAGGGTGGTTTTTCTTTTCACCAGCTTGCTTC
23[88]	ATCGGCCAGGAAACAGAATTTATCCAGACGAC
24[71]	TGTCGAAAATCCTGTTTGATGGTGAAAGAATA
24[116]	ATTTGAATTACCTTTTTTAATACGCGCGGCCAGCTGC
25[56]	GCCCGAGAAGTCCACTATTAAAGAGTCTATCAGAACCATCGTAAAGCA
25[88]	AAACAAAAAAGATGATATTTACGATGAAAATA
26[71]	GGAACAAGTAGGGTTGTTCAGCTAAGACGCTG
26[116]	TTTCAATTACCTGAGCAAAAGTTAATTACATTCTGTCAAAATCAT
27[88]	TACAAAATGCCTGATTTGAGCGCTTCACCGAC
28[55]	CTAAATCGGACGGGGAAAGCCGGCAAGGAGCGGGCGCTAGTAACCACC
28[71]	GAGGTGCCACCCAAATGAATAACACAAGAAAA
28[116]	GGGAGAAACAATAACGGATTCCGCGCAGAGTCAAAAAGCATGTAG
29[88]	GAATATACAGATTTTCAGCAGCACTAAGTTTT
30[71]	GAAAGCGAGAACGTGGTTAGAGCCCCCTGAAC
30[116]	ACAGAAATAAAGAAATTGCGTAGTAACAGTATCACCGAATATCAG
31[56]	ACACCCGCATGGTTGCTTTGACGAGAGCGGGAGCTAAACA
31[88]	CTACCATATCTGAATAATTAAGAGAGGAGCGGCCGAACGT
31[104]	TATTTGCACGTAAAT
32[71]	CGCGTACTCGCGCTTATGAGTAACAACGTCAC
32[116]	CCTGATTGTTTGGATTATACTTCAAAATTACTGGTAACGTAATCA
33[64]	GGAGGCCGATTAATATCTACAGGG
33[96]	ATGGCAATCCACCAGAGCTTATAC
34[79]	TCAAGGGATTTTAGACCCTATTATTAGCG
34[124]	TCATTTTGCGGAACAAAGAAATCATCAATATAAT
35[64]	CCACCGAGTTGTAGCAATACTTCTAAGAACTCAAACTATCCGCCAGCC
35[96]	TATTAATTGTATTAAAGAATCATGAGGAAGTTCAG
36[79]	ATTAACCGTAAAAGAGATTAGGATTCTGAAACCAGT
36[124]	TTACAAACAATTCGACAACTCTTAAAAGTGACCCCCA
37[96]	TACATTTGTAGATTAGCAGAGGCCGCTTTTGCAAT
38[63]	ATTGCAACGACGCTCAATCGTCTGTCACACGACCAGTAATCCTTCTGA
38[79]	AATATTACGGCCTTGCAGGAGGTTGAGGGTTG
38[124]	TTTAGGAGCACTAACAACTAAAGGATTTAACTAAAGA
39[96]	AGGAATTGTCAGTTGGCATTGCTT

40[79]	TTCACCAGAAATGGATAACCCATGCCTCAGAG
40[124]	AAACCCTCAATCAATATCTGGAGGAAGGTTGCAGGGA
41[64]	CCTGAAAGAATGGCTATTAGTCTTCATTAAAAATACCGAACGAACCAC
41[96]	AAGCATCAAGCCAGCACAGCGGAG
42[124]	CTGCAACAGTGCCACGCTGAGCCTTGCTGCGGTTTAT
43[80]	CAGCAGAAACAGACAATATTTTTGCGTAAGAAAGTTTTGTCTGTAGCA
43[96]	AGAGGTGAGGCGGTCAGTATTAACACCGC
44[143]	CCATTCGCGAATAATAAAAGCTGCATTCATTAAACCCACC
45[136]	CGCTTCTGGCACTCCAAGTGAATAGCCAGAGGAGAGGCTTTGCGAATA
48[143]	GGGACGCATAGTAAAACGGTGTCTTGTTTTAAGAAATCCG
49[136]	GCATCGTATAGGTCACTTCATTCCGGTAAAGAAATGCAATTCAGTTTG
56[127]	GAAATTGTCATGGTCAACCGTGTGATAAA
60[127]	CTCACATTTGGGGTGCAAGACAAAGAACG
62[95]	ATTAATGATGTAAATCCAATAGTGTACATAAACATCAAGA
62[119]	CGGGAAACGAGACTACCTTTTTAATTAGTACC
63[72]	AGAAGAGTGTCGCTATTGAATAACTGAGACGGGCAACAGCTGATTGCC
63[104]	AGGTCTGACTGTCGTGGGAGAGGCGGTTT
64[135]	ACGGTCCGCAGAAAAGTGAGCTAA
65[115]	TATATAACTATATGAGGCATTCAACGCCAAAGCCGTTTTTAT
67[115]	CGAGAAAACTTTTTATGGCTTAATTGAGAATC
68[135]	CGGAGAATTTGTTAAATCCTGTGT
69[115]	TTTCATCTTCTGAATTCTTACTTTAGTATAGAACGCGAGGCG
73[109]	GAAAAAGCCTGCAGTATAAAGC
75[109]	CAACGCTCAACAGTAGTTCACCGCGCCCAATA
77[109]	GCCATATTTAATTCGAGCCAGT
79[109]	AATAAGAGAATATAAAAGCATCATTCCAAGAA
80[87]	GACAATAACCATCCTAGAAACAAATACCAAGT
80[119]	GACAAAAGCAATAATCGGCTGTCTTCGAGAAACGATTTTTCCCACAAG
81[72]	ATAATATCACAACATGAGTGTTGTTCAATATA
81[104]	AAACCAATGTAAAGTAATTTAACAATTTC
82[135]	TAACACTTAATAAAGCTTTAGGCAGTAAATGC
83[115]	CGGGTATTAAACCGTCAAACAGCCATATTATT
83[136]	CACTCGAATGTACCAACTCAGAGCATCGATGA
84[135]	GAACAAGCACATGTAAGAAGCCTTTCAAGGGT
85[136]	AACCTGTTTTGCGGGAAAACATTATCACAAAT
86[135]	AATGGATTAACGCAAGTTATACAACCTAAATT
87[136]	GGCTTCGAATATTTTAGATAAAAAATTAATGC
88[135]	GTATTCTACATATGCGTTCTAAAC
89[115]	TTTTAGCGAACCTCCCGAAGTGTTGGTGTTCTCCGTG
91[109]	AATCAAGATTAGTTGCGTAAACTGGCATGATT
95[109]	TTGCCAGTTACAAAATAGGCTTTTTAAGAAAA
97[109]	TATCCCAATCCAAATACGTCAATAATAAGAGC
98[87]	GCAGCCTTAGGGTAATGCTTTGAACGTCAGAT

99[72]	AAAGTCAGTACAGAGACAAGTTTTTCCAGTTT
99[104]	AGAGATAATGTTTAACGGCGAATTATTCA
99[120]	AATTGAGTCATAATTATTCATTAAAGAATCAA
100[135]	ATTGCGCCTTTAATTCTCCAACAGAAGTACCG
101[115]	AAGAAACAATGAAAACCGATTGCCAAAGACGTTTGCCATCTT
101[136]	AGCTAGTCAAGCAAACGAGCTTCAAGTAGCAT
102[135]	CCGAAGCCATTAGAGACTAACGAGATCTCAAT
103[136]	GTTCAGAAAAGAGGTCGTACCTTTGCTATCGA
104[135]	GCTTTACATACCAACGAATATAATATATAGAA
105[115]	GAAACCGAGGAAAAAGACACCGTGGCAACCGCCACCCTCAGA
105[136]	TAACGGGAAATTGCTGATTTTTGCATTTCGCA
106[135]	CCCAAAAGGCTCAACAGGACTTGC
107[115]	AAGACTCCTTATTACGCATAAAGGCGATTAGATGGGC
111[109]	TTATTTGTCACAATCCATGAACCAGAGCCAC
113[109]	GGTTTACCAGCGAGGGAGGGAA
115[109]	GGTAAATATTGACGGACAGTCAGACTGTAGCG
116[87]	TTGAGCCACCATCGATAGGTTTAAGTTAGAAC
117[72]	CAATGAAATTTGGGAACGAGAAAGTTGGGGTC
117[104]	GTAGCGACAGGTGAATTACCTTTTACATC
117[120]	GTTTGCCTCCTCTTTTGATGATACAAACAAAG
118[135]	ATAAAGCGTTGAGATTCGACATTCATAGCAAT
119[115]	CGTTTTCATCGGCCCGGAATT
119[136]	TCATAATACAGATACATAGGAATACAAAGCGG
120[135]	CTTATTAGCAAAAGGGAGCAACACCGAAAACA
121[115]	TTCATAATCAAAATCCTCATTCCTTGATATTCGGTCGAAACAGCT
121[136]	GTGAATTAATAGTAAGTAACGCCACAGTCTTA
122[135]	TTTAACCGAACCCTCGGAAACGCACGCAATAA
123[115]	CACCGGAACCGCCGACGGAGG
123[136]	AGCCGGAGAAATAGCGTTTACCAGCCTCAAAT
124[135]	CTCAGAACATATAAAAGGGGTATG
125[115]	GCCACCACCCTCAGAGCCTTCGCCAGCTTGGGGATGT
127[109]	GCCGCCAGCATTGACAAAGGAGCCTTTCAACTAAA
128[119]	TTGAGGCAAATTTCTTCTGAGGCTTATCTAAAATATC
129[109]	CAGACGATTGGAAAGCCAGGGGATCGTCTTTGAGGGAAGTATTAGACT
131[109]	GGAAAGCGCAGTCTCTAACTACAGAGGCACCCTCA
132[119]	TACCGTTCTCCATTAATCATCTTTTTGAGTAACATTA
133[109]	TAAGCGTCATACATGGGCTTAAAACACACGGGTAA
134[75]	GCCTATGCGCCGGAAGGGAA
134[87]	AACGGGGTATGAAAGTATGGAAGGTCCTGATTATCAGATG
135[104]	CAAGCGCGAGGAGTGT
135[120]	TACAACGGAGATTTGTGAATACACCCATGTTATAAGGGAAATTTTCGG
136[103]	GCGATGAGACTCCTCAATAGCCCGTCCTTTGCATAGATAA
137[80]	ATATAAGTAGAGAAGGTCTGTCCAAATTATCA

137[128]	AATACGTAGCAACGGCTGACCAACCAAATAAATCATCATT
138[103]	CTTTTTAGGTGTATCACTCATTTTAGCCGTCACAGTTGAA
139[80]	CCACCACCCCGTACTCTGGTAATATCACGCAA
139[128]	GCAGCGAACCGATATATTCACAAAGTAATCTTTCCCTCAG
140[103]	GTTAAGGATAGCAAGCACAGCCCTCAAATCAAAAAATCTA
140[143]	ACGCATAAAGACAGCAGTACAGACTTTGAAAGATTGCCCC
141[80]	TTCCACAGCCAATAGGTATTTACATCCAGAAC
141[104]	TTTCGAGGTGGGTTT
141[128]	TGATACCGTCCAAAAGAACCGGATTCAGCCAC
142[103]	CAGCTAGTTAGCGTAAAACAGTTTGCAAATGAGATAAAAC
142[143]	AAAAAGGCATAGTTGCCATCAAGACAGGCGCAATTTCAAC
143[66]	GGGATTTTGCTAAACAACTTTCCGATCTAATACGTGGCTTGGCAGA
143[104]	TTTGAGAATAGCCTT
143[128]	GGAATTGCCATTCAGGTCCGGCAC
46[154]	TTTTTCAGGAAGATCGTGCCGGAAACGTAACATTTTTTCACGTTTTTT
50[154]	TTTTCCGTAATGGGAACCGTGCACTAAAGTATGTTTAGACTGGATTTT
54[151]	TTTTCTGGCCTTAAAGGCCGGAGACTTTT
56[151]	TTTTCCAATAGGTGATATTCAACCGTTTT
58[151]	TTTTTAATATTTTTGAGAGATCTACTTTT
60[151]	TTTTATTGTATACCTGAGAGTCTGGTTTT
64[156]	TTTTAGCAAACAAGAGAATAAAGCTTTTT
66[156]	TTTTAAAGGCTATCAGGTGACCCTGTTTT
68[156]	TTTTTTCTAGCTGATAATTTTTAGATTTT
70[156]	TTTTAGTCAAATCACCAGCCTGAGTTTTT
74[151]	TTTTACCCTCATGTAGATTTAGTTTTTTT
76[151]	TTTTTAATACTTTAGCTATATTTTCTTTT
78[151]	TTTTAAATCGGTAAGGTGGCATCAATTTT
80[148]	TTTTCAAGGCAAAGAATTAGCAAAACCTAATCGTAAAACTAGCATGTTTT
82[156]	TTTTTTCTACTAATAGTAAGCGAACTTTT
84[156]	TTTTATTTGGGGCGCGAAATTGCTCTTTT
86[156]	TTTTGACCATTAGATACGGATGGCTTTTT
88[156]	TTTTTTCCCAATTCTGCATATGCAATTTT
92[151]	TTTTTAGAGCTTATCGTCATAAATATTTT
94[151]	TTTTCTTTTGATAACGAGAATGACCTTTT
96[151]	TTTTCAGACCGGTTTACCCTGACTATTTT
98[148]	TTTTAAGCCCGAAAGACTTCAAATATTCTCCAATAAATCATACAGGTTTT
100[156]	TTTTTTATAGTCAGAAGCCACATTCTTTT
102[156]	TTTTATAAATCAAAAATAAAGGAATTTTT
104[156]	TTTTTTCATTGAATCCCACGACGATTTTT
106[156]	TTTTTAGCGTCCAATACTTGCAAAATTTT
110[151]	TTTTAAAAACCATAGTAAATTGGGCTTTT
112[151]	TTTTTACGAGGCCCTTATGCGATTTTTTT
114[151]	TTTTAACTAATGCCAGTCAGGACGTTTTT

116[148]	TTTTATTATTACAGGTAGAAAGATTTAAATCAAAAAGATTAAGAGGTTTT
118[156]	TTTTTGGGAAGAAAAATGAACGAGGTTTT
120[156]	TTTTTAAGAACTGGCTCAGGACAGATTTT
122[156]	TTTTTTGAGATGGTTTATAGGCTGGTTTT
124[156]	TTTTCGAGAAACACCAGCCCAAATCTTTT
128[151]	TTTTCTGACCTTGCCGACAATGACATTTT
130[151]	TTTTTGAACGGTTCGTTTT
132[151]	TTTTCGCAGACGACGAAGGCACCAATTTT
134[148]	TTTTAAATTGTGTCGAAATCCGCGATTAACGAACTAACGGAACAACTTTT
136[156]	TTTTCCTAAAACGAAAGAGGCAAAAATCATCGCCTGATTTTT
138[146]	TTTTGAACGAGGGTAATGCCACTGTCAATCACTTAGCCGCTACGTTATTTT
44[154]	TTTTCAGGCAAAGCG
47[136]	GCTGACGACAGTATCGGAAGTTTTAGGCTTGCCCTGATTTT
48[154]	TTTTGCCAGTTTGAG
51[136]	GGAACAAATAACAACCAGGGTGAGCCTGTAGCCAAAAATAATTCGCGTTTTT
51[144]	CGGCGGATAATGTGTAATATAACAGTTGATTTT
62[148]	TTTTTCAATCATATGTACCCCGGTTGATCCAGT
65[136]	TGATGTTGAGCAAATACCCCAAAAACAGGAAGTTTT
67[136]	AGCTATTTTGTTAAAATTTAAATTGTAAACGTTTTT
69[136]	TAATGATAAACGCCATTCAGCTCATTTTTTAATTTT
71[115]	TAAGGCGTTAAATAAGAAAAACGTCGGATATTAAATGTGAGCGAGTTTT
140[156]	TTTTACAACCATCGCCC
142[156]	TTTTGAAAATCTCCAAA
23[32]	TTTTTTGCAGCAAGCGGTCCCCTGGCCCTGAGAGAGTTTT
25[32]	TTTTAATCCCTTATAAATCAGTTCCGAAATCGGCAATTTT
27[32]	TTTTCAAAGGGCGAAAAACCACGTGGACTCCAACGTTTTT
29[32]	TTTTCCCCGATTTAGAGCTTGAACCCTAAAGGGAGCTTTT
31[32]	TTTTGCGGTCACGCTGCGCGGGCGCTGGCAAGTGTATTTT
37[40]	TTTTTCACTTGCCTGAGTAGTTGATTAGTAATAACATTTT
39[40]	TTTTGAAATACCTACATTTTAGGAAAAACGCTCATGTTTT
41[40]	TTTTCCAACAGAGATAGAACAAAAGGGACATTCTGGTTTT
43[40]	TTTTAGCCCTAAAACATCGCTAATGCGCGAACTGATTTTT
53[114]	TTTTTCTAGAGGATCAACGCATGCCTGCAGGTTTT
55[111]	TTTTATTCGTAATTATCCGCTTTTT
59[111]	TTTTTGTAAAGCCAATTGCGTTTTT
63[58]	TTTTAGATTAATGCATTTT
81[59]	TTTTCTGAATAAAAATTTT
99[58]	TTTTTGAACAAGCAATTTT
117[59]	TTTTCCGGAAGTGCCTTTT
135[58]	TTTTTCGGAAAGGAACGGCAGTGAGGTTTT
137[67]	TTTTGTCGATAGTACTTTT
139[66]	TTTTCGCCACTACCGTTTT
141[67]	TTTTAACGCCGTCTTTTTT

22[55]	CTTCACCGACGCTGGTTTGCCCCAGCAGGGAGTAATTAATTTTCCCTTTT
33[32]	TTTTTTCCTCGT
34[47]	AATCCTGATAGAATCAGCACGTATAACGTGCTTTTT
35[40]	TTTTGAAGTGTTTTTATAATTACGCCAG
45[114]	GGGCGACCACCAGAGAAAGGAAAATTGTATAACCTCAAATATCTTTT
47[114]	CCAGCTCGTAGAAAATATTTT
49[114]	GTTTTCTTGAAGCCTTATTTT
51[114]	CCAGTGCATAATTACTATTTT
57[111]	TTTTTTCCACACAACATATTTATATTTTAGTTAA
61[111]	TTTTTCACTGCCCGCTTTAATGCTTAGGTTGGGT
66[135]	CCAATCGCCTAATGAGTTCGCATTAAACGAGCCGGAAGCATTTT
70[135]	AAATACCGTAGCTGTTCAGCTTTCATCCCCGGGTACCGAGTTTT
72[127]	ACCGGAATCCAAGCTTGACGTTGTAAAACTTTT
90[127]	GGGAGGTTCCAGTCACAAGTTGGGTAACGTTTT
108[127]	TTAGCAAAGGCGAAAGGGCCTCTTCGCTATTTT
126[127]	CAGAACCATCGGTGCGCTGCGCAACTGTTTTTT

SS-linker strands

44[114]	GGGTCCCAATTCTGCGAACCCATATAACAGTTGATAA
46[114]	TTAGGTCATTTTTGCGGATGCTCCTTTTGATAAGACG
48[114]	CCAGAAGCCCGAAAGACTTTCAAAAAGATTAAGAGGG
50[114]	GACTCCCCCTCAAATGCTTATAAATATTCATTGAAGG
52[114]	TCGAGTAAGAGCAACACTAAGGAATTACGAGGCATAC
54[111]	CTCTTAATAAAACGAACTGAAGAAAAATCTACGGA
56[111]	CACGTAGTAAATTGGGCTTAGAAACACCAGAACGAAA
58[111]	TAAGCTGACCTTCATCAACAGGCGCATAGGCTGAG
60[111]	TGCATAAATTGTGTCGAAATTTGTATCATCGCCTGGC
63[40]	AAAACATAGCGATAGCTTTTAGAATCCTTGAAAGA
81[40]	GGACAACAATAGATAAGTCGAACGCGCCTGTTTATGT
99[40]	TAAGACGGGAGAATTAACCAGGGAAGCGCATTAGA
117[40]	CGGATTACCATTAGCAAGGAATCACCAGTAGCACCAA
135[40]	ACATGCCCCCTGCCTATTCGTATAAACAGTTAATA
137[48]	ACGCAGGCGGATAAGTGCCGGGTTTTGCTCAGTACCA
139[48]	CAACGCCACCCTCAGAACCGCCACCCTCAGAACAA
141[48]	AAACACCAGTACAAACTACTAACACTGAGTTTCGTCC
143[48]	TAAATGAATTTTCTGTATTCCAGACGTTAGTAAGC

SS-probes. The red- and green-colored portions of the sequences are complementary to the ssDNA conjugated to the enzymes, and are located in the Left and Right half-cages, respectively.

94[44]	GATATAAGTATAGTGACACAGACAGCCCTCATGGAGGGAGGG
104[50]	CTTTTGATGATGTCAGTGCCTTGGAGGGAGGG
110[44]	CATTGACAGGAGGATTTAAGCGTCATACATGGGGAGGGAGGG

87[115]	GCAAGCAAATCAGGCTTATTTGCACCCAGCTCCAGCCAGCC
93[109]	ACAATTTTATCCAGAGCCTAATCCAGCCAGCC
103[115]	GTAAGCAGATAGCTATAATAGAAAATTCATATCCAGCCAGCC

DS Full-Cage design

Cross-sectional view

Sequences of staple strands in the DS-left half-cage

1[16]	TTTTCAGTACAAACTACAACCACTGAGTTTCGTCACTTTT
3[16]	TTTTAATTTTCTCAGCTTTCCGGCATTTT
5[16]	TTTTTCACGTTGGAGATCTTTTT
7[16]	TTTTATACCGATAGTTGCGCTTTCTTAAACAGCTTGTTTT
11[13]	TTTTCCATTAAACGGCAAGCGCGAAATTTT
12[31]	GATTATACGTAAAATATGTTTAGAGTCACCCTGTTAAAGGCCGCTTTTTTTT
13[13]	TTTTCAAAGTACAACAACCGAACTGATTTT
14[31]	CATAAGGGGGAGATTTAAGAAGTTTTGCCTTTT
15[13]	TTTTCCAACTTTGAAAACGTAACAAATTTT
16[31]	CCCAAATCAGAGGACACCCTCGTTTACCATTTT
17[13]	TTTTGCTGCTCATTCATGCGATTTTATTTT
18[31]	ATTACCTTAGTGAATATACGAGGCATAGTTTTT
19[13]	TTTTAGAACTGGCTCCGGTTTT
20[39]	TAATAAAACGAACTAAATTATACCGATTTAGGAATACTTTT
21[21]	TTTTAACAACATTATGCTTCAAATTCAAATAGAGAGTACCTTTATTTT
23[19]	TTTTCACATTCAACTAATGAAAAAGATTAAGAGGAATTTT
25[19]	TTTTAAGAGCAACACTAGACTATTAAATCAAAATCAACATGTTTTATTTT
27[19]	TTTTGACGACGATAAAAACGACAGTTCAGAAAACGATTTT
29[19]	TTTTAGAGGGGGTAATAATAAATATAGCGTCCAGTAGATTTAGTTTTT
31[21]	TTTTGATTCATTGAATCCTTTT
33[13]	TTTTCCCTCAAATGCTTTAAGGTGTGTCTGGAAGTTTTTT
35[13]	TTTTGAATGACCATATAGTCAGAAGCTTTT
37[13]	TTTTAAAGCGGATTGCATCAACAGGTCATTTTTGCGTTTT
39[13]	TTTTGCCCGAAAGACGCGTTTT
41[21]	TTTTAACCAGACCGGACATTATGAAAGCTAATCAACGCAAGGATTTTT
43[19]	TTTTATTGCTCCTTTTGCATAAATTAAGCAATAAAGTTTT
45[19]	TTTTGATGGCTTAGAGCCCAATAAATACTAATATGAGAAAGGCCGGTTTT
47[19]	TTTTAATATGCAACTAAAAACGCGAGCTGAAAAGGTTTTT
49[19]	TTTTTCATTCCATATAAGTCAATAAACCATTAGATTT
51[21]	TTTTTTGCCTGTTTAGCTTTTT
53[13]	TTTTATATTTTCATTTGGGGTCCAATATGATATTCATTTT

55[13]	TTTTGGCATCAATTCTCATACAGGCATTTT
57[13]	TTTTAGGCAAAGAATTAGCAAGCATATATTTTAAATTTTT
59[13]	TTTTCCTCAGAGCAT
61[25]	TTTTCCCTGTACATTTTTTCATTAAATCTGGCCTTCCTGTTTTT
63[19]	TTTTAAAAATTTTTAGATCCTAAACGTTAATATTTTTTTT
65[19]	TTTTGCAATGCCTGAGTAAACAGGAGGTTGATAATTGACCGTAATGTTTT
67[19]	TTTTAGACAGTCAAATCTGTACCCCTTTT
69[19]	TTTTACCGTTCTAGCTGGAGCAAACATCAGGTCACTC
70[27]	TTGAAAAATCTCGCGAATAATAATTTTTTTTT
71[17]	TTTTAAAGGCTAAGAGAATCGATTTT
73[13]	TTTTTGAACGGTAATCGTAAAACTGCATCTGCCAGTTTTT
76[23]	AGATTGTATAATTTT
77[13]	TTTTGCAAATATTTAAATTGTTTCCCGTCGGATTCTTTTT
79[13]	TTTTGTTAAAATTCG
81[25]	TTTTACCAATAGTCGACTCAGTGCCAAGAAATTGTTATCCTTTT
83[19]	TTTTAGCCAGCTTTCATATACAGTCACGACGTTGTATTTT
85[19]	TTTTCCGTGGGAACAAACAAGGCGAAGCTGGCGAACTCACATTAATTTTT
87[19]	TTTTGGATAGGTCACGTGCTCGGTGCGGGCCTCTTCTTTT
89[19]	TTTTTTGAGGGGACGACGCCATTCACGGAAACCCGTATTGGGCGTTTT
90[27]	CAGCGTATGGGACAGACGTTAGTAAATGTTTT
91[11]	TTTTCCGCTTCTGGTGCGGCTGCGCAACTTTT
93[13]	TTTTTGTTGGGAAGGGCGATATTGTCGTGCCAGCTGTTTT
95[13]	TTTTGCTATTACGCCTTAAGTTGGGTTTTT
97[13]	TTTTAACGCCAGGGTTTTCCAAAGTGTAAAGCCTGGTTTT
99[13]	TTTTAAACGACGGCCTAGAGGATCCCCGTTTT
101[11]	TTTTGGTACCGAGCTCGAATTCGTACAAAGGGCATTAAAGA
103[19]	TTTTGCTCACAATTCCATGTTGTTCAGAATAGC
105[19]	TTTTGGTGCCTAATGAGCGAAATCGGAAAATCC
107[19]	TTTTTGCGTTGCGCTCAAGCGGTCCCCTGGCCC
109[19]	TTTTCATTAATGAATCGAGACGGGCAACAGCTGATTTTTT
111[21]	TTTTCCAGGGTGGTTTTTCTTTTTACCGTAAGCCTGTAG
113[13]	TTTTGCCCTTCACCGACGCTGGTTTGTTTT
115[13]	TTTTCCCCAGCAGGCGCAAAATCCCTTTTT
117[13]	TTTTTATAAATCAAACAGTTTGGAACTTTT
119[13]	TTTTAAGAGTCCACT
121[24]	TTTTGAAAAACCGTCTATCATCCAACGTATCATGG
0[55]	CCAATAGGAACCCATGATAACGTGTTAGAGAGG
1[40]	CATTCCACAGTTTTGTTTAAAAATCCATCAGGA
1[72]	TCGAGAGGTCAGTACCAGGCGGATTAACAGTG
1[88]	AAGTATAGACCCTCAGAGCCACCACCCTCATTTTCAGGGAAAGTGCCG
2[55]	CGATCTAAAGACAGCCAAGGGATTCTTTCCTCGCTTTGAC
3[40]	AACAACTTAACAACTAGAACCTACTAAGGAGAG
3[72]	CCCGTATAGGGTCAGTGCCTTGAGCACAAACAAATAAATCGATTGGCC

4[55]	TAGAAAGGTCAACAGTTTCAGCGGTAGCGTAA
4[87]	TTTTAACGAACAGTTAATGCCCCCATTAGCGGGGTTTTGCGTTGATAT
5[40]	AGGCTCCATTGCTTTCATTTTAGTTGAATTCTGC
6[55]	TTATCAGCAAAGGAGCAACAGAAACATA
6[71]	TTGATATTCGCCTCCCTCAGAGCCGAGCCACCACCGGAACCAGTAGCG
7[40]	ACAACAACCTGAGGCTCATTACCGCTTATCC
7[88]	CAGAACCGTTGAGGCAGGTCAGACCTCATTAAAGCCAGAAGTAATAAG
8[55]	TTCGGTCGCATCGCCCTAATGGTTTAAT
9[40]	AAAGACAGCTTTGAGGCACTACGA
9[72]	ACAGAATCATAGCAGCGTGAATTATCACCGTCAAATTATT
10[31]	CTTTTTCATGAGGAAGGCGGGATC
10[55]	TACAGAGGCATCGGAAATAGAAGGCGCCCAATTTTT
10[87]	AACCATCGAAGTTTGCCTTTAGCGAAAATCACCGGAACCAGCCACCCT
11[48]	AGGCACCAAAACACTCGCGTTTTAGCGAA
11[64]	CGAAAGAGACCGTAATGCAACGGC
12[63]	ATACACTAACCTAAAAAATCAGATCGAGGGTACCGATATA
12[79]	CATTAAAGTTTATTTTGTCACAATGACACCACGGAATAAGTACCCAAA
12[95]	ATTGACGGACCGACTTGAGCCATTGAAACGTCACCAATGA
13[48]	AATTGTGTCGGAACGATTTTGAAGCCTTA
15[48]	ACCAGGCGTTGACAAGTATCCTGAATCTT
15[64]	GGCTGACCCTCCATGTTACTTAGCCGAAATCCGCGACCTGGCAAAAGA
15[80]	AGAACTGGCGCAATAATAACGGAAAGAGCAAGAAACAATGGTTAAGCC
15[96]	AGACTCCTATAAAAGAAACGCAAACAATAGAAAATTCATAAGGTAAAT
17[48]	GAAACACCTAATTTCATTTCCAGATATTATTTAACG
18[63]	AGATGGTTAGAACGAGTAGTAAATTTCATCAAGAGTAATCCATAGGCT
18[79]	CAATAATAAAAACAGGGAAGCGCATTAGACGGGAGAATTAAACCCACA
18[95]	AGAATTGAAAATAGCAATAGCTATAAGGAAACCGAGGAAACATGATTA
19[58]	GAGAGAAACAGCCAGCCTAATTTGCCAGTT
22[39]	TCAGTTGAAGTCAGGACATTGTGA
22[71]	ACAAAATAATAACATATGGGCTTG
23[40]	CATAAGTCACTTTAATCGTTGGGAAGACTTTACA
24[38]	AAGGAATAGGCTTGCATTCATTA
24[60]	ACCAACGCTAACGATCCTAAT
25[41]	ACAATTTAACCGGATCCTGACGA
26[41]	TTTGCACTAAGATGAACGCGGTCAAT
26[60]	AATCAAGATTAGTATAATCGG
27[39]	TAGCGAAGGGGCGCAGAGTGTACAG
28[37]	TTGCAAGTATCATCCCCCCAGC
28[60]	CCTCCCGACTTGCCACTCATCCTGTCTTTGTATCATATGCGT
29[42]	CGCGAGATCTTTGAGCCTGATA
30[40]	GGTATTAAACGTAATGCACTAAAGA
32[51]	CATCACCGACCGACCGGAATACGCGAGAATAACTATTTTT
32[66]	AGCCGTTTTTAAGCAAGCA

33[56]	GAGAACAAGAATAAACTGTGATAAATAAGGCG
36[55]	TTACGAGCATAAAGCCAACGC
38[55]	TGTTTATCACGCCAACTAATAAGAATTAATTAACCTTGCTCTTTTTTA
39[52]	CGCCAAACAACAAAAGTACCGACAAAAGAGTGAATA
40[39]	TAATTCGATACAGGTAGAAAGCCAATCTACGT
42[38]	AGGATTATCGCGTTTATAAGTCCTGCAGATA
42[71]	GGCATTTTCGAGCCAGATGTAATTTAGGCAGA
43[41]	TTTAACAAACAATAGTAATGCAGATCATTCA
44[40]	AGAATCAGAAGAAAAATTTTACCCTTCACCAGCT
44[60]	TCAACAGTAGGGCACGCTGAGATTTTCCCAAAC
45[39]	CTGAATAGTATGTAGAAAATATCCCAGCCGCCAA
46[37]	TGTAGCATCAGGTCTTCCAAGAACCAAAA
46[60]	TATACAAATTCTTCTTTTAAAAAATCATTACAAAATTGAG
47[41]	CTGTTTACCTTATCAACCAATCATGCTAT
48[40]	ACTAGAACGATTAAACCGAATCGTCGTACTAAGAA
48[71]	TTTTTTAAATAAGCA
49[39]	TTCCCAAATGTAGGAATAAGTACCGGGGGAGGCTT
50[37]	GAACGAATACTGCGTGCAGGGACAGCAGCG
50[63]	CCTAAATTACGCATAAGTATCGGT
51[52]	TTCAAAACTTTTAATTGCGTAGATT
51[56]	TTTTTTTTCTTCTGA
54[66]	GGTTGGGTTATTTTT
57[56]	TTTTAAGAGTCAATA
61[39]	TGCGGGTACTCTGTAAATACCAAAAAAGCAAACTCCAATATTGTTCAGC
61[56]	ATGGAAACCTAATAGATTTAGAAGAATCAACACAATCAATATCTGGTC
62[38]	CTTTATTATCGGTTGCTTGAAAAATAGCCATA
63[41]	ATCAAGATTAGAATCTCGTCGCTGAACAGGTC
63[52]	AAAATTCATTTGTTTGAGGATTAGAGCCAGGAAGGT
64[40]	GATGAACTCGATAGCTTATTAACATTTAATTG
64[51]	CAAATTTTAAAAAACAATTCCAAACCCTGTTG
65[39]	AGGTAAAGTAGGTCTGAAGATTAAGTTAATTG
66[38]	AAAAGGGGTAGTAGCGCTGATGCAGTAAAAGC
66[71]	GGATTCGCCTGATTGCCGGGAGAAATTCATCA
67[42]	AGTACCATGTAAATGAGACTACACCATAATGC
67[52]	ACATTTTGAATAGAGCGGAAGCGGAACATCTAAAGCATCAC
68[39]	ATATAATCAATCGCAACGCAAATGCAGTTGA
68[60]	TTCAGGTTTAACGTACTTCTGATATAATCATTAACACCGCCT
69[39]	AATGCCAGATCAAATATGACAAAGACATAATT
70[38]	GGTAGCTATACATTTGAGGTGAACGACAATG
70[62]	CACGTAACTTTAATTAGTGAGAA
71[52]	TCAAAATAATGGGCAGAAGATAAAA
71[56]	TTTTTTTAATTATTT
81[39]	CATCAATTGGTCAATAGAATCAGCTATACTTT

81[56]	TATCTAAATTGACGCT
82[38]	TTCGCGTTTTTGTTAGTATTAAAACCACAAAC
82[71]	AGTTGGCATATTTT
83[40]	AATGTTATGACAACTCATAATACAAATAGAAGC
84[38]	GTAACAAGCCCGAACAGCCCCAAAATGTGT
84[60]	CTTGCTGAACCTCCAGAGATAGATTCACCTGGTAATATCCAG
85[42]	GAAAAAAAGAAACCGTTATTAAAGAAGAT
86[39]	CCAGCCGGATCAGAAACAATCATAACCCAGTAAC
86[60]	GCAACAGTGCCACAGAATACGGAAC
87[39]	GATGGGAGTCTGATTGTACCAGAAGCCAAGATTC
88[37]	TAACCGTAGCATGTAGAGTCTGATAAATT
88[60]	CAGAGGTGAGGCGACTGATAGTGGCACAGAGTAAAAGAGTCT
89[39]	TCGGCCCCAAAGGGTTATTGGATTATCAGATGA
90[38]	AGATCGCATTGCCTGAAGGAATTCAAAAAAA
90[52]	TTTACGAAACCGATTT
92[55]	CCCTAAAAAGGAACGGCAGTGAGGATGCGCCGTAACCACC
96[51]	CCTTGATTAGTAACTATCGGCGGCGAACGATTTAGA
101[39]	TCATAGGCCTGAAATGGGCCTGCAGGGAACGC
102[37]	CTGTGTGCTTGCATGACCAGTACAACATTA
102[60]	AACAATATTACCGCACTAAATTTTTGGGG
103[39]	TACGAGTGCAGTCACACATTATTTAGAAAAATAA
104[38]	GCATAAAGGGACATTATGTGCTGCGGAGCAAAT
105[42]	TTCTTTCTGACCTGCTGGCCAAAAAGAGCGA
105[64]	TTTTTTTACTTGCCT
106[39]	TTGTAGCTAAAGGGGGTTTGAATGTGGTGTA
106[60]	GTCCATCACGCAACGCTGGCAAAGCGAAA
107[39]	CTTTCCCCGACAATATTAAAGCGTAGCTGAGAG
108[38]	GAAACCTAGTCTTTACGCCATTCGACAGTA
109[39]	CGCGGGGACCATCGCCAATGCGCGAGTCCGCATCG
109[64]	TTTTTTTAATCCTGA
110[38]	CGGTTTGAGGCAAAGCGTCTTTCTTTTGCTA
112[47]	GAGCACGTCGCGCTTACCAAGTCGG
113[32]	TGAGAGAGCACCAGTGGCCAACG
114[47]	ACACCCGCCGCTAGGGATTAACCG
115[32]	TGTTTGATTTGCAGCACTGCCCG
116[47]	GGAGCGGGGGGAAAGCCCTCCGGAA
117[32]	CCGAGATAGGTGGTTCTGAGCAATAC
118[47]	GCTTGACGCCGTAAAGCCACTGTTTC
119[32]	ACGTGGACGGGTTGAGCACAACA
0[111]	TTTTCACCCTCAGAACCGCCCCCGGAATAGGTGTATTTTT
2[111]	TTTTCAAGAGAAGGATTAGGTGCCTATTTCGGAACCTTTT
4[111]	TTTTATACAGGAGTGTACTGTGGAAAGCGCAGTCTCTTTT
6[111]	TTTTCAGCATTGACAGGAGGCCACCCTCAGAGCCACTTTT

8[111]	TTTTCCATCTTTTCATAATCTCAGACTGTAGCGCGTTTTT
10[111]	TTTTCAAGGCCGTGGGAATTAGAGCCAGTTTT
12[119]	TTTTCCGATTGAGGGAGGGATGGTTTACCAGCGCCATTTT
14[119]	TTTTATAAAGGTGGCAACATTATTACGCAGTATGTTTTTT
16[119]	TTTTCGAACAAAGTTACCAGCTTACCGAAGCCCTTTTTTT
18[119]	TTTTCTAATATCAGAGAGATACTGAACACCCTGAACTTTT
20[62]	TTTTGAAAATAGCAGCAAAATCCAAATAAGAAACGACGACAATTTTT
40[71]	TTTTTCCAGACGATTTTTTGTTTT
80[71]	TTTTACTAACAAAGTACATATTTT
82[51]	TTTTAAAGCATTGGCACAATCGTCATTGCAACAGGAAAAATTTT
104[71]	TTTTGAGTAGAAGAACTCAAATAACATCAGGGAAGAAGTGTAGCTTTT
108[71]	GAAGTGTTTTTATAATTACGCCAGCTATGGTTGTTAGAATCAGAGCGGTTTT
110[71]	TTTTAACAGGAGGCCGATTACTCATAGTTAGCAAGCTTTT
114[63]	TTTTGCTGCGCGCTACAGGGTTTT
118[63]	TTTTGAGCCCCCGTGGCGAGTTTT
120[47]	TCGAGGTGCGATGGCCCACTACGTTTTT
120[63]	TTTTATCAAGTTCGGAACCCTTTT

Sequences of staple strands in the DS-right half-cage

1[136]	TTTTATTGACGGACCGACTTTTTT
3[136]	TTTTTTTGGGAAACCATTAGTTTT
5[136]	TTTTCGGAAACGATCAGTAGTTTT
7[136]	TTTTAATCAAGTATCGGCATTTTT
9[136]	TTTTTCATAGCCAAAATCACCTTTT
11[134]	TTTTGAGCCACCACCGGAACCGAGCCGCCACCGTAACAGCAAGCCCCAGACGT
13[134]	TTTTCCTCAGAGCCACCACCCTACCAGAACCACCACCAGATTTT
15[134]	TTTTGCCAGCATTGACAGGAGGTTGAGAGATCAGAACCGCCAC
17[134]	TTTTCAAACAAATAAATCCTCAAATGGAAAGCGCAGTCTCTTTT
19[134]	TTTTTACCGTTCCAGTAAGCGTCATACAGCGGGGTTTTGCTCA
20[119]	TTTTTTTTAACGAAACATGAAAGTATTATTTCGAGG
21[96]	TTTTGGAACCTATTATTCTGGGGTCAGT
25[136]	CCGTACTCTTGGCCTTGATTTT
29[136]	CCCATGTACCCTCAGAACTTTT
40[135]	CAGCTTGCAGAGGCTGAGACTCCTATACAGGAGTTTTT
41[79]	TTTTAACCATCGCCCACGCATTTTTAAGAACTGGCTCATTTT
41[104]	TATTCGGTTTAAACAGCTTGATACTTTT
61[96]	TTTTAAAAATCTACGTTAATGAATTACCTTATGCGAAACCGATA
81[78]	TTTTGAACGAGTAGATTTAGTTTTGTAAACGTTAATATTTTTTT
81[104]	AGATACATGGAAGTTTCATTCCATTTTT
101[80]	TTTTTTCGCATTAAATTTTTCTATTAAATTTT
110[155]	GCAACATTAAAGATTCAACCGATTGAGGGAGGGAAGTTTT
111[88]	TTTTGTGCTGCAAGGCGATTAAGTTGGGGCGATCGGTGCGGGCCTCTTCGCTTTTT
113[88]	TTTTTGGTCATAGCTGTTTCGCATGCCTGCAGGTCGTTTT

115[88]	TTTTGCTTTCCAGTCGGGAAAGCCTGGGGTGCCTAATTTT
117[88]	TTTTCGCCTGGCCCTGAGAGGCGCCAGGGTGGTTTTTTTT
119[88]	TTTTGTTCCAGTTTGGAACACGAAATCGGCAAAATCTTTT
121[70]	TTTTGCGAAAAACCGTCTATCAATGGCCCACTACGTGAAGAGTCCAGTTAAATC
20[103]	GCCTTGAGTAACAGTGCCCGTATAAATTTT
1[168]	ATAGAAAAAATAAGTTCTGGTCAGAGGTTAT
2[151]	TCACCGTCAAATTATTAGCGCCATAAGAACTCTAATAACA
3[168]	ACATATAAGAAAATACTTGCTTTGTTAATCCCCC
4[151]	GCACCATTTTAGAGCCGCC
5[168]	TCCTTATTCAAAAGAAAAATATATATGGTTT
6[151]	GCACCGTATCACCAATCAGTTCAGAAAAC
7[168]	ACCGAGGAGCCGAACACCAAGAACACAAGCA
8[151]	GCGTTTTCTTGCCTTTCATCGCCTGATAA
10[151]	TCATAATCCCCTTATTACT
12[160]	AAGACTCAGCCTCCATTCAGTACAAAGCGTTTGACTGTAGC
14[162]	ACACCCCCGCCCAGAGTGACAGGGATACTGAGTTTCCCTCATAACGC
16[161]	CAGAGGCAGGTCAGACGAAGGAGGTTCGGAATAGATTTTTT
18[161]	CCAAAGCCAGTTAAATAAGTATAGCCTAGTACCGAGTGAGAAAACA
20[151]	TTTTGATGCAAGAGAAGGATTAGGATACCTTTAA
22[168]	CTACAAAGCCTAATTTGCCCAAT
23[147]	GTACCAGGCGGATAACGAAAATC
24[165]	ATATAAGAAACGATCCTTTA
26[167]	AACGCCCCATAACATAACTGA
27[147]	CCTCAGAACCGCCGAGATGAATT
28[165]	CATTTTACAAAGTCAACCCAC
30[168]	AGCCGTCACGAGTTAAGCAATAGCTCCATCTTT
31[149]	ACAGTTAGCGTAACGATCTAAAGT
31[156]	CTGTATTTGTATAGCGTCAGCGATAGCA
33[131]	TTTGTCGTCTTTCAATAGGAA
33[151]	TAGTAACATTTATACCAAGCGC
35[141]	GGATTTTGCTATAGAAAGGAACAACTAAAGGA
35[156]	ACTTCACTACGAATACACTAAAAGAGGAAGGGAACCAGCGTCCAATACT
37[131]	ATTGCGAATAATAGTGTATCA
37[151]	CACGTTATGAGTTTCCATTAAA
38[149]	TCCAAACGGCTACAACAGCATCCACCAGA
39[141]	AAGGCTCCAAAAGGAGTAAAGCG
40[119]	TGAATTTCCGCTGAGGCTTGCAGGCAACTTTA
40[149]	TTGTATTTGCGGGATCGTCACCGATAGTAAATTGGGCTTAGAAAGA
42[155]	AAAGGAGGCTTTTAAGGCTTTAACAAAGTATCATAACCCTC
42[168]	AACATGAGCAGTACCGACAATAAACAAGTGCC
43[136]	TTTTGGTAGCAAAAA
43[167]	AAAGACAGGGACGACGACAAAAGGTCACCCAG
44[165]	TGAGGATTCAGCTATTCAGCGGCCAGAGGCGT

45[147]	CGGGTAAAATACGTTACAAGATTCATGGTAAACCAAACAGAGGGGTAAGAAAGA GCCCCAGGAAG
45[170]	AAATTCCAATAGATATGCAGAAGAAAGGGTTG
46[167]	TATGCTGCTCAACAGTTAATTTACACCCTCA
47[136]	TTTTCGAAAGAGATG
47[167]	CATCTTGGATCCCATCCAAGTCCTGATTCTAAG
48[165]	CAGCGAGTAGAAACACAGACAGCGTTTTTATT
49[147]	GAAACAAAGTACAGCCGGAACCCGCGACCGCTT
49[170]	TGTGATTTATCATTCAATCAATCAACCACCCT
50[168]	GAAATAGAGAGCATTCCAAGTTACCATCTTACC
51[139]	ATTGTGTCGAAATGAGGCGCAGAC
53[141]	GGTCAATCATACAGATGAAAGTTTTGCATAGCGAGGCGAACC
55[131]	AGGCGCATAGGCTACCTAAAA
57[131]	TATTCATTACCCAAATCAACGGCCCTGACCATA
59[141]	AAAGGAACGAGGGCCGCTTCGGTTTAT
59[151]	ACGAGTAGCTTTAGGAACAAA
60[119]	ATCATTGTAAAACGAACTAACGGACTAAAGTACGGTGTCTTTCGCAAA
60[135]	TTTAATTTGAGTTAAA
61[152]	TTCATCAGGATCTGTATAATGTATAAAAGGTGGCATC
62[168]	CGTCGCAGATTAGATTATCAGTGAAGAGGACT
63[136]	TTTTTGCAGATAGAG
63[148]	ACGCTACCACATGCTGAATAGCTCAACATTTTCATT
63[156]	AGGATCTGATAACTTTTGAAATACAGGCGCCT
63[167]	GGCATACAAAATTTATCAGACGCTGCAACGCC
64[165]	CAACACCTGCTCATCCTCCGGCTAAGTTATAC
65[147]	GTTTACCAGACGACAGGAAGCAA
65[170]	TGAATTCTTTTTAAAAAATCATAGTTTTTTCA
66[168]	TAACAATAATGGGTTATCAACTTTGAAACACT
67[167]	TAAAATTGAAAATCCAAATAACTATTAGTATCA
68[165]	CTGGATGAACTGACGTTACTTAACGCCGACCG
69[147]	GCGGAATCGTCATGACTATTAAATCAAAAAATG
69[167]	CATTGATTCACTTTTTCTCGCAAGAACCTGACCCC
70[155]	TAAAGAAACCATCACCAGTA
70[165]	TCAAATTGCTCCATCTGGCATGAGAAGGAA
71[139]	GAGAATGACCATATAGTCAGATTTAGAACTATTTCAAATATTCA
75[151]	AGACCGAAAAGCTAAATCGGTT
76[149]	ACTCCAGCAATAAAAAGGCAAAGAATCGA
77[131]	AGAGAGTACCTTTAATTGCTCGAGGTCATTTTTGCGGATGGC
79[131]	TTAGAGCTTAATTTCAACTAAATTACAGGTGAGATGG
81[120]	TGGTCAATAAACAGGAAGATTGTATTTTAACCAATAGGAA
81[136]	TAGCTATATGTTTTAAATATGCAAACAACATT
81[152]	TGGGGCGCGCGACCCCGG
82[168]	GAACAACTGCAGATGATATTATACTATTACGA

83[147]	AATTCTACTAATAAGAGTAATCGTAAAACTAG
83[167]	CATTAATAATTGTTTGGGGCAATTCTGTAAAT
84[165]	TAAATCTAATGGAAACGTAAAACGATTTCATT
85[156]	CAGAAAGGCTATGTAGCTATGCGCATCGTAACC
85[170]	GACAACTTATTTGCGGGTTAGAAATGTAAGAG
86[168]	TTACAATAATAAAGAAAATATACAGGTAATAG
87[147]	GTACCAAAAACATAAGCTAGCTGATAAATTAA
87[167]	CTGTAATAGTCAGATGATTGCGTAGTTACATT
88[155]	CCTTCCTCATATAGGGTGAGGTAATGTGCCAG
88[165]	GCGGGATACCTTTTTTTACCCTAAATATT
89[136]	TAAAAATTAGCAAAGCGGATT
89[170]	AGGAGCTCGCCTGAACATCGGGTGAGTTTAGA
90[155]	CAATAGCAAAATGTGAATTA
90[168]	CTAAAATTAATCAGGTCATACATAAATTAAGAC
91[149]	TGAAAAGGCCGGCACCGCTGATCGCACCAGTGAGGAATCCTGA
93[131]	CACCATCAATATGCGCAAGGA
93[151]	ACCGTTCATCTCAGGAATCTGGTGCTTGATTAGAAACTATC
95[141]	TGCCGGAGAGGCAGGTCATTAGG
97[131]	GGAGCAAACAAGAGAATTAGC
97[151]	TGAACGAGATGGGAACAGTTGGTGTGGTTGCTTGAATCAGA
101[104]	AGCTCATTTAAGCAAATATTTAAATGACCATT
101[128]	CGCCATCAAAAATAATATCAGAAAAGCCCCAAAACCTGTT
102[168]	ACGTGGAGCTGATAGCCCCACCAGCGTAGTAG
103[147]	CATTAAATGTGAGGGAGCGGGTGCGCGTA
103[167]	CAACCCAGACGAACGAACTAAAACATTTTGCG
104[165]	TCTCCGTAAAACAGGATCTACAGCAACAATTC
105[136]	TAATGGGATGCCTGAGAGTCT
105[148]	TCACAACGGCGGGGTCACGCCGCTAGGG
105[170]	ACACGACGCCTGCAAGGTGAGGTATCATCCAA
106[168]	AGATTCTGGTTTTGAGAAAAATCTATATGACC
107[147]	GTGCATCTGCCAGTACGCCAGCCACCGAG
107[167]	GGACGAACGGCAAATGAACAGTGCCTTAGACT
108[165]	ATCGGCCACCTTGCGATTCAAAATTTATCTTT
109[170]	ACAGGAATCAATATTGAACCTCCAATACTTTT
110[119]	TGGGAAGGTAACGCCAGGCCAGTGCCAAGCTTCTGTGTGA
110[135]	CAGGCTGCGCAACTGT
111[136]	CTGAGTAGTCGCCATTGCTTTCCGGAGACAGTCAAAT
111[160]	GGCCTTGCAGGGCGACCACAATCA
112[143]	TCACTTGCTTTATAATTCCAGCCA
113[112]	AATTGTTAGAAGCATAAAGTGTAAACCTGTCGTGCCAGCTGCGGTTTG
113[120]	TCCGCTCACAATTCCAGACGTTGTAAAACGACGGGTTTTCCCAGTCAC
113[160]	TAAAAGAGATACTTCTCGGCATTGCA
114[143]	GAAGTGTTCGTGCTTTCCTCGTTATGACGAGC

115[160]	GCGGGAGCAGGAACGGTTTGAGG
116[111]	CGTATTGGAGTTGCAGCAAGCGGTTGTTTGATGGTGGTTCGGTGCCGT
116[135]	CAACGCGCGGGGAGAGGCATTAATGAATCGGCCACAACATACGAGCCG
116[143]	ACGTATAAAGTGTAGCATTGACCG
117[160]	ACCACCACCGTACTATAGAACCAGTC
118[143]	CGCTGGCAGGGAGCCCCCGATTTA
119[112]	AAAGCACTAGTTTTTGGGGTCGAACCATCACCCAAATCA
119[120]	AAATCGGAACCCTAAACAGCAGGCGAAAATCCCCACGCTGGTTTGCCC
119[152]	GAGCTTGACGGGGAAAAAGCGAAACGAGTAA
99[141]	CATGTCAATCATATGTAACCAGCTTTCATCAA

0[186]	TTTTTACCAGCGCCAAAGACAAATGGTAATATCCAGTTTT
2[186]	TTTTAGACACCACGGTTCATATGGTTTTTT
4[186]	TTTTTAGCAAACGTAAAGAAACGCAATTTT
6[186]	TTTTAACGGAATACCACGCAGTATGTTTTT
8[186]	TTTTGTAAGCAGATAAACGCAATAATTTTT
9[168]	GAAGCCCTTGAAATAGCCCAATAATAAGAGCATTTT
10[183]	TTTTAGAAACAATTTTAAGAAAATTTT
12[183]	TTTTCTAATATCGTAGGAATCATTATTTT
14[183]	TTTTTTAGACGGAATCAGATATAGATTTT
16[183]	TTTTAAAATGAAGAACCTCCCGACTTTTT
18[183]	TTTTCAGCCATATTAAATCAAGATTTTTT
20[180]	TTTTCAACGCTAACGAGCGTCTTTCCAGATGGC
22[188]	TTTTAGTTGCTATTTTGAAAGTAATTTTT
23[167]	GTCGAGGCCTTATTTATCCAGTTACAAAATAAATTTT
24[188]	TTTTTGCGGGAGGTTTTCGCGCCTGTTTT
25[170]	TTTAGCAATAGCAGTTTTTGTTTAACGTCATTTT
26[188]	TTTTAGGCTTATCCGGTAACAAGAATTTT
27[167]	GAGCCAGCAGAGAATTAAAAACAGGGAAGCGCATTTT
28[188]	TTTTCCGCGCCCAATAGAATCGGCTTTTT
29[170]	TTCATCAGAGAGATAGAGGGTAATTGAGCGTTTT
30[191]	TTTTACCGCACTCATCGAGAGGGTATTAGTCTTTCCAAATAAGGCGTTATTTT
31[184]	AACCAAGTTTTTTTT
34[183]	TTTTAAATAATAATCATAATTACTATTTT
36[183]	TTTTTTTATCAATTACCAGTATAAATTTT
38[183]	TTTTTCTGTCCAGCTTAATTGAGAATTTT
40[180]	TTTTTTTCGAGCCAGTAATAAGAGAATTTTTATCCTGAATCTTACTTTT
42[188]	TTTTTCGCCATATTTAAAGAAGAGTTTTT
44[188]	TTTTGCCAACGCTCAACAGGTCTGATTTT
46[188]	TTTTGAAAAAGCCTGTTATGTAAATTTTT
48[188]	TTTTAATAAGAATAAACCAAAGAACTTTT
50[191]	TTTTTCTGACCTAAATTTATTTAGTTAGCGAGAAAATTTCAATTACCTTTTT
51[184]	ATTTCATCTTTTTTTT

54[183]	TTTTGCTGATGCAACAAACATCAAGTTTT
56[183]	TTTTGAGACTACACCTTTTTTAATGTTTT
58[183]	TTTTCAATAGTGTATATGTGAGTGATTTT
60[180]	TTTTTAGAATCCTTGAAAACATAGCCTCTAATTTAGGCAGAGGCATTTTT
62[188]	TTTTATAACCTTGCTTCATCAATATTTTT
64[188]	TTTTGAAACAGTACATAACCTACCATTTT
66[188]	TTTTAAAACAAAATTAAATTTTCAGTTTT
68[188]	TTTTGAGCAAAAGAAGAAGAAACAATTTT
70[191]	TTTTATCGCGCAGAGGCGAAAATACCAATAACGGATACTAACAACTAATTTTT
71[184]	GTTACAAATTTTTTTT
74[183]	TTTTGTTTAACGATAATACATTTGATTTT
76[183]	TTTTTATCAAAATCGTATTAAATCCTTTT
78[183]	TTTTAATCCTGATTTTAAAAGTTTGTTTT
80[180]	TTTTCGGAATTATCATCATATTCCTTTGTATTAATTAATTTTCCCTTTTT
82[188]	TTTTAGTAACATTATCATCGCCATTTTTT
84[188]	TTTTTTTGCCCGAACGTCGGTCAGTTTTT
86[188]	TTTTGGATTTAGAAGTAACGCTGAGTTTT
88[188]	TTTTAGATTAGAGCCGTAAATATCATTTT
90[191]	TTTTTGAAAGGAATTGAGGATTGGCAAAAACCCTCAAAAACGCTCATGGTTTT
91[184]	TCAACAGTTTTTTTTT
94[183]	TTTTAGCCAGCACTCAATCGTCTGATTTT
96[183]	TTTTATTAACACCCAGTAATAAAAGTTTT
98[183]	TTTTAAAAATACGATAGAACCCTTCTTTT
100[149]	TTGATATCGCGTCTGGCCTTCCTGTCACAGACAATATTTTTGATTTT
100[180]	TTTTATGGCTATTAGTCTTTAATGCGAGAGAAACCACCAGAAGGAGTTTT
102[188]	TTTTTGACCTGAAAGCGACGTGGCGTTTT
104[188]	TTTTGGACATTCTGGCCCGCTTAATTTTT
106[188]	TTTTAATGGATTATTTAAGGCCGATTTTT
108[188]	TTTTAAATACCTACATTTCACGCAATTTT
110[180]	TTTTAACAATATTACCGCCAGCAAATAGGTAAATATTTTGTAGGTGGCA
112[183]	TTTTATTAACCGTTGTAGCATCTGTCCATTGCGACAGT
114[183]	TTTTTAAAGGGATTTTAGACTAAACAGGCATTGGC
116[183]	TTTTGCGCCGCTACAGGGCGACCCGCCGAACGTCGGAT
118[183]	TTTTAGAAAGGAAGGGAAGAGCCGGCGATAAGAAT

DS-linker strands

0[135]	GTATACCGCCACCCTCAGAACCGC
2[135]	GAGTATTAAGAGGCTGAGACTCCTCACCGTACTCAGGAGGTTTAGAAT
4[135]	CAACGTCATACATGGCTTTTGATGTATTATTCTGAAACATGAAAGCCA
6[135]	CGAAACCACCACCAGAGCCGCCGCTGAATTTACCGTTCCAGTAAGGGC
8[135]	TTTTAGCCCCCTTATTAGCGTTTGCACCCTCAGAGCCGCCACCAGCAG
10[134]	GGAAGTAGCACCATTACCATTAGTTTCATCGGCATTTTCGGTCACGG
12[133]	CGCGCGACATTCAACAAAATCACCACCA

14[133]	GCCAAAATACATACAAGACAAAAGGCAC
16[133]	TATAAGCAGATAGCAGCAAACGTAGGCC
18[133]	TGAGTAATTGAGCGTTAAGAAAAGTTCA
20[77]	CAGAACGTCAAAAAT
20[133]	GTACTGGTAATAAGAAAGTCAGAGGATT
21[72]	TTTTTAATGCCCCCTGCCTATTTCCGATAGTTGCGCCGACAATGACTG
60[78]	TTATCAATATATGTGGTAAAGTAATTCAAC
61[72]	AATACCAGTCAGGACGTTGGGAAGATAACAGTTGATTCCCAATTCAGC
100[77]	TGTATACCTACATTATATCTTTAGGTGC
101[64]	CGCTCATGGAATAAAA
110[87]	ATTACGCCAGCTGCTA
111[72]	GAGGCGAAAGGGGGATACTCTAGAGGATCCCCGGGTAGTA
113[64]	CGCCCGAGCTCGAATTCGTAATCATGAGTGAGCTAACTCACATTACAC
115[64]	GGTATTGCGTTGCGCTCACTGCCCTCTTTTCACCAGTGAGACGGGGGA
117[64]	AAACAACAGCTGATTGCCCTTCACCCTTATAAATCAAAAGAATAGAGG
119[64]	TAACCCGAGATAGGGTTGAGTGTTGAACGTGGACTCCAACGTCAACAA
121[56]	**T**GAACCATCACCAGG

DS-probes. The red- and green-colored portions of the sequences are complementary to the ssDNA conjugated to the enzymes, and are located in the Left and Right half-cages, respectively.

64[71]	ATTCATTTCAATTACCCGCGCAGAGGCGAATTTTTTGGAGGGAGGG
74[76]	TCAGATGATGGCAACAATAACTTTTGGAGGGAGGG
76[66]	ATTATCATTTTTTATCATCATATTCCTGATTATTTTGGAGGGAGGG

34[149]	TTCTGTGCAAAAGAAGGCACCAGGCTGACCGTAATCTTGACAAGAACCGGATTTTC CAGCCAGCC
67[136]	GCAAAAGACGGTGTACAGACCTTTTCCAGCCAGCC
73[131]	GCATCAAAAAGATTAAGAGGAACTTCAAATATCGCGTTTTAATTTTCCAGCCAGCC

Supplementary Methods.

Enzymes and substrates: Glucose-6-phosphate dehydrogenase (G6PDH, *Leuconostoc mesenteroides*), malic dehydrogenase (MDH, *porcine heart*), lactate dehydrogenase (LDH, *rabbit muscle*), glucose oxidase (GOx, *Aspergillus niger*), horseradish peroxidase (HRP) and β-galactosidase (β-Gal, *E. coli*) were purchased from Sigma (St. Louis, MO). Pyruvate, oxaloacetate (OAA), glucose 6-phosphate (G6P), glucose, resorufin β -D-glucopyranoside (RBG), β -nicotinamide adenine dinucleotide (NAD), resazurin (RESA) and phenazine methosulfate (PMS) were obtained from Sigma-Aldrich. ABTS (2,2'-Azino-bis[3-ethylbenzothiazoline-6-sulfonic acid] diammonium salt) was purchased from Pierce (Rockford, IL), polyphosphate (100) is ordered from Kerafast.

DNA strands: Single-stranded M13mp18 DNA was purchased from New England Biolabs. Staple strand oligonucleotides were obtained from Integrated DNA Technologies (IDT) on 96-well plates and used without further purification. Thiol-modified DNA oligonucleotides were also purchased from IDT, and were purified by denaturing PAGE before use.

Crosslinking reagents: *N*-Succinimidyl 3-(2-pyridyldithio)propionate (SPDP) and tris(2-carboxyethyl)phosphine (TCEP) were obtained from Pierce. Dimethyl sulfoxide (DMSO) was purchased from Sigma.

Buffers: Phosphate buffered saline (PBS), HEPES sodium salt, Tris buffered saline (TBS), Tris base, acetic acid, EDTA, and magnesium acetate were purchased from Sigma. 1×TAE/Mg^{2+} buffer (pH 8.0) is prepared by 40 mM Tris, 20 mM acetic acid, 2 mM EDTA and 12.5 mM magnesium acetate.

Dye-labeling reagents: NHS-Cy3, Cy5 amine reactive dyes were purchased from GE Healthcare Life Sciences. NHS-AlexaFluor®555 and AlexaFluor®647 amine reactive dyes were obtained from Life Technologies.

Amicon centrifugal filters were purchased from Millipore.

PEG 8000 was purchased from Promega.

Surface PEGylating reagents: APTES (3-Aminopropyl)triethoxysilane was purchased from Sigma-Aldrich. mPEG-SVA 5k and biotin-PEG-SVA 5k were obtained from Laysan Bio, Inc.

TEM imaging: TEM grids (400 mesh, copper grid coated with ultrathin carbon, Ted Pella) were glow discharged (Emitech K100X). 2 µl concentrated samples were deposited onto the grids for 1 min, washed with 10 µl DI water for 5 sec, stained with 10 µl 1% uranyl formate twice (2 sec for the first time and 15 sec for the second time), and imaged using Philips CM12 transmission electron microscope.

Enzyme activity assay: A 96-well-plate reader was used to monitor enzyme activity through absorbance changes of the samples. The enzyme samples and substrates were loaded in the wells of the 96-well plate with a final concentration of caged enzymes ~ 0.5 nM in 1× TBS (Tris buffered saline with 1 mM $MgCl_2$, pH 7.5) for most assays. The DNA cage concentration was determined by the A_{260} value as described above. For a typical GOx and HRP asasy, 1 mM Glucose and 2 mM ABTS was used as substrate and enzyme activity was measured by monitoring the increase in absorbance at 410 nm ($ABTS^{-1}$). For a typical G6pDH assay, 1 mM G6P and 1 mM NAD^+ were used as substrates, and enzyme activity was measured by monitoring the increased absorbance at 340 nm due to the reduction of NAD^+ to NADH. For a typical LDH

assay, 2 mM pyruvate and 1 mM NADH were used as substrates, and enzyme activity was measured by monitoring the decreased absorbance at 340 nm due to the oxidation of NADH to NAD^+. For a typical MDH assay, 2 mM OAA and 1 mM NADH were used as substrates, and enzyme activity was measured by monitoring the decrease in absorbance at 340 nm. For a typical β-Gal assay, 100 μM RBG was used as substrate and enzyme activity was measured by monitoring fluorescence intensity, with excitation at 532 nm and emission at 590 nm.

Trypsin assay: Enzyme activity was measured after incubation with or without trypsin (1 μM) at 37 °C for 24 h in 1×TAE-10mM Mg buffer (pH 8.0). Activity assay conditions: 1 mM Glucose, 1 mM ABTS, 1 nM of free GOx and HRP in pH 7.5, 1×TBS buffer containing 1 mM $MgCl_2$, and monitoring absorbance at 410 nm. In the DNA cage experiment, all conditions were the same except for incubating 1 nM DNA cage-encapsulated GOx and HRP with trypsin.

Supplementary References

1. Fu, J., Liu, M., Liu, Y., Woodbury, N. W. & Yan, H. Interenzyme Substrate Diffusion for an Enzyme Cascade Organized on Spatially Addressable DNA Nanostructures. *J. Am. Chem. Soc*. **134**, 5516–5519 (2012).
2. Liu, M., Fu, J., Hejesen, C., Yang, Y., Woodbury, N. W., Gothelf, K., Liu, Y. & Yan, H. A DNA Tweezer-Actuated Enzyme Nanoreactor. *Nature Commun.* **4**, 1-5 (2013).
3. Abelson, J. *et al.* Conformational dynamics of single pre–mRNA molecules during in vitro splicing *Nat. Struct. Mol. Biol.* **17**, 504-512 (2010).
4. Michelotti, N. *et al.* A bird's eye view tracking slow nanometer-scale movements of single molecular nano-assemblies. *Methods Enzymol.* **475**, 121-148 (2010).
5. Blanco, M. & Walter, N. G. Analysis of Complex Single-Molecule FRET Time Trajectories. *Method. Enzymol*. **472**, 153-178 (2010).
6. Gourévitch, B. & Eggermont, J. J. A nonparametric approach for detection of bursts in spike trains. *Journal of Neuroscience Methods* **160**, 349-358 (2007).
7. Rinaldi, A. J., Lund, P. E., Blanco, M. R. & Walter, N. G. The Shine-Dalgarno sequence of riboswitch-regulated single mRNAs shows ligand-dependent accessibility bursts. *Nat. Commun., 8976* (2015).
